# The Subnational Corruption Database: Grand and petty corruption in 1,473 regions of 178 countries, 1995–2022

**DOI:** 10.1038/s41597-024-03505-8

**Published:** 2024-06-25

**Authors:** Lamar Crombach, Jeroen Smits

**Affiliations:** 1 KOF Swiss Economic Institute, ETH Zürich, Leonhardstrasse 21, 8092 Zürich, Switzerland; 2 Global Data Lab, Economics, Institute for Management Research, Radboud University, Nijmegen, Netherlands

**Keywords:** Society, Government, Sociology, Politics, Economics

## Abstract

This data descriptor presents the Subnational Corruption Database (SCD), which provides data on corruption in 1,473 subnational areas of 178 countries. The SCD includes a comprehensive overall corruption index, the Subnational Corruption Index (SCI), and its two components: the Subnational Grand Corruption Index (SGCI) and Subnational Petty Corruption Index (SPCI). The SCD is constructed by combining data of 807 surveys held in the period 1995–2022 and includes the corruption experiences and perceptions of 1,326,656 respondents along 19 separate dimensions. The data are available for multiple years, allowing longitudinal analyses. At the national level, the SCI correlates strongly with established corruption indices, like the Transparency International Corruption Perceptions Index (CPI) and the World Bank Control of Corruption Index (CCI). We create subnational estimates of the CPI and CCI by superimposing the subnational variation of the SCI around the national averages of these indices. The presentation of subnational data in the SCD and the separation between grand and petty corruption significantly broaden the global knowledge base in the field of corruption.

## Background & Summary

Corruption remains a persistent global issue. According to Transparency International, 152 out of 180 ranked countries have made no notable progress in reducing corruption since 2012, and in some the situation has even worsened^1^. This trend yields pervasive negative consequences, as research has shown that corruption may lead to: (i) increased bureaucratic inefficiency, (ii) deterioration of the investment climate, (iii) reduced civil and political rights, (iv) diminished levels of economic growth and foreign investment, (v) exacerbated poverty and income inequality, (vi) reduced international trade, (vii) compromised political legitimacy of the state, (viii) larger shadow economies and thus reduced tax bases, (ix) higher levels of brain drain, (x) larger fiscal deficits, and (xi) poorer education, health and socioeconomic outcomes^2,3^. Given these detrimental effects, the pervasive nature of worldwide corruption is indeed a cause for concern.

To develop effective strategies to reduce corruption and mitigate its negative effects, comparative research on a global scale is of utmost importance. Comparative research enables the identification of factors related to corruption, while simultaneously highlighting the unique contexts in which corruption may be more or less relevant^3^. To facilitate this type of research, the existing literature predominantly makes use of two readily available indices: Transparency International’s Corruption Perceptions Index (CPI)^1^, and the World Bank’s Control of Corruption (CCI) index^4^. The CPI relies on the assessment of experts and business executives to gauge corruption in the public sector, while the CCI combines data from household and firm surveys, commercial business information providers, non-governmental organizations and public sector organizations.

These indices have proven to be very useful for comparative research on the causes and consequences of corruption^5–7^. However, a disadvantage of these indices and thus of existing comparative research is that they are only available at the national level and may therefore hide existing variation of corruption within countries. There is broad evidence that regions within countries may differ in many respects, including socioeconomic, demographic, health and cultural outcomes^8–14^, which are all characteristics known to be related to corruption^2,3,14^. It is therefore not surprising that the few studies focusing on within-country variation in corruption identified substantial differences in this respect^15–18^. These findings make it clear that country-level data is inadequate for gaining an encompassing understanding of the causes and consequences of corruption.

However, until now only a few studies have collected within-country data of corruption in a comparative manner. One study^15^ brought together subnational data for 27 EU countries in 2009, while another study^16^ did so for 22 African countries in 2013–2015. The most extensive of these studies^17^ assembled data from barometer surveys, enterprise surveys and a broad range of other sources to build a database that includes subnational corruption for 101 countries in 2005. This database is impressive and constitutes a first important step into the direction of a global subnational corruption database. Nevertheless, it has some disadvantages. The data has become rather outdated, is restricted to only one point in time (around 2005), and many – in particular low-income – countries are not included.

The aim of the current data descriptor is to present a dataset with information on corruption at the level of subnational regions that solves these issues. This Subnational Corruption Database (SCD) contains national and subnational values of the Subnational Corruption Index (SCI) and its two equally weighing components: the Subnational Grand Corruption Index (SGCI) and the Subnational Petty Corruption Index (SPCI). It offers data on 1,473 regions within 178 countries between 1995–2022. The SCD improves on the previous study’s^17^ database in several important ways. First, we have extended the number of countries from 101 to 178, whereby in particular low-income countries have been added. Second, we include data for several points in time, such that changes in subnational corruption over time can be studied. Third, we present, besides an overall corruption index, two additional subindices that represent the two major underlying forms of corruption: grand corruption and petty corruption. Fourth, our method of harmonization between surveys ensures that regions and countries can be assessed on their corruption levels in a comparable manner, as our method maximizes conceptual consistency between the surveys.

The SCD provides researchers worldwide with a set of global corruption indices that reveals in unprecedented detail how corruption is distributed across the world and how it varies both within and across countries and global regions. The SCI can be included as a dependent as well as an independent variable in explanatory studies that aim to increase our understanding of the roots of corruption and the effects it may have on economic, social and development-related processes. In the political arena, the SCI may contribute to the realization of the global development agenda, where corruption is explicitly highlighted under SDG targets 16.5 “Substantially reduce corruption and bribery in all its forms” and 16.6 “Develop effective, accountable and transparent institutions at all levels”^19^. By providing localized information on corruption, our database aims to facilitate more effective, targeted anti-corruption strategies, enrich the academic discourse on corruption, and offer a tool to policymakers, researchers, and activists seeking to understand and combat corruption at various governance levels. This work may ultimately contribute to a comprehensive, layered understanding of corruption, advancing our collective ability to address this pervasive issue.

## Methods

In line with Transparency International, we define corruption as “The abuse of entrusted power for private gain”^20^. Transparency International also distinguishes between the two sub-forms of corruption. They define “petty corruption” as the everyday abuse of entrusted power by public officials in their interactions with ordinary citizens who try to access basic goods or services. Further, they define “grand corruption” as the abuse of high-level power, that benefits the few at the expense of the many and causes serious and widespread harm to individuals and society^20^. In the SCD, subnational and national data on all three forms of corruption (grand, petty and total) are presented, whereby the grand and petty corruption subindices (SGCI and SPCI) are first estimated separately, and the total corruption index (SCI) is computed as the (unweighted) mean of both subindices. The two subindices constitute the first empirical attempt in the literature to separate these two concepts in a comparable manner for a global sample. Because the SGCI and SPCI are developed independently, researchers can also use these indices to assess the relationship between petty and grand corruption.

To construct a global corruption index, one requires data that is comparable over time and between (sub)national areas. Ideally, one would have access to an annual global survey which asks the same questions on all relevant aspects of corruption to a representative sample each year. However, the reality is another. Although there are many surveys asking a broad range of corruption questions, these surveys are held irregularly, and the questions may change over time and differ between survey programs. As such, while there is ample information on all aspects of corruption, this information is scattered, inconsistent and unstandardized. Thus, the main challenge we faced was to combine, standardize and harmonize the many and diverse pieces of information into one coherent picture of the variation in corruption across time and place.

We are not the first to face this challenge. Transparency International and the World Bank faced this issue when creating their national corruption indices. The same holds for other organisations or scholars that focus on concepts other than corruption, like the Institute of Social Studies (ISS) when creating their Indices of Social Development^21^, and for the subnational version of the Human Development Index (SHDI)^22^. The methods used by these and other experts to combine data fragments derived from many different sources into a comparative global picture are based on the assumption that each data fragment contains information on a certain aspect of the underlying construct that is measured with error^4,20,21^. To separate this information from the error, these organizations start with recoding all variables in such a way that they have the same form (CPI 0–100; CCI and SHDI 0-1; ISD standardized) and that higher values indicate a more positive outcome.

Transparency International then imputes the missing values on the variables, turns them into z-scores, normalizes them to a 0–100 scale and takes the mean of the subindices as the final CPI score. By computing z-scores using the mean and standard deviation in a baseline year, they achieve comparability over time from 2012 onwards^20^. Alternatively, The World Bank uses an Unobserved Components Model to ensure comparability between the variables derived from diverse sources. They then create a weighted average of these variables as the final index. With this method comparability over time is achieved for the relative position of countries on the scale, but not for their absolute positions, which reduces the possibilities for trend studies^4^. The ISS uses a variant of the matching percentiles methodology^23^ which assigns scores to countries based on ordinal rankings. The SHDI^22^ achieves consistency across different sources by normalizing the information on subnational variation in subindices around the official national values of these indices in the Human Development Index database (https://hdr.undp.org/data-center).

We follow a different approach. While the CPI is completely and the CCI largely based on the judgments of experts and business executives, the SCI is almost completely derived from surveys in which representative samples of the population are interviewed regarding their personal experiences with and perceptions of corruption. Given that these surveys are rather diverse with respect to data availability and questions on corruption, it was a challenge to bring together all information under a common denominator. To address this challenge, we developed a new approach, which will be discussed in detail in the next sections.

### Data

The data used to compute the subnational corruption indices are derived from 13 multi-country survey programs, which are presented in Supplementary Table 1. The programs are Afrobarometer (2008, 2012, 2014–2021), Arab Barometer (2011, 2014, 2017, 2021), Asian Barometer (2010, 2014–2016, 2018, 2019); Eurobarometer (2007, 2011, 2017, 2019, 2021), European Quality of Government Database (2010, 2013, 2017, 2021); International Social Survey Programme (2006); LAPOP AmericasBarometer (2004, 2006–2010, 2012, 2014, 2016–2019), Latinobarómetros (1998, 2000, 2001, 2013, 2016–2018), Global Corruption Barometer (2017, 2019), The Asia Foundation (2006–2019); World Values Survey (1995–1999, 2011–2014, 2017–2020); World Bank Country Opinion Survey (2012–2021); World Bank Enterprise Surveys (2009, 2011–2013, 2016, 2019)^24–31^. In total, our database includes survey data on 1,326,656 individuals derived from 807 national surveys, 769 of which contain information on grand corruption, and 589 on petty corruption.

The vast majority of these programs interview representative samples of country populations on a broad range of topics, including their experience with and awareness of corruption. The last two programs differ in that the World Bank Country Opinion Survey is based on interviews with experts and stakeholders of the World Bank Group and the World Bank Enterprise Survey is a firm-level survey using a representative sample of the country’s enterprises. Given that we aim to construct indices that represent the perspective of a country’s general population, the last two sources are only used to include countries for which no other usable subnational information is available (36 out of 807 surveys). When necessary, weighting variables were used to create representative samples of a country’s population.

The 13 data sources together contain a very broad range of questions on different aspects of governance, of which 103 questions were considered relevant for the construction of our database. This large number of relevant questions reflects the fact that corruption can take many different forms and be experienced in many ways, but also the broad variation in measurement approaches between different survey traditions. Questions are included about the number of persons involved in corruption, the degree to which institutions are involved in corruption, the prevalence of corruption in the country, experience with being asked for a bribe for getting a service, to what extent voters are bribed, etc. The answering possibilities varied between simple yes/no variables, a few categories (like none, some, most, all), more categories, or ranges from 0–10 (low to high) or 0–100 (percentages involved), etc. Supplementary Table 2 presents all questions, with their original answering categories and the surveys in which they were asked, while Supplementary Table 3 offers information on the coverage of countries and dimensions, and the number of observations by survey.

### Approach

As a first step towards reducing the vast and diverse information set to manageable proportions, we follow Transparency International and the World Bank by recoding the variables from our raw data sources in such a way that they all have the same range – in our case 0-1 – and that higher values indicate less corruption. In this transformation step, a variable with two categories obtained values 0 and 1, a variable with four categories (e.g. 0 None, 1 Some, 2 Many, 3 All) obtained values 0, 0.33, 0.66, 1, and a variable running from 0 to 10 the values 0, 0.1, 0.2, ….. 0.9, 1.

These are linear transformations whereby the assumption is made that the variables are of interval measurement level. Given that most of the included variables are Likert type scales, for which the response categories are symmetric in nature, this assumption is not expected to lead to serious problems in the Principal Component Analyses (PCA) that are used to create our corruption indices. There is broad evidence^32–34^, even going back to Pearson himself^35^, that analyses based on Pearson correlations, like regression and PCA, are highly robust against violations of the underlying assumptions. Using Likert type items in these kind of analyses has therefore become common practice in social research^33,34^.

**Fig. 1:**
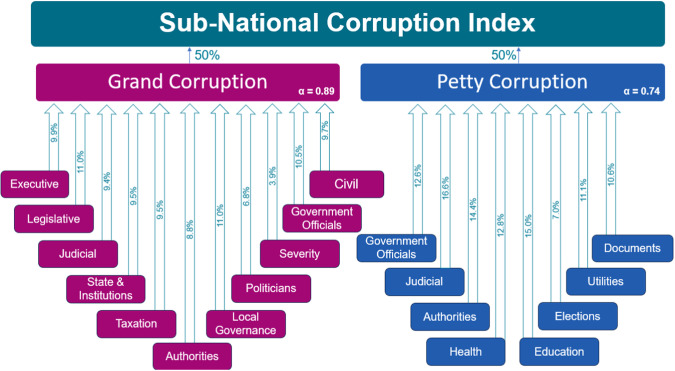
Dimensions of corruption and their relationships with grand and petty corruption.

After this recoding step, the 103 questions were assigned to one of 19 different dimensions of corruption, of which 11 dimensions reflect aspects of grand corruption – the perceived abuse of high-level government power–and the other 8 aspects of petty corruption – the experience with abuse of power by public officials in the form of bribery. As a result, we obtain values for up to 19 dimensions for the 1,326,656 million individuals in the dataset. Figure 1 summarizes these dimensions and their relationship with the corruption indices. Table 1 summarizes the absolute weights in table form.

**Table 1:** Component weights of the subdimensions of grand and petty corruption.

Grand Dimension	Weight	Petty Dimension	Weight
Executive	0.3214	Government Officials	0.3497
Legislative	0.3504	Judicial	0.4349
Judicial	0.3080	Authorities	0.3901
State & Institutions	0.3085	Health	0.3601
Taxation	0.3125	Education	0.4011
Authorities	0.2918	Elections	0.2139
Local Governance	0.3504	Utilities	0.3220
Politicians	0.2239	Documents	0.3104
Severity	0.1371		
Government Officials	0.3307		
Civil	0.3161		

The 11 dimensions for Grand corruption include: perceived corruption in the executive (President, Prime Minister and their immediate officials), legislative (Parliament or Congress), or judicial domain (judges, courts and magistrates), in state and institutions (state institutions and agencies), among politicians (individual politicians and political parties), tax authorities (tax officials, ministries of finance and tax collection agencies), government officials (such as public employees providing building permits), authorities (law enforcement agencies, particularly the police force), local governance (municipal, provincial) and overall corruption severity perceptions (intensity and frequency of corrupt practices in general).

The 8 dimensions of petty corruption include: bribery experiences when dealing with government officials (in exchange for services or expediting processes), the judicial (to receive necessary services from the courts or judicial service providers), authorities (to avoid legal issues or receive services from law enforcement or customs), healthcare (to receive medical care or assistance in clinics and hospitals), documents (to obtain necessary documents, such as permits, licenses, or other official paperwork), utilities (to access basic utilities such as water, sanitation, and electricity), elections (vote-buying and bribery in the context of elections), and education (in exchange for educational services or assistance, teachers and enrolment).

### Harmonization

Each of the 19 dimensions is based on a set of similar underlying questions, which are all summarized in Supplementary Table 2. The way of questioning can vary between the questions within a dimension. Such differences in semantics are relevant, because they can influence survey outcomes. For instance, language processing algorithms reveal that a substantial part of the variation in survey outcomes in organisational behaviour research may stem from semantic differences, and not from fundamental differences in the latent variable^36^. This might mean that regions differ in their SCI values not because of fundamental differences in corruption levels, but rather as a result of differences in formulation of the questions. Failure to adjust for such discrepancies would render the SCI less reliable.

To minimize the impact of such semantic differences between sources, we make use of the fact that the different sources overlap and that for a substantial number of countries questions are asked in several ways. Our strategy for using this overlap is as follows. For each dimension, we first pick a ‘semantic default option’, which is the semantic option that requires the least number of adjustments (see Supplementary Table 2), depending on the available set of questions. The goal, then, is to adjust all other questions within a dimension to reflect the difference in semantics from the default option. For instance, one frequent semantic difference was whether respondents were asked about the severity/extent of corruption, or rather the number of corrupt officials. When comparing the responses to such questions from different sources in the same country and the same time period, one consistently observes that questions on the number of corrupt officials are upward biased (higher mean; less corruption) and less volatile (lower standard deviation) relative to questions on the severity/extent of corruption. For instance, respondents are less likely to answer ‘almost everyone is corrupt’ relative to ‘[…] is extremely corrupt’ even if the questions themselves are comparable in nature. This is problematic, because both of those categories receive the same numerical value.

We exploit the known differences in means and standard deviations between sources within the same country and time period to adjust responses depending on their semantics. More specifically, if one asks two representative samples of the Greek population in a given time period on the number of corrupt state officials (World Values Surveys) and the extent of corruption of state officials (Eurobarometers), one would expect a very similar mean and standard deviation in the case of no impact of the semantic difference, as both questions are meant to capture the same corruption dimension. That is, if both questions were interpreted similarly by the respondents of these two Greek representative samples in the same period, there should be no difference in the outcomes. When such differences did exist, we adjusted the responses of one source to be more comparable with the other source by equalizing the means and standard deviations using the following formula:

$${Q}_{i}^{Adj.}=\frac{({Q}^{Orig.}-{\mu }_{i}^{Orig.})}{{\sigma }_{i}^{Orig.}}\,\ast \,{\sigma }^{New}+{\mu }_{i}^{Orig.}+{\mu }^{New}-{\mu }^{Old.},$$

where the adjusted value of question *Q* of respondent *i* is a result of multiple transformations. First, the original question value was standardized within each country-year, such that the average is 0 and the standard deviation is 1. We then multiplied each value with the new standard deviation, which is the standard deviation of the question with the default semantics we were aligning with. We then added back the original country mean and accounted for the difference in means between the original question and the semantic default question for the set of overlap countries by adding the difference in means of the set of overlap countries between the source with the default semantic option (*μ*^*New*^) and the other question (*μ*^*Old*^). This allows for comparability between countries over time without touching the overall variance structure within the country. Countries that were part of a set of overlapping countries received a full adjustment based on their own idiosyncratic differences in means and standard deviations (i.e., *σ*^*New*^, *μ*^*New*^, *μ*^*old*^ become country-specific ${\sigma }_{i}^{New},{\mu }_{i}^{New},{\mu }_{i}^{Old}$). Essentially, this approach allows us to estimate the semantic bias of one question design relative to another question design, which we then used to minimize this bias. Every single adjustment is explicitly described for each question in Supplementary Table 2. The code that performs these adjustments is available in the Stata do-file ‘From Raw Dataset to Baseline Dataset’ included in the SCD.

### Completing the database

In the harmonization step, we ensured that all variables measuring a specific dimension were conceptually similar and thus comparable. This minimized the issue of differences in measurement between the data sources, but it did not yet account for the fact that each survey offers data on a limited set of dimensions. This could be problematic, because each dimension only sheds light on one part of overall corruption. For instance, respondents are much more likely to report corruption of politicians than of the police authorities (see Table 4). Thus, if a survey on a certain area only measures the corruption of politicians, the area would appear more corrupt than if it only had data on the police authorities. This bias as a result of missing dimensions could lead to unfair comparisons between areas and over time.

This means that–similar to the World Bank^4^ and Transparency International^1^–we had to adjust for unobserved dimensions. We did this by using an imputation process that exploits the relationships between dimensions at the individual-level, obtained from surveys that cover multiple dimensions simultaneously. Tables 2 and 3 provide the correlation coefficients between the dimensions, all of which are significant at the 95% level. If there is no value, it means that there exists no survey where both dimensions were recorded simultaneously. The figures show that all but one dimension are well connected with the other ones. The only exception is the severity dimension, but given that three connections existed, we decided to keep it in our dataset.

**Table 2:** Pairwise Correlation Matrix Grand Corruption Dimensions.

	Executive	Legislative	Judicial	State & Institutions	Taxation	Government Officials	Local Government	Authorities	Civil	Politicians	Severity
Executive	1										
Legislative	0.69	1									
Judicial	0.50	0.53	1								
State & Institutions	0.35	0.56	0.38	1							
Taxation	0.47	0.51	0.61	0.31	1						
Government Officials	0.59	0.66	0.48	0.38	0.51	1					
Local Government	0.55	0.64	0.48	0.69	0.49	0.54	1				
Authorities	0.46	0.50	0.59	0.34	0.59	0.50	0.48	1			
Civil	0.50	0.56	0.50	0.48	0.52	—	0.61	0.52	1		
Politicians	0.66	0.72	0.61	0.70	—	0.63	0.62	0.53	—	1	
Severity	—	—	—	0.41	—	—	0.39	—	0.34	—	1

**Table 3:** Pairwise Correlation Matrix Petty Corruption Dimensions.

	Authorities	Government Officials	Judicial	Utilities	Education	Documents	Health	Elections
Authorities	1							
Government Officials	0.40	1						
Judicial	0.32	0.27	1					
Utilities	0.47	0.31	0.74	1				
Education	0.30	0.12	0.38	0.49	1			
Documents	0.42	0.32	0.45	0.49	0.38	1		
Health	0.28	0.11	0.28	0.50	0.43	0.42	1	
Elections	0.21	0.23	0.36	0.21	0.18	0.21	0.16	1

Our imputation process is based on chained regression^37–39^. In this method, the missing values for each dimension at the individual level are imputed using chained prediction models based on all other dimensions. A chained prediction model starts off by filling all missing values of each dimension with their mean, i.e. an initial guess of the missing value. Then, in the first cycle of the first iteration, the filled in mean values of the most observed dimension, grand corruption in the authorities, are set back to missing and then predicted using linear regression models that include all other available dimensions. Subsequently, the second cycle of the first iteration starts with the second most observed dimension: grand corruption of the local governance. Its initial guess values are now set to missing and predicted with all other dimensions (including the imputed values of the previous dimension, grand corruption in the authorities). Each iteration ends once all dimensions have been cycled through, i.e. the chain of regressions is complete. Each additional iteration applies the exact same approach, though no longer using the initial guesses, but now the imputed values from the previous iteration. More iterations ensure that the imputed values eventually stabilize, i.e. convergence is achieved. In our imputation process, we perform ten consecutive iterations, which is the general strategy^40^. Table 4 summarizes the mean, standard deviation, number of respondents, and the percentage coverage at the survey level for each original dimension. By imputing all dimensions in this manner, we ensure a level playing field for all areas in all years. Without such imputation, the SCI would be sensitive to the bias of unobserved dimensions.

**Table 4:** Mean, standard deviation, number of respondents and the percentage coverage at the survey level for the 11 grand corruption and 8 petty corruption dimensions.

Dimension	Mean	St. Dev.	N	Survey N (%)	Dimension	Mean	St. Dev	N	Survey N (%)
Grand: Executive	0.547	0.307	204,748	24	Grand: Local Governance	0.521	0.281	526,394	53
Grand: Legislative	0.514	0.292	224,252	26	Petty: Authorities	0.879	0.322	729,953	79
Grand: State & Institutions	0.311	0.309	306,662	40	Petty: Government Officials	0.922	0.223	441,262	57
Grand: Judicial	0.577	0.291	354,634	42	Petty: Judicial	0.971	0.166	154,105	45
Grand: Taxation	0.553	0.291	225,587	27	Petty: Utilities	0.912	0.230	59,793	18
Grand: Civil	0.510	0.276	114,450	13	Petty: Education	0.953	0.202	576,358	76
Grand: Politicians	0.378	0.283	68,484	7	Petty: Documents	0.796	0.403	152,030	49
Grand: Government Officials	0.493	0.288	503,156	53	Petty: Health	0.928	0.247	624,640	76
Grand: Severity	0.157	0.257	272,142	16	Petty: Elections	0.525	0.363	339,491	33
Grand: Authorities	0.590	0.282	609,524	49					

### Index construction

To create the two indices of petty and grand corruption, two separate Principal Component Analyses (PCA) were performed, one for each index, using eleven dimensions for grand corruption and eight dimensions for petty corruption. PCA is a multivariate statistical technique aiming to decrease the number of variables in a dataset by converting them into a smaller number of components, whereby each component is a linear weighted combination of the initial variables, which are assumed to be of interval level^41,42^. Although the Likert type variables used in most of our surveys are not strictly of interval level, PCA and Factor Analyses generally perform well with these kinds of variables^32–34^.

We extracted the first component for both PCAs. For grand corruption, this component has an Eigenvalue of 5.277 and captures 48% of the variation. For petty corruption, it has an Eigenvalue of 3.173 and captures 40% of the variation. These variation shares of 48% and 40% are relatively high compared to other fields, like the construction of wealth indices, where the first component tends to explain no more than 30% of the variation (e.g. 26%^43^; 27%^44^; 30%^45^). The Cronbach’s alpha values for internal consistency of the grand and petty corruption indices are with 0.88 and 0.74 clearly above the critical value of 0.70^46^.

The arrows in Fig. 1 capture the relative importance of each dimension in calculating the grand and petty corruption indices by presenting the relative weight of each dimension. For grand corruption all weights are in the order of 10%, except for the severity dimension which is with a score of 3.9 clearly the least important. The weights for petty corruption show somewhat more variation, between 9 and 15 percent, except for bribery related to elections which is with a score of 7% the least important.

After the PCAs, one arrives at 6,494 area observations for grand corruption (97% of sample), and 5,345 observations for petty corruption (80% of sample). Together these observations form the first raw version of our corruption database.

### Handling outliers

While almost all country-surveys have a reasonable sample size (see Supplementary Table 3), the sample sizes are substantially lower once we split up the countries into subnational regions. This means that the influence of fluctuations in the data becomes larger and that the importance of handling outliers increases. Outliers are usually defined as observations that appear to deviate markedly from the other members of the sample in which they occur^47,48^. To address them, we first have to identify them and then apply measures to reduce their influence. Identifying outliers means formulating rules with which they can be distinguished from valid cases^49,50^. Here, the main concern for outliers is regarding the existence of extreme differences at the sub-national level, either compared to other subnations within the same country, or compared to the same subnation over time.

**Fig. 2:**
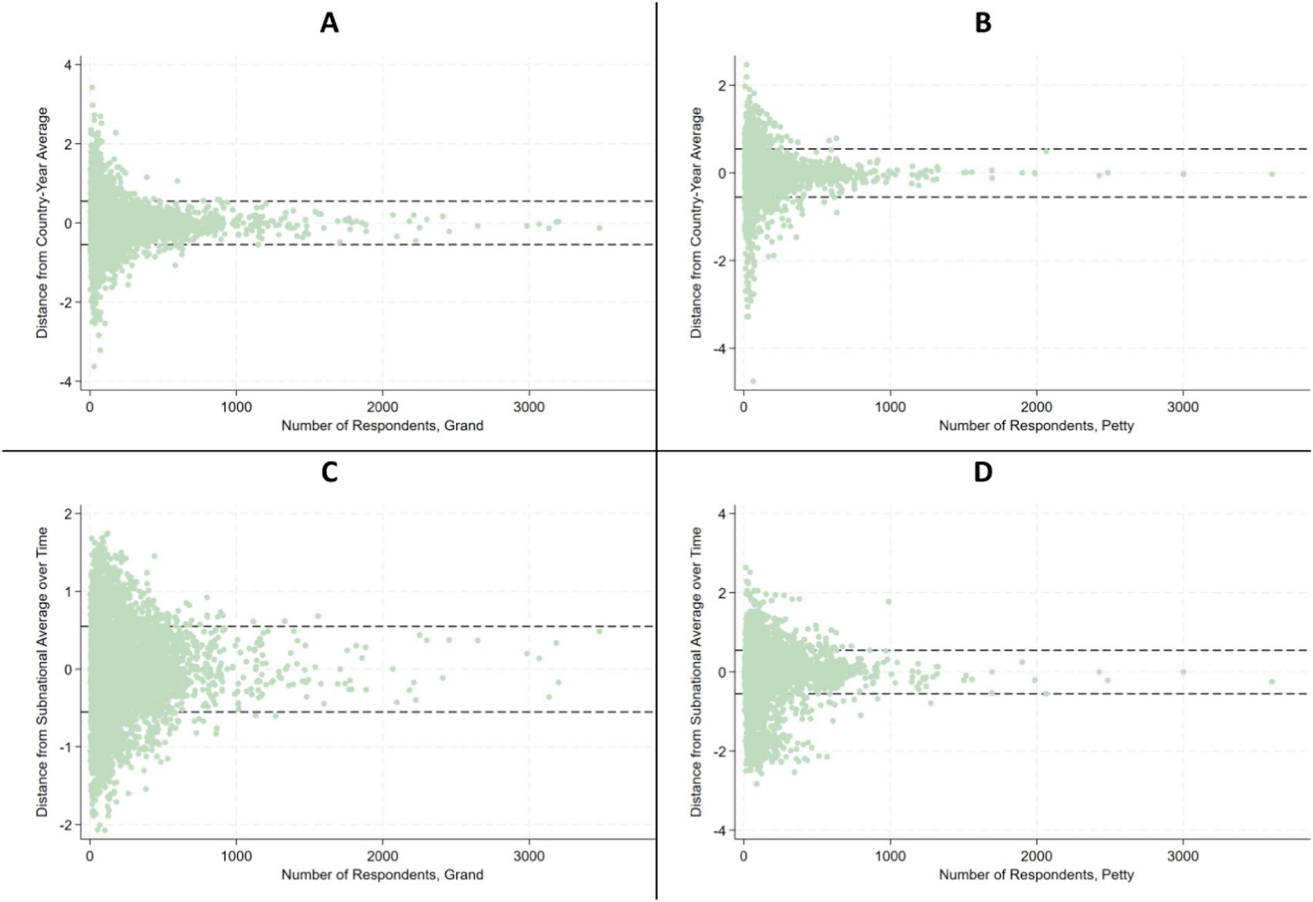
Relationship between residuals and number of underlying respondents for the SGCI (panels A,C) and the SPCI (panels B,D). The residuals reflect the distance of an area-year to the country-year average (panels A,B) and the subnational average over the entire sample (panels C,D).

To address these outliers we use a two-step approach. In the first step, we ran regression analyses with country fixed effects (FE), time FE and country-time FE without independent variables at the subnational level for the petty and grand indices separately. The residuals at this stage reflect the distance between a particular observation and the overall average in the country-year. In Panels A and B of Fig. 2, the size of the residuals and the number of respondents in the raw data source are displayed together. For both indices, a clear nonlinear relationship between the number of respondents and the residual size is observed. This relationship starts to disappear around 1,000 respondents, where the residual tends to lie between −0.55 and 0.55 (dashed lines). We therefore define observations with a residual above 0.55 or below −0.55 as outliers. To address these outliers, trimming or Winsorizing can be used, whereby trimming means removing the outliers completely and Winsorizing reducing their influence^51,52^. As we prefer to keep as many regions as possible in our database, we opt for Winsorizing and choose to limit the maximum movement of each observation to −0.55 and 0.55 of the overall average of the national area in a given year. For instance, if a residual exceeded 0.55, say 0.7, we set the residual to 0.55, effectively dampening extreme variation.

In the second step, we ran regression analyses with only sub-national area FE without independent variables at the subnational level on the adjusted values. These residuals then reflect the distance to the adjusted sub-national area average over time. We continue to find a clear relationship between the number of respondents and the distance from the average in this setup, and again limit these residuals to −0.55 and 0.55 (see Fig. 2, panels C and D).

We also apply an adjustment to areas with only one year of data, for which we can only obtain predicted positive and negative residuals based on the number of respondents and the underlying raw data source. After predicting the residuals, we assess whether our estimate for the subnational area is overestimated (i.e., apply the negative residual) or underestimated (i.e., apply the positive residual) by comparing the relative position of the country to the outcomes of the CCI and the CPI.

As a robustness check, we have developed 50 separate indices where our maximum residual range for both steps ranged from −0.30 to 0.30 and from −0.80 to 0.80, each time increasing the bandwidth by 0.01. All 49 indices correlated about 0.99 with the main index based on the −0.55 and 0.55 range, thus indicating that our criterion for outlier detection has little influence on the performance of the indices.

### Data completion

To obtain values for both grand and petty corruption for the region-year combinations for which only one of these forms is known, each component is estimated using the other in two region-dummy models using OLS regression analysis on 5,128 observations. These specifications are:

$$\widehat{Gran{d}_{i,t}}=-0.043+0.2\,\ast \,Pett{y}_{i,t}+{\zeta }_{i}|Adj.\,{R}^{2}=0.77$$

$$\widehat{Pett{y}_{i,t}}=-1.724+0.148\,\ast \,Gran{d}_{i,t}+{\zeta }_{i}|Adj.{R}^{2}=0.86,$$

where the T-value of the petty and grand coefficients is 10.77 and ${\zeta }_{i}$ denote region fixed effects.

In this way, we can exploit the substantial association between petty and grand corruption (Pearson correlation of 0.40) while also accounting for the fact that we know the values of petty and grand corruption for some regions in other years (region FE). Both linear and nonlinear models were tested, but the linear models turned out to fit better than the nonlinear ones, as the nonlinear terms (squared and cubic) were not significant.

The dataset indicates when a value was predicted (these values are denoted by *SCI2*, *grand2*, and *petty2*) Researchers should use these values only for descriptive purposes and not for explanatory research.

### Subnational Corruption Database (SCD)

After handling the outliers as described in the preceding section, we obtain the ‘*Baseline Dataset*’ of the Subnational Corruption Database (SCD). This *Baseline Dataset* contains for all countries, subnational regions and years for which survey data is available the values of the three central indices, the Subnational Corruption Index (SCI), the Subnational Grand Corruption Index (SGCI) and the Subnational Petty Corruption Index (SPCI). In total, the *Baseline Dataset* contains data for 1,473 regions in 178 countries for selected years in the period 1995–2022.

This *Baseline Dataset* is the first major product of our subnational corruption database. The values of the indices in this dataset can be considered as *absolute* corruption scores, in the sense that they are all computed using the same formulas. This also means that the scores are comparable between the years and can be used to study changes in the three indices over time.

**Fig. 3:**
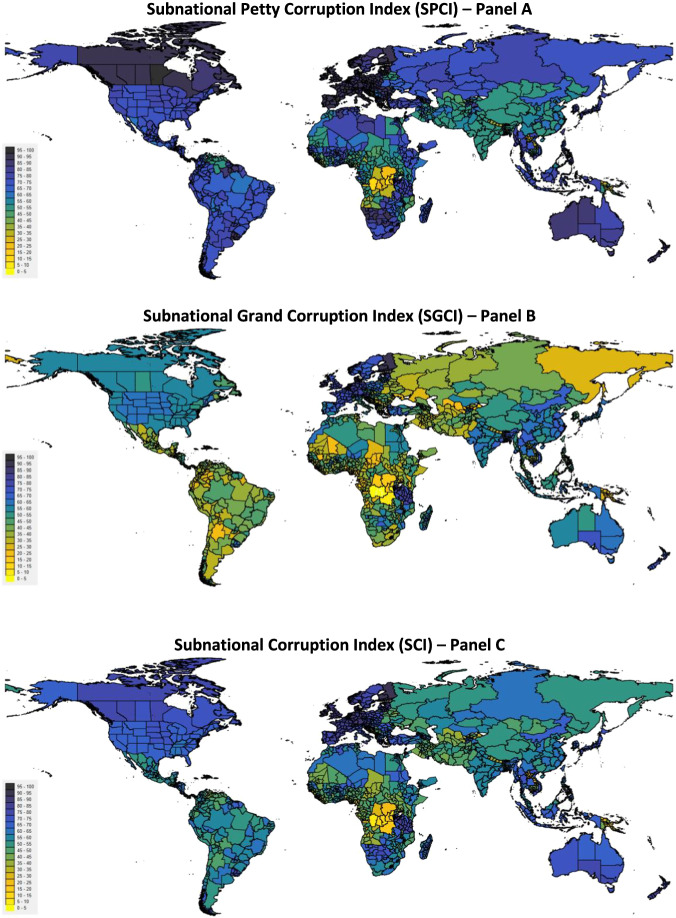
Maps of the SPCI (panel A), SGCI (panel B) and the comprehensive SCI (panel C) showing the most recent available estimate for each area.

The maps in Fig. 3 graphically present the SPCI, SGCI and SCI in panels A, B, and C, respectively. All maps clearly show that there is significant subnational variation in corruption, and that this variation differs substantially when comparing the SPCI and SGCI. Interestingly, the perceptions on grand corruption vary much more than the experiences with petty corruption. We also observe that subnational variation in corruption matters more in more corrupt countries, indicating that our indices are especially relevant for countries that were not accounted for in existing subnational corruption indices.

### Inter- and extrapolation

Given that the Baseline Dataset only contains information for country-year combinations for which survey data were available, the availability of data varies between countries and over time. To achieve improved data coverage for descriptive purposes, two additional steps were taken. First, for the countries for which data for several points in time was available, linear interpolation was used to fill in empty years between the years for which data was available. As no other information for the missing countries is available, presuming that in each of the in-between years the corruption level changed in a similar way does seem a reasonable assumption, which is also applied in other data projects^22^. With this step, the number of area-years for which data was available increased from 7,456 to 17,166.

Second, with this interpolated dataset as a starting point, we extrapolated the data down to 1995 and up to 2022 using time-variation of the World Bank’s CCI. In case of a missing SCI, the CCI is the best and most widely available alternative index to ‘guess’ the direction of the SCI. To extrapolate, we first convert the CCI values into SCI values using OLS regression to ensure the scales are the same:

$$SC{I}_{i}={\beta }_{0}+{\beta }_{1}CC{I}_{i}+{\beta }_{2}CC{I}_{i}^{2}|Adj.{R}^{2}=0.60$$

Subsequently, we applied the absolute change in the CCI (after conversion to the SCI) to the SCI values over time, such that we arrive at a full dataset. For instance, if the converted CCI increased by 3 index points for a country between 2004 and 2005, then we will apply a deduction of 3 points to the real SCI in 2005 when extrapolating to arrive at a value for 2004.

**Fig. 4:**
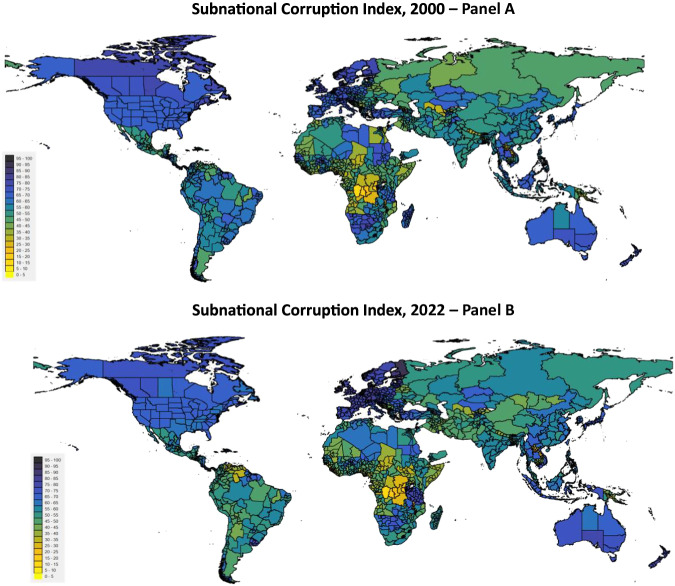
World maps of subnational corruption (using the SCI) in the year 2000 in panel A, and in the year 2022 in panel B.

When extrapolating, we assume no change in the composition of subnational values, as the closest available subnational distribution seems to offer the most likely prediction, given that subnational variation is a sticky phenomenon^53^. Ultimately, this led to a dataset with 45,958 observations on corruption. Given the estimated nature of part of this *Comprehensive Dataset*, researchers should only use it for descriptive purposes and use the *Baseline Dataset* for in-depth analyses. Figure 4 presents the resulting SCI for 2000 and 2022 at world scale.

### Subnational estimates of CPI and CCI

We also offer subnational corruption values that are more easily understandable to a broader audience of policy makers, NGO’s, journalists, and advocacy groups fighting corruption. Given that these groups generally are more familiar with the CPI of Transparency International and the CCI of the World Bank, we have connected the subnational values derived from our datasets with the national values of CPI and CCI. To do this, we have followed an approach used by other scholars^22,54^, who created subnational versions of the UNDP’s Human Development Index (HDI) by standardizing subnational variation derived from household surveys around the national HDI values derived from the Human Development Report database of the UNDP (https://hdr.undp.org/data-center).

The subnational CCI and subnational CPI estimates obtained in this way are called SUBCCI and SUBCPI respectively. The SUBCCI is available across the entire 1995–2022 time period. The SUBCPI runs from 2012 to 2022, as the CPI is only comparable over time from 2012 onwards^20^. Both the SUBCCI and the SUBCPI are equivalent to the official versions of the CCI and CPI at the country level, but add estimates of these indices for subnational regions within the countries. These estimates are scaled in the same way as the official national values.

## Data Records

The Subnational Corruption Database (SCD) provides the levels of total, grand, and petty corruption for 1,473 subnational regions within 178 countries in the period 1995–2022. The SCD also offers subnational estimates of two existing corruption indices, the Control of Corruption Index (CCI) of the World Bank and the Corruption Perceptions Index (CPI) of Transparency International. All the data records are publicly available as a ZIP Archive from the Figshare repository^55^.

The SCD ZIP Archive contains three different data files: “Baseline Dataset SCI”, “Comprehensive Dataset SCI”, and “SUBCPI and SUBCCI Dataset”. Each of these files is available in three different versions; for SPSS, Stata and MS Excel. The SCD also contains two Stata syntax files: “From Raw Dataset to Baseline Dataset.do” and “From Baseline Dataset to Comprehensive Datasets.do”. Below, we also indicate all variable names in closed brackets.

The *Baseline Dataset SCI file* contains the subnational and national values of the Subnational Corruption Index [SCI] and its two components: the Subnational Petty Corruption Index (SPCI) [petty] and the Subnational Grand Corruption Index (SGCI) [grand]. All indices range from 0–100, where 0 is the largest level of corruption and 100 is the lowest level of corruption. These indices are most suitable for explanatory analyses.

The *Baseline Dataset SCI file* also contains predicted values for the SCI [SCI2], SPCI [petty2] and SGCI [grand2], which fill up missing values in years where either the SPCI or the SGCI is missing, by exploiting the relationship between the SPCI and the SGCI. The Baseline Dataset SCI contains 6,701 area-years of corruption data, based on 807 standardized and harmonized household surveys.

The *Comprehensive Dataset SCI file* contains the yearly subnational and national values of the estimated SCI for the period 1995–2022, after applying inter- and extrapolation. This dataset is most suitable for descriptive analyses. The number of subnational regions varies between countries. It can reach up to 37 regions (for Nigeria) with an average of 8.3 regions. The years for which the SCI is available are similar to those of the World Bank’s Control of Corruption (CCI) (from 1995–2022) The total number of country-years for which SCI data is available is 4,930.

The *SUBCPI and SUBCCI Datasets* contains subnational and national estimates of total corruption obtained by superimposing the subnational variation of our SCI around two established measures of corruption: Transparency International’s Corruption Perceptions Index (CPI) and the World Bank Control of Corruption Index (CCI). The [SUBCPI] is available for the period 2012–2022 and the [SUBCCI] for the whole period 1995–2022.

The *From Raw Dataset to Baseline Dataset* Stata do-file shows the syntax steps made to get from the Raw Dataset to our Baseline Dataset SCI. The Raw Dataset contains the underlying questions of all sources at the individual level, after combining all subnational region definitions under one coding scheme. The Raw Dataset is not available given copyright. The *From Baseline Dataset to Comprehensive Datasets* Stata do-file shows the syntax required to move from our Baseline Dataset SCI to our Comprehensive Dataset SCI and SUBCPI and SUBCCI Dataset.

The most recent version of the SCD is available at www.corruptionradar.org. One can find an adjusted version of the SCD integrated with a sub-national socioeconomic database on www.globaldatalab.org.

## Technical Validation

### Correlations with existing indices

To test the performance of the SCI as a corruption index, a first way to gain insight into its effectiveness is to compute correlations with other – more established – corruption indices. Given that the SCI is currently the only available time-varying sub-national corruption index, such a comparison is only possible at the national level. We therefore have computed Pearson correlations of the SCI with the Control of Corruption Index (CCI) of the World Bank’s Worldwide Governance Indicators and with the Corruption Perceptions Index (CPI) of Transparency International. For this analysis, the baseline dataset without estimated versions of the SCI is used, i.e. no prediction, interpolation or extrapolation. The SCI correlates 0.78 with the CCI in the 1995–2022 period as well as in the 2012–2022 period and 0.76 with the CPI in the 2012–2022 period, which is the period over which the CPI is comparable^20^. The high correlations of our SCI with both indices make clear that the SCI at the national level to a large extent overlaps with them. At the same time, the correlation is not perfect. This is unsurprising as the CPI is completely and the CCI largely based on the judgments of experts and business executives, while the SCI reflects the personal experiences and perceptions of corruption of each country’s population.

**Fig. 5:**
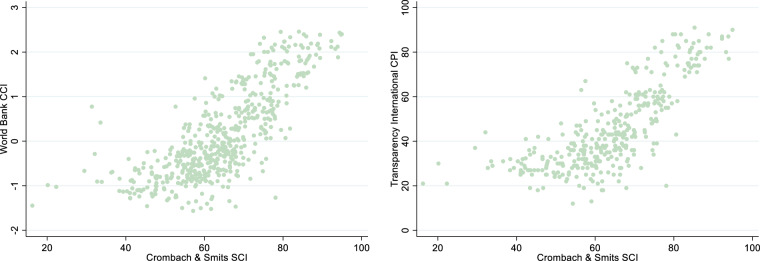
Associations of the national values of the SCI (horizontal axis) with the CCI (vertical axis in left figure) and the CPI (right vertical axis) for the period 1995–2022 (CCI) and 2012–2022 (CPI).

In Fig. 5 the associations of SCI with CCI and CPI are depicted graphically. These figures confirm the high correlations between the SCI and these indices. At the same time, they reveal the existence of some nonlinearity in the associations, with seemingly stronger associations at the higher levels of the indices than at the lower levels. It appears that the SCI is better able to separate the degree of corruption in more corrupt areas. To explore this nonlinearity further, two regression analyses were performed with the CCI and CPI as dependent variables and both the SCI and SCI^2^ as independent variables. In both analyses, significant positive regression coefficients were obtained for both SCI and SCI^2^, thus confirming the existence of nonlinearity in the associations. The nonlinear correlation coefficients computed on the basis of the model’s R2 are 0.81 and 0.82 respectively, thus revealing even higher correlations of SCI with CCI and CPI when nonlinearity is taken into account.

Given that the SCI at the subnational level is constructed in the same way as at the national level, it seems likely that if we were able to compare our index with subnational versions of CCI and CPI, we would find similar high correlations with those indices. This strengthens our trust in the reliability of the SCI as an indicator of the within-country variation in corruption levels.

In terms of petty and grand corruption, we find that the SPCI correlates 0.60 with the CCI overall and 0.60 with the CPI after 2012. Further, we find that the SGCI correlates 0.67 with the CCI overall and 0.70 with the CPI after 2012. As such, the CPI and CCI are somewhat more focussed on grand corruption overall. The finding that the correlation of the CPI and CCI with the overall SCI is higher than the correlations of CPI and CCI with the Grand and Petty subindices is well in line with the fact that CPI and CCI are overall corruption indices.

### Stability of the PCA results

Our subindex for grand corruption was constructed by performing PCA on eleven individual-level variables representing the eleven dimensions we distinguish for grand corruption. In the same way, our index for petty corruption was constructed with a PCA on eight individual-level variables, representing the eight dimensions we distinguish for petty corruption. The PCA weights of these analyses (presented in Table 1) showed that with only a few exceptions (severity of corruption and bribery related to elections) each separate dimension contributed substantially to the PCA outcomes. This raises the question to what extent the final indices for grand and petty corruption are dependent on specific dimensions.

To test for the presence of such dependencies we have computed a number of reduced versions of both indices, each time with one of the dimension indicators removed. For this purpose, 11 new PCAs were conducted for the SGCI and 8 new PCAs for the SPCI. The reduced SGCI and SPCI computed in this way were subsequently correlated with the original indices based on all dimensions. The Pearson correlation coefficients obtained in this way are presented in column “Correlation Upper Bound” of Table 5. All these correlations are in the order of 0.99, hence there are hardly any differences between the original and reduced versions of the indices.

**Table 5:** Correlation coefficients with the grand corruption index (column 2, upper bound; column 3, lower bound) and with the petty corruption index (column 5, upper bound; column 6, lower bound) of the reduced grand and petty corruption indices with one of the dimensions removed. Upper bound columns refer to validation analyses post-imputation, lower bound columns refer to validation analyses pre-imputation.

Grand without	Correlation Upper Bound	Correlation Lower Bound	Petty without	Correlation Upper Bound	Correlation Lower Bound
Executive	0.993	0.975	Authorities	0.987	0.814
Legislative	0.993	0.974	Judicial	0.999	0.985
State & Institutions	0.991	0.974	Utilities	0.999	0.987
Judicial	0.990	0.970	Education	0.995	0.985
Taxation	0.991	0.973	Documents	0.999	0.987
Civil	0.991	0.967	Health	0.995	0.974
Politicians	0.994	0.973	Elections	0.999	0.975
Government Officials	0.990	0.960	Government Officials	0.994	0.941
Severity	0.997	0.978			
Authorities	0.992	0.960			
Local Governance	0.992	0.953			

These high correlations seem convincing, but it is possible that they are inflated by the fact that in the preceding steps of data preparation estimation procedures have been used in which information of one dimension might have spread to other dimensions. We have therefore estimated a second set of reduced indices on the *Baseline Dataset*, where we each time run the imputation process *without* the removed dimension. The correlations of these indices with the indices based on all dimensions are presented in column “Correlation Lower Bound” of Table 5.

These correlations are a little lower than the first set, yet remain impressive. All SGCI dimensions are correlated 0.953 or higher, while almost all SPCI dimensions are correlated 0.941 or higher. The exception is the SPCI authorities dimension, which lower bound correlates 0.814, highlighting the somewhat more important role of petty corruption in the authorities in the imputation process.

Overall, we can conclude that there are hardly any differences between the original and reduced versions of the indices, which indicates that they are highly robust against removal of a dimension and that none of the separate dimensions is thus of critical importance for their construction. The findings of this robustness test are in line with earlier findings in the field of wealth measurement and climate vulnerability, where PCA-based composite indices also turned out to be highly robust against the removal of one or two of the underlying variables^45,56^.

### PCA versus factor analysis

Another way to test the robustness of the PCA results is to compare them with the outcomes of Factor Analysis as an alternative method for data reduction and index construction. We therefore have constructed alternative versions of the Grand and Petty corruption indices by using Factor Analysis (FA) on the same datasets that were used as input for the PCA. To compare the outcomes of both types of analyses, Pearson correlations were computed between the PCA-based and FA-based indices. These correlations turned out to be larger than 0.99 for both the grand and petty indices, thus highlighting that both methods lead to almost exactly the same index.

### Supplementary information

Supplementary Tables

## Data Availability

All original survey datasets used for constructing the SCD are freely available from the data collecting organizations. The websites of these organizations are mentioned in Supplementary Table 1. The database was constructed from these survey datasets with Stata-16. The Stata Do files with the syntax code used for processing the data and constructing the index are included in the ZIP Archive with the Subnational Corruption Database available from the Figshare repository^55^.
