# Supplementary material for: The Subnational Corruption Database: Grand and petty corruption in 1,473 regions of 178 countries, 1995–2022
Source: Sci Data. 2024 Jun 25;11:686. doi: 10.1038/s41597-024-03505-8 (PMC11199513; doi:10.1038/s41597-024-03505-8)
Supplement: Supplementary file 1 — Supplementary Tables [file 41597_2024_3505_MOESM1_ESM.pdf]

Supplementary Table 1 - Data sources, countries, years and dimensions covered.

Data Source	Link/Citation	Countries	Years	Dimensions
Afrobarometers	https://www.afrobarometer.org[24]	Algeria; Angola; Benin; Botswana; Burkina Faso; Burundi; Cameroon; Cape Verde; Côte d'Ivoire; Egypt; Eswatini; Ethiopia; Gabon; Gambia; Ghana; Guinea; Kenya; Lesotho; Liberia; Madagascar; Malawi; Mali; Mauritius; Morocco; Mozambique; Namibia; Niger; Nigeria; Senegal; Sierra Leone; South Africa; Sudan; São Tomé and Príncipe; Tanzania; Togo; Tunisia; Uganda; Zambia; Zimbabwe	2008, 2012, 2014-2021	Executive [Grand]; Legislative [Grand]; Judicial [Grand]; Taxation [Grand]; Civil [Grand]; Government Officials [Grand]; Authorities [Grand]; Local Government [Grand]; Authorities [Petty]; Utilities [Petty]; Education [Petty]; Documents [Petty]; Health [Petty]; Election [Petty]
Arabbarometers	https://www.arabbarometer.org/survey-data/	Algeria; Egypt; Iraq; Jordan; Kuwait; Lebanon; Libya; Morocco; Palestine; Tunisia; Yemen	2011, 2014, 2017, 2021	State [Grand]
Asian Barometers	https://www.asianbarometer.org/datar?page=d10	Cambodia; China; India; Indonesia; Japan; Malaysia; Mongolia; Singapore; South Korea; Thailand; Vietnam	2010, 2014-2016, 2018, 2019	State [Grand]; Local Government [Grand]
Eurobarometers	https://europa.eu/eurobarometer/surveys/browse/all	Austria; Belgium; Bulgaria; Croatia; Cyprus; Czechia; Denmark; Estonia; Finland; France; Germany; Greece; Hungary; Ireland; Italy; Latvia; Lithuania; Luxembourg; Netherlands; Poland; Portugal; Romania; Slovakia; Slovenia; Spain; Sweden; United Kingdom	2007, 2011, 2017, 2019, 2022	State [Grand]; Judicial [Grand]; Government Official [Grand]; Authorities [Grand]; Local Government [Grand]; Authorities [Petty]; Government Officials [Petty]; Judicial [Petty]; Education [Petty]; Health [Petty]
European Quality of Government Database	https://www.gu.se/en/quality-government/qog-data[25,15,26,27,28]	Austria; Belgium; Bulgaria; Czechia; Denmark; Estonia; Finland; France; Germany; Greece; Hungary; Ireland; Italy; Latvia; Lithuania; Malta; Netherlands; Poland; Portugal; Romania; Slovakia; Slovenia; Spain; Sweden	2010, 2013, 2017, 2021	Authorities [Grand]; Authorities [Petty]; Education [Petty]; Health [Petty]; Election [Petty]
International Social Survey Programme	https://www.gesis.org/en/issp/modules/issp-modules-by-topic/role-of-government/2006	Argentina; Australia; Canada; Chile; China; Czechia; Denmark; Dominican Republic; Finland; France; Germany; Hungary; Ireland; Israel; Japan; Latvia; Netherlands; New Zealand; Norway; Poland; Portugal; Russian Federation; Slovakia; Slovenia; South Africa; South Korea; Spain; Sweden; Switzerland; United States	2006	Politician [Grand]; Government Officials [Grand]; Government Officials [Petty]
Vanderbilt AmericasBarometer (LAPOP)	www.vanderbilt.edu/lapop	Argentina; Bahamas; Barbados; Belize; Bolivia; Brazil; Canada; Colombia; Costa Rica; Dominican Republic; Ecuador; El Salvador; Guatemala; Guyana; Haiti; Honduras; Jamaica; Mexico; Nicaragua; Panama; Paraguay; Peru; Suriname; United States; Uruguay	2004, 2006-2010, 2012, 2014, 2016-2019	Politician [Grand]; Government Officials [Grand]; Authorities [Petty]; Government Officials [Petty]; Judicial [Petty]; Education [Petty]; Documents [Petty]; Health [Petty]
Latinobarómetros	https://www.latinobarometro.org/latContents.jsp	Argentina; Bolivia; Brazil; Chile; Colombia; Costa Rica; Dominican Republic; Ecuador; El Salvador; Guatemala; Honduras; Mexico; Nicaragua; Panama; Paraguay; Peru; Uruguay; Venezuela	1998, 2000, 2001, 2013, 2016-2018	Executive [Grand]; Legislative [Grand]; State [Grand]; Judicial [Grand]; Taxation [Grand]; Civil [Grand]; Government Officials [Grand]; Severity [Grand]; Authorities [Grand]; Local Government [Grand]

The Asia Foundation: Afghanistan<br><br>And<br><br>The Asia Foundation: Timor-Leste	https://asiafoundation.org/	Afghanistan; Timor-Leste	2006-2019 (Afghanistan); 2014, 2016, 2018 (Timor-Leste)	Executive [Grand]; Legislative [Grand]; State [Grand]; Judicial [Grand]; Politician [Grand]; Severity [Grand]; Authorities [Grand]; Local Government [Grand] Severity [Grand];
Global Corruption Barometers, 9th edition (Note: Only national data)<br><br>Global Corruption Barometer for Africa, 10th edition and Global Corruption Barometer Pacific, 1st edition	https://www.transparency.org/en/gcb/global/global-corruption-barometer-2017/press-and-downloads<br><br>https://www.transparency.org/en/gcb/africa/africa-2019/press-and-downloads<br><br>https://www.transparency.org/en/gcb/pacific/pacific-2021	National GCB:<br>Austria; Belgium; Bulgaria; Croatia; Cyprus; Czechia; Denmark; Estonia; Finland; France; Germany; Greece; Hungary; Ireland; Italy; Latvia; Lithuania; Luxembourg; Netherlands; Poland; Portugal; Romania; Slovakia; Slovenia; Spain; Sweden; United Kingdom<br>Subnational GCB:<br>The Democratic Republic of Congo; Cook Islands; Micronesia; Fiji; French Polynesia; Kiribati; Marshall Islands; Nauru; New Caledonia; Niue; Papua New Guinea; Palau; Samoa; Solomon Islands; Tokelau; Tonga; Tuvalu; Vanuatu	National: 2017; Sub-national: 2019; 2021	National:<br>Judicial [Grand]; Taxation [Grand]; Government Officials [Grand]; Authorities [Grand];<br>Sub-national:<br>Executive [Grand];<br>Legislative [Grand];<br>State [Grand]; Judicial [Grand]; Civil [Grand]; Government Officials [Grand]; Authorities [Grand]; Local Government [Grand]; Authorities [Petty]; Judicial [Petty]; Utilities [Petty]; Education [Petty]; Documents [Petty]; Health [Petty]; Elections Petty]
World Bank Country Opinion Survey Program	https://www.worldbank.org/en/programs/world-bank-country-opinion-surveys	Bhutan; Central African Republic; Chad; Comoros; Congo Brazzaville; Djibouti; Equatorial Guinea; Guinea Bissau; Maldives; Mauritania; Seychelles; Somalia; South Sudan; Turkmenistan; Uzbekistan	2012-2021	State [Grand]
World Bank Enterprise Surveys	http://www.enterprisesurveys.org	Bosnia and Herzegovina; Kosovo; Lao; Montenegro; Nepal; Sri Lanka	2009, 2011-2013, 2016, 2019	State [Grand]; Taxation [Grand]; Government Officials [Petty]; Utilities [Petty]; Documents [Petty]
World Values Survey	https://www.worldvaluessurvey.org/wvs.jsp[29-31]	Albania; Algeria; Andorra; Argentina; Armenia; Australia; Azerbaijan; Bangladesh; Belarus; Bolivia; Brazil; Bulgaria; Chile; China; Colombia; Cyprus; Dominican Republic; Ecuador; Egypt; El Salvador; Estonia; Ethiopia; Georgia; Germany; Ghana; Greece; Guatemala; Hungary; India; Indonesia; Iran; Iraq; Japan; Jordan; Kazakhstan; Kuwait; Kyrgyzstan; Latvia; Lebanon; Libya; Lithuania; Malaysia; Moldova; Myanmar; Netherlands; New Zealand; Nicaragua; Nigeria; North Macedonia; Pakistan; Palestine; Peru; Philippines; Poland; Puerto Rico; Romania; Russian Federation; Rwanda; Serbia; Slovakia; South Africa; South Korea; Spain; Switzerland; Tajikistan; Thailand; Tunisia; Turkey; Ukraine; United Kingdom; United States; Uruguay; Vietnam; Yemen; Zimbabwe	1995-1999, 2011-2014, 2017-2020	State [Grand]; Civil [Grand]; Severity [Grand]; Local Government [Grand]; Government Officials [Petty]; Election [Petty]

Supplementary Table 2 – Overview of all survey questions and their adjustments.

Question	Original Units	Source	Year (Question)	Adjustments
**Grand: Executive**				
**How many of the following people do you think are involved in corruption, or haven't you heard enough about them to say: The President/Prime Minister and Officials in his Office?**	None<br>Some of them<br>Most of them<br>All of them	Afrobarometers Transparency International Democratic Republic of Congo	2008-2011 (Q50A), 2012-2013 (Q60A), 2014-2015 (Q53A), 2017-2019 (Q44A), 2019-2022 (Q42A)	
**How many of the following people do you think are involved in corruption, or haven't you heard enough about them to say?: The president and officials in his/her office**	None<br>Some<br>Most<br>All	Latinobarometros	2016 (P1TI.A), 2018 (Q71TI.A)	
**How many of the following people do you think are involved in corruption? The President and Officials in his/her Office | The Prime Minister and Officials in his/her Office (combined)**	None<br>Some of them<br>Most of them<br>All of them	Transparency International	2017 (TI_Q6_1 and TI_Q6_2)	
**To what extent do you perceive the following areas to be affected by corruption? Office of the President.**	Not at all<br>A little<br>Moderately<br>Significantly<br>Extremely	The Asia Foundation Timor-Leste Tatoli Public Perception Survey	2016, 2018 (m47_09 and m47_10)	
**Grand: Legislative**				
**How many of the following people do you think are involved in corruption, or haven't you heard enough about them to say: Members of Parliament**	None<br>Some of them<br>Most of them<br>All of them	Afrobarometers Transparency International Democratic Republic of Congo	2008-2011 (Q50B), 2012-2013 (Q60B), 2014-2015 (Q53B), 2017-2019 (Q44B), 2019-2022 (Q42B)	
**How many of the following people do you think are**	None<br>Some<br>Most	Latinobarometros	2016 (P1TI.B), 2018 (Q71TI.B)	

**involved in corruption, or haven't you heard enough about them to say?: Members of Parliament**	All			
**How much corruption do you think there is in congress?**	0 (None) to 10 (A lot)	Latinobarometros	2017 (Q42NC.E)	Where other questions focus on the number of corrupt parliamentarians, this question asks about the degree of corruption in congress. This led to downward bias (more corruption). To adjust, country-level averages were set to the interpolated value between Latinobarometro2016 and Latinobarometro2018 where possible. Brazil was not available in 2016 or 2018, and consequently received a global adjustment based on the average difference between this question and the other questions for the same group.
**How many of the following people do you think are involved in corruption? The President and Officials in his/her Office | The Prime Minister and Officials in his/her Office (combined)**	None<br>Some of them<br>Most of them<br>All of them	Transparency International	2017 (TI_Q6_3)	
**To what extent do you perceive the following areas to be affected by corruption? Parliament.**	Not at all<br>A little<br>Moderately<br>Significantly<br>Extremely	The Asia Foundation Timor-Leste Tatoli Public Perception Survey	2016, 2018 (m47_03)	
**Grand: State & Institutions**				
**Do you think that there is corruption within the state's institutions and agencies?**	2011, 2014:<br>Yes<br>No<br>2014, 2017, 2021:<br>To a large extent<br>To a medium extent<br>To a small extent	Arabbarometers	2011, 2014, 2017, 2021 (Q210)	Where other questions allow for more refined responses, the 2011 and 2014 versions of this question only allow for a binary response. In this particular case, this led to a downward bias (more corruption). To adjust, we use overlap between the World Values Survey2014 and the

	Not at all			Arabbarometers2014 based on Jordan, Kuwait and Yemen. These overlap countries receive a full adjustment, while all other countries in the Arabbarometers with a binary response question received the average adjustment of the overlap countries.
**Among the following groups of people, how many do you believe are involved in corruption? Tell me for each group if you believe it is none of them, few of them, most of them or all of them? State authorities**	None of them<br>Few of them<br>Most of them<br>All of them	World Values Survey	2017-2020 (Q113)	Where other questions focus on the degree of corruption, this question asks about number of corrupt people. This led to upward bias (less corruption). To adjust, we use overlap between the Latinobarometro2017 and Eurobarometers2017 and 2019 based on Argentina, Bolivia, Greece and Cyprus. These overlap countries receive a full adjustment, while all other countries in the WVS received the average adjustment of the overlap countries.
**How widespread: Corruption is within the government in your country**	1 (None or low corruption) to 10 (high corruption)	World Values Survey	2012-2014 (MN_228N)	
**How much corruption do you think there is in: National Government?**	0 (None) to 10 (A lot)	Latinobarometro	2017 (Q42NC.C)	
**How widespread do you think corruption and bribe-taking are in the national government? Would you say …?**	Hardly anyone is involved<br>Not a lot of officials are corrupt<br>Most officials are corrupt<br>Almost everyone is corrupt	Latinobarometro	2013 (Q65GBS)	Where other questions focus on the degree of corruption, this question asks about number of corrupt people. This led to upward bias (less corruption). To adjust, we apply the WVS adjustment above to all Latinobarometro2013, as there is no overlap between Latinobarometro2013 and other sources with questions concerning the degree of corruption.
**To what extent do you perceive the following institutions to be affected by corruption? Council of Ministers**	Not at all<br>A little<br>Moderately<br>Significantly<br>Extremely	The Asia Foundation Timor-Leste Tatoli Public Perception Survey	2016, 2018 (m47_11)	

**Percent of firms expected to give gifts to secure a government contract**	0 (None) to 100 (All)	World Bank Enterprise Surveys	2007, 2009, 2011-2013, 2016, 2019 (corr2)	This WBES question was adjusted based on a country-comparison between the WBES and the WVES2012-2014 based on overlap between Iraq, Jordan and Tunisia. We only include WBES data if the country is not available in any other source.
**Has corruption been mentioned as an issue or area of improvement at all?**	No<br>Yes	World Bank Group Country Surveys	2012-2021	All WBGCS questions were adjusted based on a country-comparison between the WBGCS and the WVS2012-2014 based on overlap between Tunisia and Jordan. We only include WBGCS data if the country is not available in any other source. If in both WBES and WBGCS, WBES is preferred given larger sample sizes and more subnational variation.
**How widespread do you think corruption and bribe-taking are in the national government?**	Almost everyone is corrupt<br>Most officials are corrupt<br>Not a lot of officials are corrupt<br>Hardly anyone is involved	Asian Barometers	2010 (Malaysia, q117), 2014-2016, 2018-2019 (q118)	Where other questions focus on the degree of corruption, this question asks about number of corrupt people. This led to upward bias (less corruption). To adjust, we use overlap between the adjusted World Values Survey and the Asian Barometers2018 and Asian Barometers2019 based on China, Japan, and Thailand. These overlap countries receive a full adjustment, while all other countries in the Asian Barometers received the average adjustment of the overlap countries.
**How much of a problem, if at all, is corruption in the government in this country? Is it…?**	No problem at all<br>Fairly small<br>Fairly big<br>A very big problem	Transparency International	2017 (Q2a)	
**Please tell whether you agree or disagree with each of the following? There is corruption in the national public institutions in this country**	Totally disagree<br>Tend to disagree<br>Tend to agree<br>Totally agree	Eurobarometers	2007 (QB1.4), 2011 (QC1.4), 2017 (QB15.2), 2019 (QB15.2), 2022 (QA15.2)	
**Grand: Judicial**				

**How many of the following people do you think are involved in corruption, or haven't you heard enough about them to say: Judges and Magistrates**	None<br>Some of them<br>Most of them<br>All of them	Afrobarometers<br>Transparency International<br>Democratic Republic of Congo	2008-2011 (Q50G), 2012-2013 (Q60G), 2014-2015 (Q53G), 2017-2019 (Q44F), 2019-2022 (Q42F)	
**How many of the following people do you think are involved in corruption, or haven't you heard enough about them to say?: Judges and Magistrates.**	None<br>Some<br>Most<br>All	Latinobarometros	2016 (P1TI.G), 2018 (Q71TI.G),	
**How much corruption do you think there is in the courts of justice?**	0 (None) to 10 (A lot)	Latinobarometros	2017 (Q42NC.A)	Where other questions focus on the number of corrupt parliamentarians, this question asks about the degree of corruption in the courts of justice. This led to downward bias (more corruption). To adjust, country-level averages were set to the interpolated value between Latinobarometro2016 and Latinobarometro2018 where possible. Brazil was not available in 2016 or 2018, and consequently received a global adjustment based on the average difference between this question and the other questions for the same group.<br><br>This question concerns the degree of corruption, while others ask about the number of corrupt individuals. This leads to bias, as respondents are more likely to report a lot of corruption than to report that almost all public officials are corrupt. This question was adjusted downard (more corruption) for comparability. The adjustment is based on overlap between Q42NC.A and Q71TI.G in 2017-2018 for Argentina, Bolivia, Chile, Colombia, Costa Rica, Dominican Republic, Ecuador,

				El Salvador, Guatemala, Honduras, Mexico, Nicaragua, Panama, Peru, Peru, Uruguay
**How many of the following people do you think are involved in corruption? Judges and Magistrates**	None<br>Some of them<br>Most of them<br>All of them	Transparency International	2017 (TI_Q6_7)	
**To what extent do you perceive the following areas to be affected by corruption? National court AND Judicial system**	Not at all<br>A little<br>Moderately<br>Significantly<br>Extremely	The Asia Foundation Timor-Leste Tatoli Public Perception Survey	2016, 2018 (m46_03 and m47_07)	
**How many of the following people do you think are involved in corruption, or haven't you heard enough about them to say? Judges and Magistrates**	None<br>Some of them<br>Most of them<br>All of them	Global Corruption Barometers	2017 (TI1G)	
**Do you think that the giving and taking of bribes and the abuse of power for personal gain are widespread among any of the following? The courts (tribunals) (2017-2022)/ The people working in the judicial services**	Mentioned<br>Not mentioned	Eurobarometers	2007 (QB2.3), 2011 (QC4.3), 2017 (QB7.3), 2019 (QB7.3), 2022 (QA7.3)	This question leads to unfair comparisons as respondents must mention the institutions they think are corrupt, which tends to lead to bias towards less corruption. The Eurobarometers have been rescaled at the national level using Transparency International's Global Corruption Barometer in 2017 (# of corrupt people). Time and subnational variation are still taken from the Eurobarometers.
**Grand: Taxation**				
**How many of the following people do you think are involved in corruption, or haven't you heard enough about them to say: Tax Officials (e.g. Ministry of Finance or Local Government tax collectors)**	None<br>Some of them<br>Most of them<br>All of them	Afrobarometers	2008-2011 (Q50F), 2012-2013 (Q60F), 2014-2015 (Q53F), 2019-2022 (Q42G)	
**How many of the following people do you think are**	None<br>Some<br>Most	Latinobarometros	2016 (P1TI.F), 2018 (Q71TI.F)	

**involved in corruption, or haven't you heard enough about them to say?: Tax officials, like ministry Of finance or local government tax collectors**	All			
**Percentage of firms expected to give gifts in meetings with tax officials**	0 (None) to 100 (All)	World Bank Enterprise Surveys	2007, 2009, 2011-2013, 2016, 2019 (corr1)	This WBES question was adjusted based on a country-comparison between the WBES and the Afrobarometers and the Latinobarometros based on overlap between Namibia, Nigeria, Senegal, Tunisia, Dominican Republic, El Salvador, Honduras and Nicaragua. We only include WBES data if the country is not available in any other source.
**How many of the following people do you think are involved in corruption, or haven't you heard enough about them to say? Tax Officials, like Ministry of Finance officials or Local Government tax collectors**	None<br>Some of them<br>Most of them<br>All of them	Global Corruption Barometers	2017 (TI1F)	
**Do you think that the giving and taking of bribes and the abuse of power for personal gain are widespread among any of the following? Tax authorities**	Mentioned<br>Not mentioned	Eurobarometers	2017 (QB7.2), 2019 (QB7.2), 2022 (QA7.2)	This question leads to unfair comparisons as respondents must mention the institutions they think are corrupt, which tends to lead to bias towards less corruption. The Eurobarometers have been rescaled at the national level using Transparency International's Global Corruption Barometer in 2017 (# of corrupt people). Time and subnational variation are still taken from the Eurobarometers.
**Grand: Civil**				
**How many of the following people do you think are involved in corruption, or**	None<br>Some of them<br>Most of them<br>All of them	Afrobarometers	2019-2022 (Q42C)	

**haven't you heard enough about them to say: Civil servants**				
**Imagine the total number of public employees are 100 and you would have to say how many of those you think are corrupted. How many would you say?**	0 (None) to 100 (All)	Latinobarometros	2001 (P29N)	
**Among the following groups of people, how many do you believe are involved in corruption? Tell me for each group if you believe it is none of them, few of them, most of them or all of them? Civil service providers**	None of them<br>Few of them<br>Most of them<br>All of them	World Values Survey	2017-2020 (Q116)	
**How many of the following people do you think are involved in corruption? Public servants or civil servants AND Workers in civil society organisations**	None<br>Some of them<br>Most of them<br>All of them	Transparency International	2017 (Q6_4 and Q6_10)	
**Grand: Politicians**				
**In your opinion, about how many politicians are involved in corruption?**	Almost none<br>A few<br>Some<br>Quite a lot<br>Almost all	International Social Survey Programme	2006 (ZA4700:V61)	
**Amount of corruption among politicians?**	None<br>Less than half of them<br>Half of them<br>More than half of them<br>All	AmericasBarometer LAPOP	2016-2019 (exc7new)	
**To what extent do you perceive the following areas to be affected by corruption? Political parties**	Not at all<br>A little<br>Moderately<br>Significantly<br>Extremely	The Asia Foundation Timor-Leste Tatoli Public Perception Survey	2016, 2018 (m47_01)	
**Grand: Government Officials**				
**How many of the following people do you think are involved in**	None<br>Some of them<br>Most of them<br>All of them	Afrobarometers Transparency International	2008-2011 (Q50D), 2012-2013 (Q60C), 2014-2015	

**corruption, or haven't you heard enough about them to say: Government officials**		Democratic Republic of Congo	(Q53C), 2017-2019 (Q44C)	
**Perception of Public Corruption among Public Officials?**	Very widespread<br>Somewhat widespread<br>Not very widespread<br>Not widespread at all	AmericasBarometer LAPOP	2004, 2006-2010, 2012, 2014, 2016-2019 (exc7)	Where other questions focus on the degree of corruption, this question asks about number of corrupt people. This led to downward bias (more corruption). To adjust, we use overlap between the LAPOP2018, and Latinobarometros2018 based on Colombia, El Salvador and Honduras. These overlap countries receive a full adjustment, while all other countries in the WVS received the average adjustment of the overlap countries.
**How many of the following people do you think are involved in corruption, or haven't you heard enough about them to say?: Government officials**	None<br>Some<br>Most<br>All	Latinobarometros	2016 (P3TI.C), 2018 (Q73TI.C)	
**In your opinion, about how many public officials are involved in corruption?**	Almost none<br>A few<br>Some<br>Quite a lot<br>Almost all	International Social Survey Programme	2006 (ZA4700:V61)	
**How many of the following people do you think are involved in corruption, or haven't you heard enough about them to say? Government Officials**	None<br>Some of them<br>Most of them<br>All of them	Global Corruption Barometers	2017 (TI1C)	
**Do you think that the giving and taking of bribes and the abuse of power for personal gain are widespread among any of the following? Officials awarding public tenders + issuing building permits + issuing**	Mentioned<br>Not mentioned	Eurobarometers	2007 (QB2.7.8.9.12), 2011 (QC4.7.8.9.12), 2017 (QB7.8.9.10.13), 2019 (QB7.8.9.10.13), 2022 (QA7.8.9.10.13)	This question leads to unfair comparisons as respondents must mention the institutions they think are corrupt, which tends to lead to bias towards less corruption. The Eurobarometers have been rescaled at the national level using Transparency International's Global Corruption Barometer in 2017 (# of corrupt people). Time

**business permits + inspectors**				and subnational variation are still taken from the Eurobarometers.
**Grand: Severity**				
**How widespread do you think bribe taking and corruption is in this country?**	Almost no public officials are engaged in it<br>A few public officials are engaged in it<br>Most public officials are engaged in it<br>Almost all public officials are engaged in it	World Values Survey	1995-1999 (V213)	Where other questions focus on the degree of corruption, this question asks about number of corrupt people. This led to upward bias (less corruption). To adjust, we use overlap between the Latinobarometros1998, and the WVS based on El Salvador and Peru. These overlap countries receive a full adjustment, while all other countries in the WVS received the average adjustment of the overlap countries.
**How would you place your views on corruption in your country?**	1 There is no corruption in my country<br>10 There is abundant corruption in my country	World Values Survey	2017-2020 (q112)	
**Thinking about the problem of corruption in [country] today, would you say that the problem is**	Very serious<br>Serious<br>Not very serious<br>Not at all serious	Latinobarometros	1998, 2000-2001 (P17ST)	
**How prevalent has corruption been in the past 6 months?**	Very high<br>High<br>Low<br>Very low<br>None	The Asia Foundation Timor-Leste Tatoli Public Perception Survey	2014 (Q17_h)	
**Please tell me whether you think that corruption is a major problem, a minor problem, or no problem at all in Afghanistan as a whole**	Major problem<br>Minor problem<br>Not a problem	The Asia Foundation: Survey of the Afghan People	2006-2019 (x23e)	
**Grand: Authorities**				
**How many of the following people do you think are involved in corruption, or haven't you heard enough about them to say: Police**	None<br>Some of them<br>Most of them<br>All of them	Afrobarometers<br>Transparency International<br>Democratic Republic of Congo	2008-2011 (Q50E), 2012-2013 (Q60E), 2014-2015 (Q53E), 2017-2019 (Q44E), 2019-2022 (42E)	

**How many of the following people do you think are involved in corruption, or haven't you heard enough about them to say?: Police**	None<br>Some<br>Most<br>All	Latinobarometros	2016, 2018 (Q75TI.E)	
**To what extent do you perceive the following areas or institutions in this country to be affected by corruption? Police (PNTL)**	Not at all<br>A little<br>Moderately<br>Significantly<br>Extremely	The Asia Foundation Timor-Leste Tatoli Public Perception Survey	2016, 2018 (M47_05)	
**How many of the following people do you think are involved in corruption – would it be none of them, some of them, most of them, or all of them? Police.**	None<br>Some of them<br>Most of them<br>All of them	Transparency International	2017 (Q6_6)	
**Corruption is prevalent in the police force in my area.**	1 Strongly disagree<br>10 Strongly agree	European Quality of Government Index	2010, 2013, 2017 (Q15), 2021 (Q16)	
**How many of the following people do you think are involved in corruption, or haven't you heard enough about them to say? Police**	None<br>Some of them<br>Most of them<br>All of them	Global Corruption Barometers	2017 (TI1E)	
**Do you think that the giving and taking of bribes and the abuse of power for personal gain are widespread among any of the following? Police, customs**	Mentioned<br>Not mentioned	Eurobarometers	2007 (QB2.1.2), 2011 (QC4.1.2), 2017 (QB7.1), 2019 (QB7.1), 2022 (QA7.1)	This question leads to unfair comparisons as respondents must mention the institutions they think are corrupt, which tends to lead to bias towards less corruption. The Eurobarometers have been rescaled at the national level using Transparency International's Global Corruption Barometer in 2017. Time and subnational variation are still taken from the Eurobarometers.
**Grand: Local Governance**				
**How many of the following people do**	None<br>Some of them	Afrobarometers	2008-2011 (Q50C), 2012-	

**you think are involved in corruption, or haven't you heard enough about them to say: local government councilors**	Most of them<br>All of them	Transparency International Democratic Republic of Congo	2013 (Q60D), 2014-2015 (Q53D), 2017-2019 (Q44D), 2019-2022 (Q42D)	
**How many of the following people do you think are involved in corruption, or haven't you heard enough about them to say? Local government councilors.**	None<br>Some<br>Most<br>All	Latinobarometros	2016, 2018 (Q74TI.D)	
**How much corruption do you think there is in county councils?**	0 None<br>10 A lot	Latinobarometros	2017 (Q42NC.D)	This question concerns the degree of corruption, while others ask about the number of corrupt individuals within a group. This leads to bias, as respondents are more likely to report a lot of corruption than to report that all councillors are corrupt. This question was adjusted upward (less corruption) for comparability. The adjustment is based on overlap between q115 and Q42NC.D in 2016-2018 for Argentina, Bolivia.
**How widespread do you think corruption and bribe-taking are in your local/municipal government?**	Hardly anyone is involved<br>Not a lot of officials are corrupt<br>Most officials are corrupt<br>Almost everyone is corrupt	Latinobarometros	2013 (Q64GBSM)	
**To what extent do you perceive the following institutions in this country to be affected by corruption? Municipal government AND Suku council**	Not at all<br>A little<br>Moderately<br>Significantly<br>Extremely	The Asia Foundation Timor-Leste Tatoli Public Perception Survey	2016, 2018 (m47_04 and m47_18)	This question concerns the degree of corruption, while others ask about the number of corrupt individuals within a group. This leads to bias, as respondents are more likely to report extreme corruption than to report that all councillors are corrupt. This question was adjusted upward (less corruption) for comparability. The

				adjustment is based on overlap between q115 and Q42NC.D in 2016-2018 for Argentina, Bolivia.
**How many of the following people do you think are involved in corruption? Local government councillors**	None<br>Some of them<br>Most of them<br>All of them	Transparency International	2017 (Q6_5)	
**Please tell me whether you think that corruption is a major problem, a minor problem, or no problem at all in the following areas… In your local authorities**	Major problem<br>Minor problem<br>No problem at all	The Asia Foundation: Survey of the Afghan People	2007-2016 (x23c)	This question concerns the degree of corruption, while others ask about the number of corrupt individuals within a group. This leads to bias, as respondents are more likely to report corruption as a major problem than to report that all councillors are corrupt. This question was adjusted upward (less corruption) for comparability. The adjustment is based on overlap between q115 and Q42NC.D in 2016-2018 for Argentina, Bolivia, Chile, Colombia and Peru.
**How widespread do you think corruption and bribe-taking are in your local/municipality?**	Almost everyone is corrupt<br>Most officials are corrupt<br>Not a lot of officials are corrupt<br>Hardly anyone is involved	Asian Barometers	2010 (q116), 2014-2016, 2018-2019 (q117)	
**Among the following groups of people, how many do you believe are involved in corruption? Tell me for each group if you believe it is none of them, few of them, most of them or all of them? Local authorities**	None of them<br>Few of them<br>Most of them<br>All of them	World Values Survey	2017-2020 (q115)	
**Please tell whether you agree or disagree with each of the following? There is corruption in the local**	Totally disagree<br>Tend to disagree<br>Tend to agree<br>Totally agree	Eurobarometers	2007 (QB1.3), 2011 (QC1.3), 2017 (QB15.1), 2019 (QB15.1), 2022 (QA15.1)	This question concerns the degree of corruption, while others ask about the number of corrupt individuals within a group. This leads to bias, as respondents are more likely to report corruption as a

**or regional public institutions in this country**				major problem than to report that all councillors are corrupt. This question was adjusted upward (less corruption) for comparability. The adjustment is based on overlap between WVS and Eurobarometers in 2017-2019 for Cyprus, Greece.
**Petty: Authorities**				
**In the past year, how often, if ever, have you had to pay a bribe, give a gift, or do a favor to government officials in order to: Avoid a problem with the police (like passing a checkpoint or avoiding a fine or arrest)?**	Never<br>Once or twice<br>A few times<br>Often	Afrobarometers<br>Transparency International<br>Democratic Republic of Congo	2008-2011 (Q51C), 2012-2013 (Q61D), 2014-2015 (Q55J), 2017-2019 (Q49T), 2019-2022 (Q44N)	While multiple answer options are preferable, other sources only provide binary responses. This leads to unfair comparisons, as binary responses by definition make corruption look more extreme. For comparability, this question has been made binary, where paying a bribe at least once is akin to 'yes' and no bribe at all to 'no'.
**Police officer asked for a bribe**	No<br>Yes	AmericasBarometer<br>LAPOP	2004, 2006-2010, 2012, 2014, 2016-2019 (exc2)	
**In the last 12 months, have you or anyone in your family been asked by a public official to give an informal gift or bribe in police?**	Yes<br>No	European Quality of Government Index	2010, 2013 (q16_3), 2017 (q17_3), 2021 (q18_3)	
**How often, if ever, did you have to pay a bribe, give a gift, or do a favour in order to get the assistance or services you needed from the police?**	Never<br>Once or twice<br>A few times<br>Often	Transparency International	2017 (Q9_5)	While multiple answer options are preferable, other sources only provide binary responses. This leads to unfair comparisons, as binary responses by definition make corruption look more extreme. For comparability, this question has been made binary, where paying a bribe at least once is akin to 'yes' and no bribe at all to 'no'.
**In the past 12 months has anyone asked you or expected you to give a gift, favour, or extra money for his or her services? Police, customs**	Yes<br>No	Eurobarometers	2007 (QB3.2.3), 2011 (QC5.2.3), 2017 (QB9b.1), 2019 (QB9b.1), 2022 (QA9b.1)	
**Petty: Government Officials**				

**Government employee asked for a bribe**	No<br>Yes	AmericasBarometer LAPOP	2004, 2006-2010, 2012, 2014, 2016-2019 (exc6)	
**How often do you think ordinary people like yourself or people from your neighbourhood have to pay a bribe, give a gift or do a favor to these people in order to get the services you need? Does it happen never, rarely, frequently or always?**	Never<br>Rarely<br>Frequently<br>Always	World Values Survey	2017-2020 (q118)	This question concerns perceptions on bribery in general. Other questions within this category concern personal bribery experiences. Corruption is much more likely to be perceived if the question includes perceptions on others' experiences. This question was adjusted upward (less corruption) for comparability. The adjustment is based on overlap between the Eurobarometers and the WVS in 2017-2018 for Germany.
**In the last five years, how often have you or a member of your immediate family come across a public official who hinted they wanted, or asked for, a bribe or favour in return for a service?**	Never<br>Seldom<br>Occasionally<br>Quite often<br>Very often	International Social Survey Programme	2006 (ZA4700:V62)	This question concerns personal bribery experiences, but also includes experiences of immediate family and asks about a longer timespan. This leads to a bias downward (more corruption). This question was adjusted upward (less corruption) for comparability. The adjustment is based on overlap between the ISSP and the LAPOP in 2006-2008 for Argentina and the Dominican Republic.
**Percent of firms expected to give gifts to public officials (to get things done)**	0 (None) to 100 (All)	World Bank Enterprise Surveys	2007, 2009, 2011-2013, 2016, 2019 (corr4)	This WBES question was adjusted based on a country-comparison between the WBES and the WVS based on overlap between Argentina, Bolivia, Cyprus. We only include WBES data if the country is not available in any other source.
**In the past 12 months has anyone asked you or expected you to give a gift, favour, or extra money for his or her services? Officials awarding public tenders + issuing building permits + issuing business permits + inspectors**	Yes<br>No	Eurobarometers	2007 (QB3.8.9.10.13), 2011 (QC5.8.9.10.13), 2017 (QB9b.8.9.10.13), 2019 (QB9b.8.9.10.13), 2022 (QA9b.8.9.10.13)	
**Petty: Judicial**				

**Asked to pay a bribe to the courts?**	Yes<br>No	Latin American Public Opinion Project	2004, 2006-2010, 2012, 2014, 2016-2019 (exc14)	
**How often, if ever, did you have to pay a bribe, give a gift, or do a favour in order to get the assistance or services you needed from the courts?**	Never<br>Once or twice<br>A few times<br>Often	Transparency International	2017 (TI_Q9_6)	While multiple answer options are preferable, other sources only provide binary responses. This leads to unfair comparisons, as binary responses by definition make corruption look more extreme. For comparability, this question has been made binary, where paying a bribe at least once is akin to 'yes' and no bribe at all to 'no'.
**In the past 12 months has anyone asked you or expected you to give a gift, favour, or extra money for his or her services? People workind in the judicial services (2007, 2011)/Courts (tribunals) (2017, 2019, 2022)**	Yes<br>No	Eurobarometers	2007 (QB3.4), 2011 (QC5.4), 2017 (QB9b.3), 2019 (QB9b.3), 2022 (QA9b.3)	
**Petty: Utilities**				
**In the past year, how often, if ever, have you had to pay a bribe, give a gift, or do a favor to government officials in order to: Get water or sanitation services?**	Never<br>Once or twice<br>A few times<br>Often	Afrobarometers<br>Transparency International<br>Democratic Republic of Congo	2008-2011 (Q51B), 2012-2013 (Q61B), 2014-2015 (Q55H)	
**How often, if ever, did you have to pay a bribe, give a gift, or do a favour in order to get the assistance or services you needed from a government service provider to get water, sanitation or electricity?**	Never<br>Once or twice<br>A few times<br>Often	Transparency International	2017 (Q9_4)	
**Percent of firms expected to give gifts to get an electrical connection**	0 (None) to 100 (All)	World Bank Enterprise Surveys	2007, 2009, 2011-2013, 2016, 2019 (corr6)	This WBES question was adjusted based on a country-comparison between the WBES and the Afrobarometers based on overlap between Namibia, Nigeria, Senegal. We only

				include WBES data if the country is not available in any other source.
**Petty: Education**				
**And how often, if ever, did you have to pay a bribe, give a gift, or do a favour for a teacher or school official in order to get the services you needed from the schools?**	Never<br>Once or twice<br>A few times<br>Often	Afrobarometers<br>Transparency International<br>Democratic Republic of Congo	2014-2015 (Q55B), 2017-2019(Q49C), 2019-2022 (Q44C)	While multiple answer options are preferable, other sources only provide binary responses. This leads to unfair comparisons, as binary responses by definition make corruption look more extreme. For comparability, this question has been made binary, where paying a bribe at least once is akin to 'yes' and no bribe at all to 'no'.
**Have you had to pay a bribe at school in the last twelve months?**	No<br>Yes	Latin American Public Opinion Project	2004, 2006-2010, 2012, 2014, 2016-2019 (exc16)	
**In the last 12 months, how often, if ever, did you have to pay a bribe, give a gift, or do a favour in order to get the assistance or services you needed from**	Never<br>Once or twice<br>A few times<br>Often	Transparency International	2017 (Q9_1)	While multiple answer options are preferable, other sources only provide binary responses. This leads to unfair comparisons, as binary responses by definition make corruption look more extreme. For comparability, this question has been made binary, where paying a bribe at least once is akin to 'yes' and no bribe at all to 'no'.
**In the last 12 months, have you or anyone in your family given an informal gift or bribe to education services?**	Yes<br>No	European Quality of Government Index	2010, 2013 (Q16_1), 2017 (Q18_1), 2021 (Q19_1)	This question concerns personal bribery experiences, but also includes experiences of family. This leads to a bias downward (more corruption). This question was adjusted upward (less corruption) for comparability. The adjustment is based on overlap between the EQoG and the Eurobarometers in 2017 for Belgium, Bulgaria, Czechia, Denmark, Finland, France, Germany, Greece, Hungary, Italy, Netherlands, Poland, Portugal, Romania, Slovakia, Spain, Sweden.
**In the past 12 months has anyone asked you or expected you to give a gift, favour, or extra money for his or her**	Yes<br>No	Eurobarometers	2007 (QB3.12), 2011 (QC5.12), 2017 (QB9b.12), 2019 (QB9b.12), 2022 (QA9b.12)	

**services? The education sector**				
**Petty: Documents**				
**And how often, if ever, did you have to pay a bribe, give a gift, or do a favour for a government official in order to get the document you needed?**	Never<br>Once or twice<br>A few times<br>Often	Afrobarometers<br>Transparency International<br>Democratic Republic of Congo	2008-2011 (Q51A), 2012-2013 (Q61A), 2014-2015 (Q55F), 2017-2019 (Q49K), 2019-2022 (Q44C)	While multiple answer options are preferable, other sources only provide binary responses. This leads to unfair comparisons, as binary responses by definition make corruption look more extreme. For comparability, this question has been made binary, where paying a bribe at least once is akin to 'yes' and no bribe at all to 'no'.
**Have you had to pay a bribe at the municipal government to process a document in the last twelve months?**	No<br>Yes	Latin American Public Opinion Project	2004, 2006-2010, 2012, 2014, 2016-2019 (exc11)	
**in the last 12 months. How often, if ever, did you have to pay a bribe, give a gift, or do a favour in order to get the assistance or services you needed from a government office to get the document you needed?**	Never<br>Once or twice<br>A few times<br>Often	Transparency International	2017 (Q9_3)	While multiple answer options are preferable, other sources only provide binary responses. This leads to unfair comparisons, as binary responses by definition make corruption look more extreme. For comparability, this question has been made binary, where paying a bribe at least once is akin to 'yes' and no bribe at all to 'no'.
**Percent of firms expected to give gifts to get a construction permit OR import license OR operating license**	0 (None) to 100 (All)	World Bank Enterprise Surveys	2007, 2009, 2011-2013, 2016, 2019 (corr8, corr9, corr10)	This WBES question was adjusted based on a country-comparison between the WBES and LAPOP based on overlap between Dominican Republic, Costa Rica, Colombia, Bolivia, Belize, Uruguay, Peru, Paraguay, Panama, Nicaragua, Mexico, Jamaica, Honduras, Haiti, El Salvador, Ecuador. We only include WBES data if the country is not available in any other source.
**Petty: Health**				
**And how often, if ever, did you have to pay a bribe, give a gift, or do a favour for a health worker or clinic or hospital staff in order to get**	Never<br>Once or twice<br>A few times<br>Often	Afrobarometers<br>Transparency International<br>Democratic Republic of Congo	2012-2013 (Q61C), 2014-2015 (Q55D), 2017-2019 (Q49G), 2019-2022 (Q44F)	While multiple answer options are preferable, other sources only provide binary responses. This leads to unfair comparisons, as binary responses by definition make corruption look more

**the medical care you needed?**				extreme. For comparability, this question has been made binary, where paying a bribe at least once is akin to 'yes' and no bribe at all to 'no'.
**In the last 12 months, have you or anyone in your family given an informal gift or bribe to health or medical services?**	Yes<br>No	European Quality of Government Index	2010, 2013 (Q16_2), 2017 (Q18_2), 2021 (Q19_2)	This question concerns personal bribery experiences, but also includes experiences of family. This leads to a bias downward (more corruption). This question was adjusted upward (less corruption) for comparability. The adjustment is based on overlap between the EQoG and the Eurobarometers in 2017 for Belgium, Bulgaria, Czechia, Denmark, Finland, France, Germany, Greece, Hungary, Italy, Netherlands, Poland, Portugal, Romania, Slovakia, Spain, Sweden.
**In order to be seen in a hospital or a clinic in the last twelve months, did you have to pay a bribe?**	No<br>Yes	Latin American Public Opinion Project	2004, 2006-2010, 2012, 2014, 2016-2019 (exc15)	
**in the last 12 months. How often, if ever, did you have to pay a bribe, give a gift, or do a favour in order to get the assistance or services you needed from a public clinic or hospital**	Never<br>Once or twice<br>A few times<br>Often	Transparency International	2017 (Q9_2)	While multiple answer options are preferable, other sources only provide binary responses. This leads to unfair comparisons, as binary responses by definition make corruption look more extreme. For comparability, this question has been made binary, where paying a bribe at least once is akin to 'yes' and no bribe at all to 'no'.
**In the past 12 months has anyone asked you or expected you to give a gift, favour, or extra money for his or her services? The health sector**	Yes<br>No	Eurobarometers	2007 (QB3.11), 2011 (QC5.11), 2017 (QB9b.11), 2019 (QB9b.11), 2022 (QA9b.11)	
**Petty: Elections**				
**During the last national election in [20xx], how often, if ever did a candidate or someone from a political party offer you something, like food or a gift or**	Never<br>Once or twice<br>A few times<br>Often	Afrobarometers	2012 (Q61F)	This question concerns personal election experiences. Other questions within this category concern perceptions on elections in general. Corruption is much more likely to be perceived if the question includes

**money, in return for your vote?**				perceptions on others' experiences. This question was adjusted downward (more corruption) for comparability. The adjustment is based on overlap between the Afrobarometers and the WVS in 2012 for Ghana and Zimbabwe.
**In your opinion, how often do the following things occur in this country's elections: Voters are bribed.**	Never<br>Once or twice<br>A few times<br>Often	Afrobarometers	2014-2015 (Q48D)	
**Corruption is NOT present in elections in my area.**	1 Strongly disagree<br>10 Strongly agree	European Quality of government Index	2010, 2013 (Q18), 2017 (Q19)	
**In your view, how often do the following things occur in this country's elections? Voters are bribed.**	Very often<br>Fairly often<br>Not often<br>Not at all often	World Values Survey	2011-2014 (V228D), 2017-2020 (Q227)	
**In the past 5 years, how many times, if at all, has anyone tried to offer you a bribe or special favour to vote in a particular way at a national, regional or local election? Has that happened to you…?**	Never<br>Once or twice<br>A few times<br>Often	Transparency International	2017 (Q19B)	This question concerns personal election experiences. Other questions within this category concern perceptions on elections in general. Corruption is much more likely to be perceived if the question includes perceptions on others' experiences. This question was adjusted downward (more corruption) for comparability. The adjustment is based on overlap between the Afrobarometers and the WVS in 2012 for Ghana and Zimbabwe.

Supplementary Table 3 – Overview of all 807 surveys.

Country	Year	Source	NGrand	NPetty	Dimensions
Afghanistan	2006	The Asia Foundation	6014	0	[Grand]: Severity
Afghanistan	2007	The Asia Foundation	6214	0	[Grand]: Severity; [Grand]: Local Government
Afghanistan	2008	The Asia Foundation	6474	0	[Grand]: Severity; [Grand]: Local Government
Afghanistan	2009	The Asia Foundation	6325	0	[Grand]: Severity; [Grand]: Local Government
Afghanistan	2010	The Asia Foundation	6402	0	[Grand]: Severity; [Grand]: Local Government
Afghanistan	2011	The Asia Foundation	6303	0	[Grand]: Severity; [Grand]: Local Government
Afghanistan	2012	The Asia Foundation	7881	0	[Grand]: Severity; [Grand]: Local Government
Afghanistan	2013	The Asia Foundation	9206	0	[Grand]: Severity; [Grand]: Local Government
Afghanistan	2014	The Asia Foundation	9232	0	[Grand]: Severity; [Grand]: Local Government
Afghanistan	2015	The Asia Foundation	9544	0	[Grand]: Severity; [Grand]: Local Government
Afghanistan	2016	The Asia Foundation	12600	0	[Grand]: Severity; [Grand]: Local Government
Afghanistan	2017	The Asia Foundation	9953	0	[Grand]: Severity
Afghanistan	2018	The Asia Foundation	14913	0	[Grand]: Severity
Afghanistan	2019	The Asia Foundation	17739	0	[Grand]: Severity
Albania	1998	World Value Surveys	808	0	[Grand]: Severity
Algeria	2011	Arabbarometers	1178	0	[Grand]: State
Algeria	2012	Afrobarometers	1168	1202	[Grand]: Executive; [Grand]: Legislative; [Grand]: Judicial; [Grand]: Taxation; [Grand]: Government Officials; [Grand]: Authorities; [Grand]: Local Government; [Petty]: Authorities; [Petty]: Utilities; [Petty]: Education; [Petty]: Documents; [Petty]: Health; [Petty]: Election
Algeria	2013	World Value Surveys	1200	730	[Grand]: State; [Petty]: Election
Algeria	2014	Arabbarometers	1093	0	[Grand]: State
Algeria	2015	Afrobarometers	1110	1153	[Grand]: Executive; [Grand]: Legislative; [Grand]: Judicial; [Grand]: Taxation; [Grand]: Government Officials; [Grand]: Authorities; [Grand]: Local Government; [Petty]: Authorities; [Petty]: Utilities; [Petty]: Education; [Petty]: Documents; [Petty]: Health; [Petty]: Election
Algeria	2017	Arabbarometers	1187	0	[Grand]: State
Algeria	2021	Arabbarometers	2966	0	[Grand]: State
Andorra	2018	World Value Surveys	984	994	[Grand]: State; [Grand]: Civil; [Grand]: Severity; [Grand]: Local Government; [Petty]: Government Officials; [Petty]: Election
Angola	2019	Afrobarometers	1977	2034	[Grand]: Executive; [Grand]: Legislative; [Grand]: Judicial; [Grand]: Taxation; [Grand]: Civil; [Grand]: Authorities; [Petty]: Authorities; [Petty]: Education; [Petty]: Documents; [Petty]: Health
Argentina	2000	Latinobarometers	1196	0	[Grand]: Severity
Argentina	2001	Latinobarometers	1196	0	[Grand]: Civil; [Grand]: Severity
Argentina	2006	International Social Survey Programme	1578	1589	[Grand]: Politician; [Grand]: Government Officials; [Petty]: Government Officials
Argentina	2008	LAPOP	1385	1478	[Grand]: Government Officials; [Petty]: Authorities; [Petty]: Government Officials; [Petty]: Judicial; [Petty]: Education; [Petty]: Documents; [Petty]: Health
Argentina	2013	World Value Surveys	0	942	[Petty]: Election
Argentina	2013	Latinobarometers	1132	0	[Grand]: State; [Grand]: Local Government
Argentina	2016	Latinobarometers	1165	0	[Grand]: Executive; [Grand]: Legislative; [Grand]: Judicial; [Grand]: Taxation; [Grand]: Government Officials; [Grand]: Authorities; [Grand]: Local Government
Argentina	2017	Latinobarometers	1183	0	[Grand]: Legislative; [Grand]: State; [Grand]: Judicial; [Grand]: Local Government
Argentina	2017	World Value Surveys	1002	977	[Grand]: State; [Grand]: Civil; [Grand]: Severity; [Grand]: Local Government; [Petty]: Government Officials; [Petty]: Election
Argentina	2018	Latinobarometers	1126	0	[Grand]: Executive; [Grand]: Legislative; [Grand]: Judicial; [Grand]: Taxation; [Grand]: Government Officials; [Grand]: Authorities; [Grand]: Local Government
Armenia	1997	World Value Surveys	1863	0	[Grand]: Severity
Australia	1995	World Value Surveys	1968	0	[Grand]: Severity
Australia	2006	International Social Survey Programme	2532	2631	[Grand]: Politician; [Grand]: Government Officials; [Petty]: Government Officials

Australia	2018	World Value Surveys	1805	1802	[Grand]: State; [Grand]: Civil; [Grand]: Severity; [Grand]: Local Government; [Petty]: Government Officials; [Petty]: Election
Austria	2007	Eurobarometers	1012	1012	[Grand]: State; [Grand]: Judicial; [Grand]: Government Officials; [Grand]: Authorities; [Grand]: Local Government; [Petty]: Authorities; [Petty]: Government Officials; [Petty]: Judicial; [Petty]: Education; [Petty]: Health
Austria	2011	Eurobarometers	1018	1018	[Grand]: State; [Grand]: Judicial; [Grand]: Government Officials; [Grand]: Authorities; [Grand]: Local Government; [Petty]: Authorities; [Petty]: Government Officials; [Petty]: Judicial; [Petty]: Education; [Petty]: Health
Austria	2017	Eurobarometers	1012	1012	[Grand]: State; [Grand]: Judicial; [Grand]: Government Officials; [Grand]: Authorities; [Grand]: Local Government; [Petty]: Authorities; [Petty]: Government Officials; [Petty]: Judicial; [Petty]: Education; [Petty]: Health
Austria	2019	Eurobarometers	1018	1018	[Grand]: State; [Grand]: Judicial; [Grand]: Government Officials; [Grand]: Authorities; [Grand]: Local Government; [Petty]: Authorities; [Petty]: Government Officials; [Petty]: Judicial; [Petty]: Education; [Petty]: Health
Austria	2021	Europe Quality of Government QoG	4097	4501	[Grand]: Authorities; [Petty]: Authorities; [Petty]: Education; [Petty]: Health
Austria	2022	Eurobarometers	1011	1011	[Grand]: State; [Grand]: Judicial; [Grand]: Government Officials; [Grand]: Authorities; [Grand]: Local Government; [Petty]: Authorities; [Petty]: Government Officials; [Petty]: Judicial; [Petty]: Education; [Petty]: Health
Azerbaijan	1997	World Value Surveys	1156	0	[Grand]: Severity
Azerbaijan	2011	World Value Surveys	0	867	[Petty]: Election
Bahamas	2014	LAPOP	0	3420	[Petty]: Authorities; [Petty]: Government Officials; [Petty]: Judicial; [Petty]: Education; [Petty]: Health
Bangladesh	1996	World Value Surveys	1414	0	[Grand]: Severity
Bangladesh	2018	World Value Surveys	1200	1176	[Grand]: State; [Grand]: Civil; [Grand]: Severity; [Grand]: Local Government; [Petty]: Government Officials; [Petty]: Election
Barbados	2014	LAPOP	0	3823	[Petty]: Authorities; [Petty]: Government Officials; [Petty]: Judicial; [Petty]: Education; [Petty]: Health
Belarus	1996	World Value Surveys	1962	0	[Grand]: Severity
Belgium	2007	Eurobarometers	1004	1004	[Grand]: State; [Grand]: Judicial; [Grand]: Government Officials; [Grand]: Authorities; [Grand]: Local Government; [Petty]: Authorities; [Petty]: Government Officials; [Petty]: Judicial; [Petty]: Education; [Petty]: Health
Belgium	2011	Eurobarometers	1028	1028	[Grand]: State; [Grand]: Judicial; [Grand]: Government Officials; [Grand]: Authorities; [Grand]: Local Government; [Petty]: Authorities; [Petty]: Government Officials; [Petty]: Judicial; [Petty]: Education; [Petty]: Health
Belgium	2017	Eurobarometers	1005	1005	[Grand]: State; [Grand]: Judicial; [Grand]: Taxation; [Grand]: Government Officials; [Grand]: Authorities; [Grand]: Local Government; [Petty]: Authorities; [Petty]: Government Officials; [Petty]: Judicial; [Petty]: Education; [Petty]: Health
Belgium	2017	Europe Quality of Government QoG	1254	1350	[Grand]: Authorities; [Petty]: Authorities; [Petty]: Education; [Petty]: Health; [Petty]: Election
Belgium	2019	Eurobarometers	1007	1007	[Grand]: State; [Grand]: Judicial; [Grand]: Taxation; [Grand]: Government Officials; [Grand]: Authorities; [Grand]: Local Government; [Petty]: Authorities; [Petty]: Government Officials; [Petty]: Judicial; [Petty]: Education; [Petty]: Health
Belgium	2021	Europe Quality of Government QoG	4870	5643	[Grand]: Authorities; [Petty]: Authorities; [Petty]: Education; [Petty]: Health
Belgium	2022	Eurobarometers	1018	1018	[Grand]: State; [Grand]: Judicial; [Grand]: Taxation; [Grand]: Government Officials; [Grand]: Authorities; [Grand]: Local Government; [Petty]: Authorities; [Petty]: Government Officials; [Petty]: Judicial; [Petty]: Education; [Petty]: Health
Belize	2008	LAPOP	1411	1539	[Grand]: Government Officials; [Petty]: Authorities; [Petty]: Government Officials; [Petty]: Judicial; [Petty]: Education; [Petty]: Documents; [Petty]: Health
Belize	2010	LAPOP	1453	1504	[Grand]: Government Officials; [Petty]: Authorities; [Petty]: Government Officials; [Petty]: Judicial; [Petty]: Education; [Petty]: Documents; [Petty]: Health
Belize	2012	LAPOP	1447	1510	[Grand]: Government Officials; [Petty]: Authorities; [Petty]: Government Officials; [Petty]: Judicial; [Petty]: Education; [Petty]: Documents; [Petty]: Health
Belize	2014	LAPOP	1509	1532	[Grand]: Government Officials; [Petty]: Authorities; [Petty]: Government Officials; [Petty]: Judicial; [Petty]: Education; [Petty]: Documents; [Petty]: Health

Benin	2008	Afrobarometers	1125	656	[Grand]: Executive; [Grand]: Legislative; [Grand]: Judicial; [Grand]: Taxation; [Grand]: Government Officials; [Grand]: Authorities; [Grand]: Local Government; [Petty]: Authorities; [Petty]: Utilities; [Petty]: Documents
Benin	2012	Afrobarometers	1184	1200	[Grand]: Executive; [Grand]: Legislative; [Grand]: Judicial; [Grand]: Taxation; [Grand]: Government Officials; [Grand]: Authorities; [Grand]: Local Government; [Petty]: Authorities; [Petty]: Utilities; [Petty]: Education; [Petty]: Documents; [Petty]: Health; [Petty]: Election
Benin	2014	Afrobarometers	1182	1190	[Grand]: Executive; [Grand]: Legislative; [Grand]: Judicial; [Grand]: Taxation; [Grand]: Government Officials; [Grand]: Authorities; [Grand]: Local Government; [Petty]: Authorities; [Petty]: Utilities; [Petty]: Education; [Petty]: Documents; [Petty]: Health; [Petty]: Election
Benin	2016	Afrobarometers	1187	1200	[Grand]: Executive; [Grand]: Legislative; [Grand]: Judicial; [Grand]: Government Officials; [Grand]: Authorities; [Grand]: Local Government; [Petty]: Authorities; [Petty]: Education; [Petty]: Documents; [Petty]: Health
Benin	2020	Afrobarometers	1196	966	[Grand]: Executive; [Grand]: Legislative; [Grand]: Judicial; [Grand]: Taxation; [Grand]: Civil; [Grand]: Authorities; [Grand]: Local Government; [Petty]: Authorities; [Petty]: Education; [Petty]: Documents; [Petty]: Health
Bhutan	2019	World Bank Country Group Surveys	120	0	[Grand]: State
Bolivia	1998	Latinobarometers	791	0	[Grand]: Severity
Bolivia	2000	Latinobarometers	1067	0	[Grand]: Severity
Bolivia	2001	Latinobarometers	1068	0	[Grand]: Civil; [Grand]: Severity
Bolivia	2004	LAPOP	2996	3067	[Grand]: Government Officials; [Petty]: Authorities; [Petty]: Government Officials; [Petty]: Judicial; [Petty]: Education; [Petty]: Documents; [Petty]: Health
Bolivia	2006	LAPOP	2802	2993	[Grand]: Government Officials; [Petty]: Authorities; [Petty]: Government Officials; [Petty]: Judicial; [Petty]: Education; [Petty]: Documents; [Petty]: Health
Bolivia	2008	LAPOP	2807	2741	[Grand]: Government Officials; [Petty]: Authorities; [Petty]: Government Officials; [Petty]: Judicial; [Petty]: Education; [Petty]: Documents; [Petty]: Health
Bolivia	2013	Latinobarometers	1119	0	[Grand]: State; [Grand]: Local Government
Bolivia	2016	Latinobarometers	1095	0	[Grand]: Executive; [Grand]: Legislative; [Grand]: Judicial; [Grand]: Taxation; [Grand]: Government Officials; [Grand]: Authorities; [Grand]: Local Government
Bolivia	2017	World Value Surveys	2055	2028	[Grand]: State; [Grand]: Civil; [Grand]: Severity; [Grand]: Local Government; [Petty]: Government Officials; [Petty]: Election
Bolivia	2017	Latinobarometers	1187	0	[Grand]: Legislative; [Grand]: State; [Grand]: Judicial; [Grand]: Local Government
Bolivia	2018	Latinobarometers	1114	0	[Grand]: Executive; [Grand]: Legislative; [Grand]: Judicial; [Grand]: Taxation; [Grand]: Government Officials; [Grand]: Authorities; [Grand]: Local Government
Bosnia and Herzegovina	2009	World Bank Enterprise Surveys	247	330	[Grand]: State; [Grand]: Taxation; [Petty]: Government Officials; [Petty]: Utilities; [Petty]: Documents
Bosnia and Herzegovina	2013	World Bank Enterprise Surveys	275	357	[Grand]: State; [Grand]: Taxation; [Petty]: Government Officials; [Petty]: Utilities; [Petty]: Documents
Bosnia and Herzegovina	2019	World Bank Enterprise Surveys	232	350	[Grand]: State; [Grand]: Taxation; [Petty]: Government Officials; [Petty]: Utilities; [Petty]: Documents
Botswana	2008	Afrobarometers	1086	1123	[Grand]: Executive; [Grand]: Legislative; [Grand]: Judicial; [Grand]: Taxation; [Grand]: Government Officials; [Grand]: Authorities; [Grand]: Local Government; [Petty]: Authorities; [Petty]: Utilities; [Petty]: Documents
Botswana	2012	Afrobarometers	1120	1192	[Grand]: Executive; [Grand]: Legislative; [Grand]: Judicial; [Grand]: Taxation; [Grand]: Government Officials; [Grand]: Authorities; [Grand]: Local Government; [Petty]: Authorities; [Petty]: Utilities; [Petty]: Education; [Petty]: Documents; [Petty]: Health; [Petty]: Election
Botswana	2014	Afrobarometers	1129	1182	[Grand]: Executive; [Grand]: Legislative; [Grand]: Judicial; [Grand]: Taxation; [Grand]: Government Officials; [Grand]: Authorities; [Grand]: Local Government; [Petty]: Authorities; [Petty]: Utilities; [Petty]: Education; [Petty]: Documents; [Petty]: Health; [Petty]: Election
Botswana	2017	Afrobarometers	1081	1198	[Grand]: Executive; [Grand]: Legislative; [Grand]: Judicial; [Grand]: Government Officials; [Grand]: Authorities; [Grand]: Local Government; [Petty]: Authorities; [Petty]: Education; [Petty]: Documents; [Petty]: Health

Botswana	2019	Afrobarometers	1110	1095	[Grand]: Executive; [Grand]: Legislative; [Grand]: Judicial; [Grand]: Taxation; [Grand]: Civil; [Grand]: Authorities; [Grand]: Local Government; [Petty]: Authorities; [Petty]: Education; [Petty]: Documents; [Petty]: Health
Brazil	2010	LAPOP	2360	2472	[Grand]: Government Officials; [Petty]: Authorities; [Petty]: Government Officials; [Petty]: Judicial; [Petty]: Education; [Petty]: Documents; [Petty]: Health
Brazil	2012	LAPOP	1388	1499	[Grand]: Government Officials; [Petty]: Authorities; [Petty]: Government Officials; [Petty]: Judicial; [Petty]: Education; [Petty]: Documents; [Petty]: Health
Brazil	2014	World Value Surveys	0	1359	[Petty]: Election
Brazil	2014	LAPOP	0	1500	[Petty]: Authorities; [Petty]: Government Officials; [Petty]: Judicial; [Petty]: Education; [Petty]: Documents; [Petty]: Health
Brazil	2017	Latinobarometers	1183	0	[Grand]: Legislative; [Grand]: State; [Grand]: Judicial; [Grand]: Local Government
Brazil	2018	World Value Surveys	1751	1700	[Grand]: State; [Grand]: Civil; [Grand]: Severity; [Grand]: Local Government; [Petty]: Government Officials; [Petty]: Election
Bulgaria	1997	World Value Surveys	792	0	[Grand]: Severity
Bulgaria	2007	Eurobarometers	1000	1000	[Grand]: State; [Grand]: Judicial; [Grand]: Government Officials; [Grand]: Authorities; [Grand]: Local Government; [Petty]: Authorities; [Petty]: Government Officials; [Petty]: Judicial; [Petty]: Education; [Petty]: Health
Bulgaria	2010	Europe Quality of Government QoG	1170	1170	[Grand]: Authorities; [Petty]: Authorities; [Petty]: Education; [Petty]: Health; [Petty]: Election
Bulgaria	2011	Eurobarometers	1006	1006	[Grand]: State; [Grand]: Judicial; [Grand]: Government Officials; [Grand]: Authorities; [Grand]: Local Government; [Petty]: Authorities; [Petty]: Government Officials; [Petty]: Judicial; [Petty]: Education; [Petty]: Health
Bulgaria	2013	Europe Quality of Government QoG	2392	2402	[Grand]: Authorities; [Petty]: Authorities; [Petty]: Education; [Petty]: Health; [Petty]: Election
Bulgaria	2017	Eurobarometers	1027	1027	[Grand]: State; [Grand]: Judicial; [Grand]: Taxation; [Grand]: Government Officials; [Grand]: Authorities; [Grand]: Local Government; [Petty]: Authorities; [Petty]: Government Officials; [Petty]: Judicial; [Petty]: Education; [Petty]: Health
Bulgaria	2017	Europe Quality of Government QoG	2320	2400	[Grand]: Authorities; [Petty]: Authorities; [Petty]: Education; [Petty]: Health; [Petty]: Election
Bulgaria	2019	Eurobarometers	1028	1028	[Grand]: State; [Grand]: Judicial; [Grand]: Taxation; [Grand]: Government Officials; [Grand]: Authorities; [Grand]: Local Government; [Petty]: Authorities; [Petty]: Government Officials; [Petty]: Judicial; [Petty]: Education; [Petty]: Health
Bulgaria	2021	Europe Quality of Government QoG	2821	3056	[Grand]: Authorities; [Petty]: Authorities; [Petty]: Education; [Petty]: Health
Bulgaria	2022	Eurobarometers	1040	1040	[Grand]: State; [Grand]: Judicial; [Grand]: Taxation; [Grand]: Government Officials; [Grand]: Authorities; [Grand]: Local Government; [Petty]: Authorities; [Petty]: Government Officials; [Petty]: Judicial; [Petty]: Education; [Petty]: Health
Burkina Faso	2008	Afrobarometers	1042	813	[Grand]: Executive; [Grand]: Legislative; [Grand]: Judicial; [Grand]: Taxation; [Grand]: Government Officials; [Grand]: Authorities; [Grand]: Local Government; [Petty]: Authorities; [Petty]: Utilities; [Petty]: Documents
Burkina Faso	2012	Afrobarometers	1080	1197	[Grand]: Executive; [Grand]: Legislative; [Grand]: Judicial; [Grand]: Taxation; [Grand]: Government Officials; [Grand]: Authorities; [Grand]: Local Government; [Petty]: Authorities; [Petty]: Utilities; [Petty]: Education; [Petty]: Documents; [Petty]: Health; [Petty]: Election
Burkina Faso	2015	Afrobarometers	1180	1181	[Grand]: Executive; [Grand]: Legislative; [Grand]: Judicial; [Grand]: Taxation; [Grand]: Government Officials; [Grand]: Authorities; [Grand]: Local Government; [Petty]: Authorities; [Petty]: Utilities; [Petty]: Education; [Petty]: Documents; [Petty]: Health; [Petty]: Election
Burkina Faso	2017	Afrobarometers	1143	1200	[Grand]: Executive; [Grand]: Legislative; [Grand]: Judicial; [Grand]: Government Officials; [Grand]: Authorities; [Grand]: Local Government; [Petty]: Authorities; [Petty]: Education; [Petty]: Documents; [Petty]: Health
Burkina Faso	2019	Afrobarometers	1173	984	[Grand]: Executive; [Grand]: Legislative; [Grand]: Judicial; [Grand]: Taxation; [Grand]: Civil; [Grand]: Authorities; [Grand]: Local Government; [Petty]: Authorities; [Petty]: Education; [Petty]: Documents; [Petty]: Health

Burundi	2012	Afrobarometers	1153	1197	[Grand]: Executive; [Grand]: Legislative; [Grand]: Judicial; [Grand]: Taxation; [Grand]: Government Officials; [Grand]: Authorities; [Grand]: Local Government; [Petty]: Authorities; [Petty]: Utilities; [Petty]: Education; [Petty]: Documents; [Petty]: Health; [Petty]: Election
Burundi	2014	Afrobarometers	1164	1175	[Grand]: Executive; [Grand]: Legislative; [Grand]: Judicial; [Grand]: Taxation; [Grand]: Government Officials; [Grand]: Authorities; [Grand]: Local Government; [Petty]: Authorities; [Petty]: Utilities; [Petty]: Education; [Petty]: Documents; [Petty]: Health; [Petty]: Election
Cambodia	2015	Asiabarometers	1187	0	[Grand]: State; [Grand]: Local Government
Cameroon	2012	Afrobarometers	1086	1148	[Grand]: Executive; [Grand]: Legislative; [Grand]: Judicial; [Grand]: Taxation; [Grand]: Government Officials; [Grand]: Authorities; [Grand]: Local Government; [Petty]: Authorities; [Petty]: Utilities; [Petty]: Education; [Petty]: Documents; [Petty]: Health; [Petty]: Election
Cameroon	2015	Afrobarometers	1121	1143	[Grand]: Executive; [Grand]: Legislative; [Grand]: Judicial; [Grand]: Taxation; [Grand]: Government Officials; [Grand]: Authorities; [Grand]: Local Government; [Petty]: Authorities; [Petty]: Utilities; [Petty]: Education; [Petty]: Documents; [Petty]: Health; [Petty]: Election
Cameroon	2018	Afrobarometers	1135	1202	[Grand]: Executive; [Grand]: Legislative; [Grand]: Judicial; [Grand]: Government Officials; [Grand]: Authorities; [Grand]: Local Government; [Petty]: Authorities; [Petty]: Education; [Petty]: Documents; [Petty]: Health
Cameroon	2021	Afrobarometers	1193	1041	[Grand]: Executive; [Grand]: Legislative; [Grand]: Judicial; [Grand]: Taxation; [Grand]: Civil; [Grand]: Authorities; [Grand]: Local Government; [Petty]: Authorities; [Petty]: Education; [Petty]: Documents; [Petty]: Health
Canada	2006	International Social Survey Programme	843	858	[Grand]: Politician; [Grand]: Government Officials; [Petty]: Government Officials
Canada	2008	LAPOP	1988	0	[Grand]: Government Officials
Cape Verde	2008	Afrobarometers	747	1211	[Grand]: Executive; [Grand]: Legislative; [Grand]: Judicial; [Grand]: Taxation; [Grand]: Government Officials; [Grand]: Authorities; [Grand]: Local Government; [Petty]: Authorities; [Petty]: Utilities; [Petty]: Documents
Cape Verde	2012	Afrobarometers	935	1187	[Grand]: Executive; [Grand]: Legislative; [Grand]: Judicial; [Grand]: Taxation; [Grand]: Government Officials; [Grand]: Authorities; [Grand]: Local Government; [Petty]: Authorities; [Petty]: Utilities; [Petty]: Education; [Petty]: Documents; [Petty]: Health; [Petty]: Election
Cape Verde	2014	Afrobarometers	841	1140	[Grand]: Executive; [Grand]: Legislative; [Grand]: Judicial; [Grand]: Taxation; [Grand]: Government Officials; [Grand]: Authorities; [Grand]: Local Government; [Petty]: Authorities; [Petty]: Utilities; [Petty]: Education; [Petty]: Documents; [Petty]: Health; [Petty]: Election
Cape Verde	2017	Afrobarometers	1018	1200	[Grand]: Executive; [Grand]: Legislative; [Grand]: Judicial; [Grand]: Government Officials; [Grand]: Authorities; [Grand]: Local Government; [Petty]: Authorities; [Petty]: Education; [Petty]: Documents; [Petty]: Health
Cape Verde	2019	Afrobarometers	986	1090	[Grand]: Executive; [Grand]: Legislative; [Grand]: Judicial; [Grand]: Taxation; [Grand]: Civil; [Grand]: Authorities; [Grand]: Local Government; [Petty]: Authorities; [Petty]: Education; [Petty]: Documents; [Petty]: Health
Central African Republic	2020	World Bank Country Group Surveys	251	0	[Grand]: State
Chad	2018	World Bank Country Group Surveys	308	0	[Grand]: State
Chile	1998	Latinobarometers	1178	0	[Grand]: Severity
Chile	2000	Latinobarometers	1159	0	[Grand]: Severity
Chile	2001	Latinobarometers	1158	0	[Grand]: Civil; [Grand]: Severity
Chile	2006	International Social Survey Programme	1392	1451	[Grand]: Politician; [Grand]: Government Officials; [Petty]: Government Officials
Chile	2016	Latinobarometers	1124	0	[Grand]: Executive; [Grand]: Legislative; [Grand]: Judicial; [Grand]: Taxation; [Grand]: Government Officials; [Grand]: Authorities; [Grand]: Local Government
Chile	2017	Latinobarometers	1150	0	[Grand]: Legislative; [Grand]: State; [Grand]: Judicial; [Grand]: Local Government
Chile	2018	World Value Surveys	995	961	[Grand]: State; [Grand]: Civil; [Grand]: Severity; [Grand]: Local Government; [Petty]: Government Officials; [Petty]: Election
Chile	2018	Latinobarometers	1134	0	[Grand]: Executive; [Grand]: Legislative; [Grand]: Judicial; [Grand]: Taxation; [Grand]: Government Officials; [Grand]: Authorities; [Grand]: Local Government
China	2006	International Social Survey Programme	1858	1901	[Grand]: Politician; [Grand]: Government Officials; [Petty]: Government Officials

China	2012	World Value Surveys	0	2007	[Petty]: Election
China	2018	Asiabarometers	1149	0	[Grand]: State; [Grand]: Local Government
China	2018	World Value Surveys	5100	5075	[Grand]: State; [Grand]: Civil; [Grand]: Severity; [Grand]: Local Government; [Petty]: Government Officials; [Petty]: Election
Colombia	2000	Latinobarometers	1198	0	[Grand]: Severity
Colombia	2001	Latinobarometers	1196	0	[Grand]: Civil; [Grand]: Severity
Colombia	2004	LAPOP	1336	1477	[Grand]: Government Officials; [Petty]: Authorities; [Petty]: Government Officials; [Petty]: Judicial; [Petty]: Education; [Petty]: Documents; [Petty]: Health
Colombia	2006	LAPOP	1389	1491	[Grand]: Government Officials; [Petty]: Authorities; [Petty]: Government Officials; [Petty]: Judicial; [Petty]: Education; [Petty]: Documents; [Petty]: Health
Colombia	2008	LAPOP	1419	1503	[Grand]: Government Officials; [Petty]: Authorities; [Petty]: Government Officials; [Petty]: Judicial; [Petty]: Education; [Petty]: Documents; [Petty]: Health
Colombia	2010	LAPOP	1436	1506	[Grand]: Government Officials; [Petty]: Authorities; [Petty]: Government Officials; [Petty]: Judicial; [Petty]: Education; [Petty]: Documents; [Petty]: Health
Colombia	2012	World Value Surveys	0	1442	[Petty]: Election
Colombia	2012	LAPOP	1403	1511	[Grand]: Government Officials; [Petty]: Authorities; [Petty]: Government Officials; [Petty]: Judicial; [Petty]: Education; [Petty]: Documents; [Petty]: Health
Colombia	2013	Latinobarometers	1162	0	[Grand]: State; [Grand]: Local Government
Colombia	2014	LAPOP	1451	1496	[Grand]: Government Officials; [Petty]: Authorities; [Petty]: Government Officials; [Petty]: Judicial; [Petty]: Education; [Petty]: Documents; [Petty]: Health
Colombia	2016	Latinobarometers	1142	0	[Grand]: Executive; [Grand]: Legislative; [Grand]: Judicial; [Grand]: Taxation; [Grand]: Government Officials; [Grand]: Authorities; [Grand]: Local Government
Colombia	2017	Latinobarometers	1193	0	[Grand]: Legislative; [Grand]: State; [Grand]: Judicial; [Grand]: Local Government
Colombia	2018	LAPOP	1613	1663	[Grand]: Politician; [Grand]: Government Officials; [Petty]: Authorities; [Petty]: Government Officials; [Petty]: Judicial; [Petty]: Education; [Petty]: Documents; [Petty]: Health
Colombia	2018	Latinobarometers	1116	0	[Grand]: Executive; [Grand]: Legislative; [Grand]: Judicial; [Grand]: Taxation; [Grand]: Government Officials; [Grand]: Authorities; [Grand]: Local Government
Colombia	2018	World Value Surveys	1520	1520	[Grand]: State; [Grand]: Civil; [Grand]: Severity; [Grand]: Local Government; [Petty]: Government Officials; [Petty]: Election
Comoros	2013	World Bank Country Group Surveys	126	0	[Grand]: State
Comoros	2016	World Bank Country Group Surveys	134	0	[Grand]: State
Comoros	2018	World Bank Country Group Surveys	144	0	[Grand]: State
Comoros	2021	World Bank Country Group Surveys	222	0	[Grand]: State
Congo Brazzaville	2015	World Bank Country Group Surveys	257	0	[Grand]: State
Congo Brazzaville	2018	World Bank Country Group Surveys	304	0	[Grand]: State
Congo Democratic Republic	2017	Transparency International, AfrobarometersR8	1269	1111	[Grand]: Executive; [Grand]: Legislative; [Grand]: Judicial; [Grand]: Government Officials; [Grand]: Authorities; [Grand]: Local Government; [Petty]: Authorities; [Petty]: Utilities; [Petty]: Education; [Petty]: Documents; [Petty]: Health
Cook Islands	2017	Transparency International	274	277	[Grand]: Executive; [Grand]: Legislative; [Grand]: State; [Grand]: Judicial; [Grand]: Civil; [Grand]: Authorities; [Grand]: Local Government; [Petty]: Authorities; [Petty]: Judicial; [Petty]: Utilities; [Petty]: Education; [Petty]: Documents; [Petty]: Health; [Petty]: Election
Costa Rica	1998	Latinobarometers	979	0	[Grand]: Severity
Costa Rica	2000	Latinobarometers	968	0	[Grand]: Severity
Costa Rica	2001	Latinobarometers	995	0	[Grand]: Civil; [Grand]: Severity
Costa Rica	2004	LAPOP	1437	1500	[Grand]: Government Officials; [Petty]: Authorities; [Petty]: Government Officials; [Petty]: Judicial; [Petty]: Education; [Petty]: Documents; [Petty]: Health

Costa Rica	2006	LAPOP	1466	1497	[Grand]: Government Officials; [Petty]: Authorities; [Petty]: Government Officials; [Petty]: Judicial; [Petty]: Education; [Petty]: Documents; [Petty]: Health
Costa Rica	2008	LAPOP	1421	1494	[Grand]: Government Officials; [Petty]: Authorities; [Petty]: Government Officials; [Petty]: Judicial; [Petty]: Education; [Petty]: Documents; [Petty]: Health
Costa Rica	2010	LAPOP	1459	1492	[Grand]: Government Officials; [Petty]: Authorities; [Petty]: Government Officials; [Petty]: Judicial; [Petty]: Education; [Petty]: Documents; [Petty]: Health
Costa Rica	2012	LAPOP	1468	1496	[Grand]: Government Officials; [Petty]: Authorities; [Petty]: Government Officials; [Petty]: Judicial; [Petty]: Education; [Petty]: Documents; [Petty]: Health
Costa Rica	2013	Latinobarometers	972	0	[Grand]: State; [Grand]: Local Government
Costa Rica	2014	LAPOP	0	1535	[Petty]: Authorities; [Petty]: Government Officials; [Petty]: Judicial; [Petty]: Education; [Petty]: Documents; [Petty]: Health
Costa Rica	2017	Latinobarometers	979	0	[Grand]: Legislative; [Grand]: State; [Grand]: Judicial; [Grand]: Local Government
Costa Rica	2018	Latinobarometers	938	0	[Grand]: Executive; [Grand]: Legislative; [Grand]: Judicial; [Grand]: Taxation; [Grand]: Government Officials; [Grand]: Authorities; [Grand]: Local Government
Croatia	2017	Eurobarometers	1038	1038	[Grand]: State; [Grand]: Judicial; [Grand]: Taxation; [Grand]: Government Officials; [Grand]: Authorities; [Grand]: Local Government; [Petty]: Authorities; [Petty]: Government Officials; [Petty]: Judicial; [Petty]: Education; [Petty]: Health
Croatia	2019	Eurobarometers	1029	1029	[Grand]: State; [Grand]: Judicial; [Grand]: Taxation; [Grand]: Government Officials; [Grand]: Authorities; [Grand]: Local Government; [Petty]: Authorities; [Petty]: Government Officials; [Petty]: Judicial; [Petty]: Education; [Petty]: Health
Cyprus	2007	Eurobarometers	505	505	[Grand]: State; [Grand]: Judicial; [Grand]: Government Officials; [Grand]: Authorities; [Grand]: Local Government; [Petty]: Authorities; [Petty]: Government Officials; [Petty]: Judicial; [Petty]: Education; [Petty]: Health
Cyprus	2011	Eurobarometers	506	506	[Grand]: State; [Grand]: Judicial; [Grand]: Government Officials; [Grand]: Authorities; [Grand]: Local Government; [Petty]: Authorities; [Petty]: Government Officials; [Petty]: Judicial; [Petty]: Education; [Petty]: Health
Cyprus	2017	Eurobarometers	502	502	[Grand]: State; [Grand]: Judicial; [Grand]: Taxation; [Grand]: Government Officials; [Grand]: Authorities; [Grand]: Local Government; [Petty]: Authorities; [Petty]: Government Officials; [Petty]: Judicial; [Petty]: Education; [Petty]: Health
Cyprus	2019	Eurobarometers	504	504	[Grand]: State; [Grand]: Judicial; [Grand]: Taxation; [Grand]: Government Officials; [Grand]: Authorities; [Grand]: Local Government; [Petty]: Authorities; [Petty]: Government Officials; [Petty]: Judicial; [Petty]: Education; [Petty]: Health
Cyprus	2019	World Value Surveys	979	954	[Grand]: State; [Grand]: Civil; [Grand]: Severity; [Grand]: Local Government; [Petty]: Government Officials; [Petty]: Election
Cyprus	2022	Eurobarometers	504	504	[Grand]: State; [Grand]: Judicial; [Grand]: Taxation; [Grand]: Government Officials; [Grand]: Authorities; [Grand]: Local Government; [Petty]: Authorities; [Petty]: Government Officials; [Petty]: Judicial; [Petty]: Education; [Petty]: Health
Czech Republic	2006	International Social Survey Programme	1143	1099	[Grand]: Politician; [Grand]: Government Officials; [Petty]: Government Officials
Czech Republic	2007	Eurobarometers	1169	1169	[Grand]: State; [Grand]: Judicial; [Grand]: Government Officials; [Grand]: Authorities; [Grand]: Local Government; [Petty]: Authorities; [Petty]: Government Officials; [Petty]: Judicial; [Petty]: Education; [Petty]: Health
Czech Republic	2010	Europe Quality of Government QoG	1560	1560	[Grand]: Authorities; [Petty]: Authorities; [Petty]: Education; [Petty]: Health; [Petty]: Election
Czech Republic	2011	Eurobarometers	1069	1069	[Grand]: State; [Grand]: Judicial; [Grand]: Government Officials; [Grand]: Authorities; [Grand]: Local Government; [Petty]: Authorities; [Petty]: Government Officials; [Petty]: Judicial; [Petty]: Education; [Petty]: Health
Czech Republic	2013	Europe Quality of Government QoG	3236	3236	[Grand]: Authorities; [Petty]: Authorities; [Petty]: Education; [Petty]: Health; [Petty]: Election
Czech Republic	2017	Eurobarometers	1027	1027	[Grand]: State; [Grand]: Judicial; [Grand]: Taxation; [Grand]: Government Officials; [Grand]: Authorities; [Grand]: Local Government; [Petty]: Authorities; [Petty]: Government Officials; [Petty]: Judicial; [Petty]: Education; [Petty]: Health
Czech Republic	2017	Europe Quality of Government QoG	3585	3600	[Grand]: Authorities; [Petty]: Authorities; [Petty]: Education; [Petty]: Health; [Petty]: Election

Czech Republic	2019	Eurobarometers	1001	1001	[Grand]: State; [Grand]: Judicial; [Grand]: Taxation; [Grand]: Government Officials; [Grand]: Authorities; [Grand]: Local Government; [Petty]: Authorities; [Petty]: Government Officials; [Petty]: Judicial; [Petty]: Education; [Petty]: Health
Czech Republic	2021	Europe Quality of Government QoG	4202	4913	[Grand]: Authorities; [Petty]: Authorities; [Petty]: Education; [Petty]: Health
Czech Republic	2022	Eurobarometers	1033	1033	[Grand]: State; [Grand]: Judicial; [Grand]: Taxation; [Grand]: Government Officials; [Grand]: Authorities; [Grand]: Local Government; [Petty]: Authorities; [Petty]: Government Officials; [Petty]: Judicial; [Petty]: Education; [Petty]: Health
Côte d'Ivoire	2017	Afrobarometers	1150	1200	[Grand]: Executive; [Grand]: Legislative; [Grand]: Judicial; [Grand]: Government Officials; [Grand]: Authorities; [Grand]: Local Government; [Petty]: Authorities; [Petty]: Education; [Petty]: Documents; [Petty]: Health
Côte d'Ivoire	2019	Afrobarometers	1194	959	[Grand]: Executive; [Grand]: Legislative; [Grand]: Judicial; [Grand]: Taxation; [Grand]: Civil; [Grand]: Authorities; [Grand]: Local Government; [Petty]: Authorities; [Petty]: Education; [Petty]: Documents; [Petty]: Health
Denmark	2006	International Social Survey Programme	1184	1314	[Grand]: Politician; [Grand]: Government Officials; [Petty]: Government Officials
Denmark	2007	Eurobarometers	1000	1000	[Grand]: State; [Grand]: Judicial; [Grand]: Government Officials; [Grand]: Authorities; [Grand]: Local Government; [Petty]: Authorities; [Petty]: Government Officials; [Petty]: Judicial; [Petty]: Education; [Petty]: Health
Denmark	2010	Europe Quality of Government QoG	975	975	[Grand]: Authorities; [Petty]: Authorities; [Petty]: Education; [Petty]: Health; [Petty]: Election
Denmark	2011	Eurobarometers	1002	1002	[Grand]: State; [Grand]: Judicial; [Grand]: Government Officials; [Grand]: Authorities; [Grand]: Local Government; [Petty]: Authorities; [Petty]: Government Officials; [Petty]: Judicial; [Petty]: Education; [Petty]: Health
Denmark	2013	Europe Quality of Government QoG	1950	2028	[Grand]: Authorities; [Petty]: Authorities; [Petty]: Education; [Petty]: Health; [Petty]: Election
Denmark	2017	Eurobarometers	1005	1005	[Grand]: State; [Grand]: Judicial; [Grand]: Government Officials; [Grand]: Authorities; [Grand]: Local Government; [Petty]: Authorities; [Petty]: Government Officials; [Petty]: Judicial; [Petty]: Education; [Petty]: Health
Denmark	2017	Europe Quality of Government QoG	2199	2250	[Grand]: Authorities; [Petty]: Authorities; [Petty]: Education; [Petty]: Health; [Petty]: Election
Denmark	2019	Eurobarometers	1026	1026	[Grand]: State; [Grand]: Judicial; [Grand]: Government Officials; [Grand]: Authorities; [Grand]: Local Government; [Petty]: Authorities; [Petty]: Government Officials; [Petty]: Judicial; [Petty]: Education; [Petty]: Health
Denmark	2021	Europe Quality of Government QoG	2227	2536	[Grand]: Authorities; [Petty]: Authorities; [Petty]: Education; [Petty]: Health
Denmark	2022	Eurobarometers	1057	1057	[Grand]: State; [Grand]: Judicial; [Grand]: Government Officials; [Grand]: Authorities; [Grand]: Local Government; [Petty]: Authorities; [Petty]: Government Officials; [Petty]: Judicial; [Petty]: Education; [Petty]: Health
Djibouti	2012	World Bank Country Group Surveys	118	0	[Grand]: State
Djibouti	2015	World Bank Country Group Surveys	191	0	[Grand]: State
Djibouti	2018	World Bank Country Group Surveys	199	0	[Grand]: State
Dominican Republic	1996	World Value Surveys	412	0	[Grand]: Severity
Dominican Republic	2006	International Social Survey Programme	1944	2061	[Grand]: Politician; [Grand]: Government Officials; [Petty]: Government Officials
Dominican Republic	2008	LAPOP	1361	1506	[Grand]: Government Officials; [Petty]: Authorities; [Petty]: Government Officials; [Petty]: Judicial; [Petty]: Education; [Petty]: Documents; [Petty]: Health
Dominican Republic	2010	LAPOP	1402	1498	[Grand]: Government Officials; [Petty]: Authorities; [Petty]: Government Officials; [Petty]: Judicial; [Petty]: Education; [Petty]: Documents; [Petty]: Health
Dominican Republic	2012	LAPOP	1436	1512	[Grand]: Government Officials; [Petty]: Authorities; [Petty]: Government Officials; [Petty]: Judicial; [Petty]: Education; [Petty]: Documents; [Petty]: Health
Dominican Republic	2013	Latinobarometers	903	0	[Grand]: State; [Grand]: Local Government
Dominican Republic	2014	LAPOP	1473	1520	[Grand]: Government Officials; [Petty]: Authorities; [Petty]: Government Officials; [Petty]: Judicial; [Petty]: Education; [Petty]: Documents; [Petty]: Health

Dominican Republic	2016	LAPOP	1412	1517	[Grand]: Politician; [Petty]: Authorities; [Petty]: Government Officials; [Petty]: Judicial; [Petty]: Education; [Petty]: Documents; [Petty]: Health
Dominican Republic	2016	Latinobarometers	982	0	[Grand]: Executive; [Grand]: Legislative; [Grand]: Judicial; [Grand]: Taxation; [Grand]: Government Officials; [Grand]: Authorities; [Grand]: Local Government
Dominican Republic	2017	Latinobarometers	985	0	[Grand]: Legislative; [Grand]: State; [Grand]: Judicial; [Grand]: Local Government
Dominican Republic	2018	Latinobarometers	946	0	[Grand]: Executive; [Grand]: Legislative; [Grand]: Judicial; [Grand]: Taxation; [Grand]: Government Officials; [Grand]: Authorities; [Grand]: Local Government
Dominican Republic	2019	LAPOP	1440	1516	[Grand]: Politician; [Grand]: Government Officials; [Petty]: Authorities; [Petty]: Government Officials; [Petty]: Judicial; [Petty]: Education; [Petty]: Documents; [Petty]: Health
Ecuador	2000	Latinobarometers	1188	0	[Grand]: Severity
Ecuador	2001	Latinobarometers	1192	0	[Grand]: Civil; [Grand]: Severity
Ecuador	2004	LAPOP	2813	2989	[Grand]: Government Officials; [Petty]: Authorities; [Petty]: Government Officials; [Petty]: Judicial; [Petty]: Education; [Petty]: Documents; [Petty]: Health
Ecuador	2006	LAPOP	2877	2922	[Grand]: Government Officials; [Petty]: Authorities; [Petty]: Government Officials; [Petty]: Judicial; [Petty]: Education; [Petty]: Documents; [Petty]: Health
Ecuador	2008	LAPOP	2866	2995	[Grand]: Government Officials; [Petty]: Authorities; [Petty]: Government Officials; [Petty]: Judicial; [Petty]: Education; [Petty]: Documents; [Petty]: Health
Ecuador	2010	LAPOP	2895	2997	[Grand]: Government Officials; [Petty]: Authorities; [Petty]: Government Officials; [Petty]: Judicial; [Petty]: Education; [Petty]: Documents; [Petty]: Health
Ecuador	2012	LAPOP	1383	1495	[Grand]: Government Officials; [Petty]: Authorities; [Petty]: Government Officials; [Petty]: Judicial; [Petty]: Education; [Petty]: Documents; [Petty]: Health
Ecuador	2013	Latinobarometers	1151	0	[Grand]: State; [Grand]: Local Government
Ecuador	2013	World Value Surveys	0	1169	[Petty]: Election
Ecuador	2016	LAPOP	1466	1544	[Grand]: Politician; [Petty]: Authorities; [Petty]: Government Officials; [Petty]: Judicial; [Petty]: Education; [Petty]: Documents; [Petty]: Health
Ecuador	2016	Latinobarometers	1139	0	[Grand]: Executive; [Grand]: Legislative; [Grand]: Judicial; [Grand]: Taxation; [Grand]: Government Officials; [Grand]: Authorities; [Grand]: Local Government
Ecuador	2017	Latinobarometers	1194	0	[Grand]: Legislative; [Grand]: State; [Grand]: Judicial; [Grand]: Local Government
Ecuador	2018	Latinobarometers	1155	0	[Grand]: Executive; [Grand]: Legislative; [Grand]: Judicial; [Grand]: Taxation; [Grand]: Government Officials; [Grand]: Authorities; [Grand]: Local Government
Egypt	2011	Arabbarometers	1193	0	[Grand]: State
Egypt	2012	Afrobarometers	1127	1174	[Grand]: Executive; [Grand]: Legislative; [Grand]: Judicial; [Grand]: Taxation; [Grand]: Government Officials; [Grand]: Authorities; [Petty]: Authorities; [Petty]: Utilities; [Petty]: Education; [Petty]: Documents; [Petty]: Health; [Petty]: Election
Egypt	2013	World Value Surveys	0	1400	[Petty]: Election
Egypt	2014	Arabbarometers	1110	0	[Grand]: State
Egypt	2015	Afrobarometers	1128	1154	[Grand]: Taxation; [Grand]: Government Officials; [Grand]: Authorities; [Grand]: Local Government; [Petty]: Authorities; [Petty]: Utilities; [Petty]: Education; [Petty]: Documents; [Petty]: Health; [Petty]: Election
Egypt	2017	Arabbarometers	1146	0	[Grand]: State
Egypt	2018	World Value Surveys	1164	930	[Grand]: Severity; [Petty]: Election
El Salvador	1998	Latinobarometers	997	0	[Grand]: Severity
El Salvador	1999	World Value Surveys	1208	0	[Grand]: Severity
El Salvador	2000	Latinobarometers	987	0	[Grand]: Severity
El Salvador	2001	Latinobarometers	992	0	[Grand]: Civil; [Grand]: Severity
El Salvador	2004	LAPOP	1502	1588	[Grand]: Government Officials; [Petty]: Authorities; [Petty]: Government Officials; [Petty]: Judicial; [Petty]: Education; [Petty]: Documents; [Petty]: Health

El Salvador	2006	LAPOP	1662	1729	[Grand]: Government Officials; [Petty]: Authorities; [Petty]: Government Officials; [Petty]: Judicial; [Petty]: Education; [Petty]: Documents; [Petty]: Health
El Salvador	2008	LAPOP	1523	1549	[Grand]: Government Officials; [Petty]: Authorities; [Petty]: Government Officials; [Petty]: Judicial; [Petty]: Education; [Petty]: Documents; [Petty]: Health
El Salvador	2010	LAPOP	1525	1550	[Grand]: Government Officials; [Petty]: Authorities; [Petty]: Government Officials; [Petty]: Judicial; [Petty]: Education; [Petty]: Documents; [Petty]: Health
El Salvador	2012	LAPOP	1381	1496	[Grand]: Government Officials; [Petty]: Authorities; [Petty]: Government Officials; [Petty]: Judicial; [Petty]: Education; [Petty]: Documents; [Petty]: Health
El Salvador	2013	Latinobarometers	928	0	[Grand]: State; [Grand]: Local Government
El Salvador	2014	LAPOP	1482	1512	[Grand]: Government Officials; [Petty]: Authorities; [Petty]: Government Officials; [Petty]: Judicial; [Petty]: Education; [Petty]: Documents; [Petty]: Health
El Salvador	2016	Latinobarometers	869	0	[Grand]: Executive; [Grand]: Legislative; [Grand]: Judicial; [Grand]: Taxation; [Grand]: Government Officials; [Grand]: Authorities; [Grand]: Local Government
El Salvador	2016	LAPOP	1543	1551	[Grand]: Politician; [Grand]: Government Officials; [Petty]: Authorities; [Petty]: Government Officials; [Petty]: Judicial; [Petty]: Education; [Petty]: Documents; [Petty]: Health
El Salvador	2017	Latinobarometers	968	0	[Grand]: Legislative; [Grand]: State; [Grand]: Judicial; [Grand]: Local Government
El Salvador	2018	Latinobarometers	893	0	[Grand]: Executive; [Grand]: Legislative; [Grand]: Judicial; [Grand]: Taxation; [Grand]: Government Officials; [Grand]: Authorities; [Grand]: Local Government
El Salvador	2018	LAPOP	1440	1511	[Grand]: Politician; [Grand]: Government Officials; [Petty]: Authorities; [Petty]: Government Officials; [Petty]: Judicial; [Petty]: Education; [Petty]: Documents; [Petty]: Health
Equatorial Guinea	2017	World Bank Country Group Surveys	158	0	[Grand]: State
Equatorial Guinea	2020	World Bank Country Group Surveys	153	0	[Grand]: State
Estonia	1996	World Value Surveys	911	0	[Grand]: Severity
Estonia	2007	Eurobarometers	1002	1002	[Grand]: State; [Grand]: Judicial; [Grand]: Government Officials; [Grand]: Authorities; [Grand]: Local Government; [Petty]: Authorities; [Petty]: Government Officials; [Petty]: Judicial; [Petty]: Education; [Petty]: Health
Estonia	2011	World Value Surveys	0	1272	[Petty]: Election
Estonia	2011	Eurobarometers	1000	1000	[Grand]: State; [Grand]: Judicial; [Grand]: Government Officials; [Grand]: Authorities; [Grand]: Local Government; [Petty]: Authorities; [Petty]: Government Officials; [Petty]: Judicial; [Petty]: Education; [Petty]: Health
Estonia	2017	Eurobarometers	1004	1004	[Grand]: State; [Grand]: Judicial; [Grand]: Taxation; [Grand]: Government Officials; [Grand]: Authorities; [Grand]: Local Government; [Petty]: Authorities; [Petty]: Government Officials; [Petty]: Judicial; [Petty]: Education; [Petty]: Health
Estonia	2019	Eurobarometers	1001	1001	[Grand]: State; [Grand]: Judicial; [Grand]: Taxation; [Grand]: Government Officials; [Grand]: Authorities; [Grand]: Local Government; [Petty]: Authorities; [Petty]: Government Officials; [Petty]: Judicial; [Petty]: Education; [Petty]: Health
Estonia	2021	Europe Quality of Government QoG	860	1058	[Grand]: Authorities; [Petty]: Authorities; [Petty]: Education; [Petty]: Health
Estonia	2022	Eurobarometers	1007	1007	[Grand]: State; [Grand]: Judicial; [Grand]: Taxation; [Grand]: Government Officials; [Grand]: Authorities; [Grand]: Local Government; [Petty]: Authorities; [Petty]: Government Officials; [Petty]: Judicial; [Petty]: Education; [Petty]: Health
Eswatini	2012	Afrobarometers	1097	1167	[Grand]: Executive; [Grand]: Legislative; [Grand]: Judicial; [Grand]: Taxation; [Grand]: Government Officials; [Grand]: Authorities; [Grand]: Local Government; [Petty]: Authorities; [Petty]: Utilities; [Petty]: Education; [Petty]: Documents; [Petty]: Health; [Petty]: Election
Eswatini	2015	Afrobarometers	1137	1185	[Grand]: Executive; [Grand]: Legislative; [Grand]: Judicial; [Grand]: Taxation; [Grand]: Government Officials; [Grand]: Authorities; [Grand]: Local Government; [Petty]: Authorities; [Petty]: Utilities; [Petty]: Education; [Petty]: Documents; [Petty]: Health; [Petty]: Election

Eswatini	2018	Afrobarometers	1028	1200	[Grand]: Executive; [Grand]: Legislative; [Grand]: Judicial; [Grand]: Government Officials; [Grand]: Authorities; [Grand]: Local Government; [Petty]: Authorities; [Petty]: Education; [Petty]: Documents; [Petty]: Health
Eswatini	2021	Afrobarometers	1142	1084	[Grand]: Executive; [Grand]: Legislative; [Grand]: Judicial; [Grand]: Taxation; [Grand]: Civil; [Grand]: Authorities; [Grand]: Local Government; [Petty]: Authorities; [Petty]: Education; [Petty]: Documents; [Petty]: Health
Ethiopia	2020	World Value Surveys	1203	1204	[Grand]: State; [Grand]: Civil; [Grand]: Severity; [Grand]: Local Government; [Petty]: Government Officials; [Petty]: Election
Ethiopia	2020	Afrobarometers	2300	1892	[Grand]: Executive; [Grand]: Legislative; [Grand]: Judicial; [Grand]: Taxation; [Grand]: Civil; [Grand]: Authorities; [Grand]: Local Government; [Petty]: Authorities; [Petty]: Education; [Petty]: Documents; [Petty]: Health
FSM	2017	Transparency International	503	499	[Grand]: Executive; [Grand]: Legislative; [Grand]: State; [Grand]: Judicial; [Grand]: Civil; [Grand]: Authorities; [Grand]: Local Government; [Petty]: Authorities; [Petty]: Judicial; [Petty]: Utilities; [Petty]: Education; [Petty]: Documents; [Petty]: Health; [Petty]: Election
Fiji	2017	Transparency International	989	1016	[Grand]: Executive; [Grand]: Legislative; [Grand]: State; [Grand]: Judicial; [Grand]: Civil; [Grand]: Authorities; [Grand]: Local Government; [Petty]: Authorities; [Petty]: Judicial; [Petty]: Utilities; [Petty]: Education; [Petty]: Documents; [Petty]: Health; [Petty]: Election
Finland	2006	International Social Survey Programme	980	1110	[Grand]: Politician; [Grand]: Government Officials; [Petty]: Government Officials
Finland	2007	Eurobarometers	1038	1038	[Grand]: State; [Grand]: Judicial; [Grand]: Government Officials; [Grand]: Authorities; [Grand]: Local Government; [Petty]: Authorities; [Petty]: Government Officials; [Petty]: Judicial; [Petty]: Education; [Petty]: Health
Finland	2011	Eurobarometers	1003	1003	[Grand]: State; [Grand]: Judicial; [Grand]: Government Officials; [Grand]: Authorities; [Grand]: Local Government; [Petty]: Authorities; [Petty]: Government Officials; [Petty]: Judicial; [Petty]: Education; [Petty]: Health
Finland	2017	Eurobarometers	1017	1017	[Grand]: State; [Grand]: Judicial; [Grand]: Government Officials; [Grand]: Authorities; [Grand]: Local Government; [Petty]: Authorities; [Petty]: Government Officials; [Petty]: Judicial; [Petty]: Education; [Petty]: Health
Finland	2017	Europe Quality of Government QoG	1947	2000	[Grand]: Authorities; [Petty]: Authorities; [Petty]: Education; [Petty]: Health; [Petty]: Election
Finland	2019	Eurobarometers	1007	1007	[Grand]: State; [Grand]: Judicial; [Grand]: Government Officials; [Grand]: Authorities; [Grand]: Local Government; [Petty]: Authorities; [Petty]: Government Officials; [Petty]: Judicial; [Petty]: Education; [Petty]: Health
Finland	2021	Europe Quality of Government QoG	2285	2484	[Grand]: Authorities; [Petty]: Authorities; [Petty]: Education; [Petty]: Health
Finland	2022	Eurobarometers	1008	1008	[Grand]: State; [Grand]: Judicial; [Grand]: Government Officials; [Grand]: Authorities; [Grand]: Local Government; [Petty]: Authorities; [Petty]: Government Officials; [Petty]: Judicial; [Petty]: Education; [Petty]: Health
France	2006	International Social Survey Programme	1732	1713	[Grand]: Politician; [Grand]: Government Officials; [Petty]: Government Officials
France	2007	Eurobarometers	1024	1024	[Grand]: State; [Grand]: Judicial; [Grand]: Government Officials; [Grand]: Authorities; [Grand]: Local Government; [Petty]: Authorities; [Petty]: Government Officials; [Petty]: Judicial; [Petty]: Education; [Petty]: Health
France	2010	Europe Quality of Government QoG	5070	5070	[Grand]: Authorities; [Petty]: Authorities; [Petty]: Education; [Petty]: Health; [Petty]: Election
France	2011	Eurobarometers	1046	1046	[Grand]: State; [Grand]: Judicial; [Grand]: Government Officials; [Grand]: Authorities; [Grand]: Local Government; [Petty]: Authorities; [Petty]: Government Officials; [Petty]: Judicial; [Petty]: Education; [Petty]: Health
France	2013	Europe Quality of Government QoG	10315	10409	[Grand]: Authorities; [Petty]: Authorities; [Petty]: Education; [Petty]: Health; [Petty]: Election
France	2017	Eurobarometers	1030	1030	[Grand]: State; [Grand]: Judicial; [Grand]: Taxation; [Grand]: Government Officials; [Grand]: Authorities; [Grand]: Local Government; [Petty]: Authorities; [Petty]: Government Officials; [Petty]: Judicial; [Petty]: Education; [Petty]: Health
France	2017	Europe Quality of Government QoG	10352	10422	[Grand]: Authorities; [Petty]: Authorities; [Petty]: Education; [Petty]: Health; [Petty]: Election
France	2019	Eurobarometers	1026	1026	[Grand]: State; [Grand]: Judicial; [Grand]: Taxation; [Grand]: Government Officials; [Grand]: Authorities; [Grand]: Local Government; [Petty]: Authorities; [Petty]: Government Officials; [Petty]: Judicial; [Petty]: Education; [Petty]: Health
France	2021	Europe Quality of Government QoG	11748	13111	[Grand]: Authorities; [Petty]: Authorities; [Petty]: Education; [Petty]: Health

France	2022	Eurobarometers	1033	1033	[Grand]: State; [Grand]: Judicial; [Grand]: Taxation; [Grand]: Government Officials; [Grand]: Authorities; [Grand]: Local Government; [Petty]: Authorities; [Petty]: Government Officials; [Petty]: Judicial; [Petty]: Education; [Petty]: Health
French Polynesia	2017	Transparency International	508	510	[Grand]: Executive; [Grand]: Legislative; [Grand]: State; [Grand]: Judicial; [Grand]: Civil; [Grand]: Authorities; [Grand]: Local Government; [Petty]: Authorities; [Petty]: Judicial; [Petty]: Utilities; [Petty]: Education; [Petty]: Documents; [Petty]: Health; [Petty]: Election
Gabon	2015	Afrobarometers	1189	1193	[Grand]: Executive; [Grand]: Legislative; [Grand]: Judicial; [Grand]: Taxation; [Grand]: Government Officials; [Grand]: Authorities; [Grand]: Local Government; [Petty]: Authorities; [Petty]: Utilities; [Petty]: Education; [Petty]: Documents; [Petty]: Health; [Petty]: Election
Gabon	2017	Afrobarometers	1179	1199	[Grand]: Executive; [Grand]: Legislative; [Grand]: Judicial; [Grand]: Government Officials; [Grand]: Authorities; [Grand]: Local Government; [Petty]: Authorities; [Petty]: Education; [Petty]: Documents; [Petty]: Health
Gabon	2020	Afrobarometers	1198	1007	[Grand]: Executive; [Grand]: Legislative; [Grand]: Judicial; [Grand]: Taxation; [Grand]: Civil; [Grand]: Authorities; [Grand]: Local Government; [Petty]: Authorities; [Petty]: Education; [Petty]: Documents; [Petty]: Health
Gambia	2018	Afrobarometers	1114	1200	[Grand]: Executive; [Grand]: Legislative; [Grand]: Judicial; [Grand]: Government Officials; [Grand]: Authorities; [Grand]: Local Government; [Petty]: Authorities; [Petty]: Education; [Petty]: Documents; [Petty]: Health
Gambia	2021	Afrobarometers	1113	1071	[Grand]: Executive; [Grand]: Legislative; [Grand]: Judicial; [Grand]: Taxation; [Grand]: Civil; [Grand]: Authorities; [Grand]: Local Government; [Petty]: Authorities; [Petty]: Education; [Petty]: Documents; [Petty]: Health
Georgia	1996	World Value Surveys	1850	0	[Grand]: Severity
Georgia	2014	World Value Surveys	0	951	[Petty]: Election
Germany	1997	World Value Surveys	1952	0	[Grand]: Severity
Germany	2006	International Social Survey Programme	1493	1511	[Grand]: Politician; [Grand]: Government Officials; [Petty]: Government Officials
Germany	2007	Eurobarometers	1519	1519	[Grand]: State; [Grand]: Judicial; [Grand]: Government Officials; [Grand]: Authorities; [Grand]: Local Government; [Petty]: Authorities; [Petty]: Government Officials; [Petty]: Judicial; [Petty]: Education; [Petty]: Health
Germany	2010	Europe Quality of Government QoG	3120	3120	[Grand]: Authorities; [Petty]: Authorities; [Petty]: Education; [Petty]: Health; [Petty]: Election
Germany	2011	Eurobarometers	1582	1582	[Grand]: State; [Grand]: Judicial; [Grand]: Government Officials; [Grand]: Authorities; [Grand]: Local Government; [Petty]: Authorities; [Petty]: Government Officials; [Petty]: Judicial; [Petty]: Education; [Petty]: Health
Germany	2013	World Value Surveys	0	1864	[Petty]: Election
Germany	2013	Europe Quality of Government QoG	6382	6400	[Grand]: Authorities; [Petty]: Authorities; [Petty]: Education; [Petty]: Health; [Petty]: Election
Germany	2017	Eurobarometers	1554	1554	[Grand]: State; [Grand]: Judicial; [Grand]: Taxation; [Grand]: Government Officials; [Grand]: Authorities; [Grand]: Local Government; [Petty]: Authorities; [Petty]: Government Officials; [Petty]: Judicial; [Petty]: Education; [Petty]: Health
Germany	2017	Europe Quality of Government QoG	7159	7200	[Grand]: Authorities; [Petty]: Authorities; [Petty]: Education; [Petty]: Health; [Petty]: Election
Germany	2018	World Value Surveys	1506	1518	[Grand]: State; [Grand]: Civil; [Grand]: Severity; [Grand]: Local Government; [Petty]: Government Officials; [Petty]: Election
Germany	2019	Eurobarometers	1526	1526	[Grand]: State; [Grand]: Judicial; [Grand]: Taxation; [Grand]: Government Officials; [Grand]: Authorities; [Grand]: Local Government; [Petty]: Authorities; [Petty]: Government Officials; [Petty]: Judicial; [Petty]: Education; [Petty]: Health
Germany	2021	Europe Quality of Government QoG	16741	19182	[Grand]: Authorities; [Petty]: Authorities; [Petty]: Education; [Petty]: Health
Germany	2022	Eurobarometers	1519	1519	[Grand]: State; [Grand]: Judicial; [Grand]: Taxation; [Grand]: Government Officials; [Grand]: Authorities; [Grand]: Local Government; [Petty]: Authorities; [Petty]: Government Officials; [Petty]: Judicial; [Petty]: Education; [Petty]: Health
Ghana	2008	Afrobarometers	1126	1032	[Grand]: Executive; [Grand]: Legislative; [Grand]: Judicial; [Grand]: Taxation; [Grand]: Government Officials; [Grand]: Authorities; [Grand]: Local Government; [Petty]: Authorities; [Petty]: Utilities; [Petty]: Documents

Ghana	2012	Afrobarometers	2364	2390	[Grand]: Executive; [Grand]: Legislative; [Grand]: Judicial; [Grand]: Taxation; [Grand]: Government Officials; [Grand]: Authorities; [Grand]: Local Government; [Petty]: Authorities; [Petty]: Utilities; [Petty]: Education; [Petty]: Documents; [Petty]: Health; [Petty]: Election
Ghana	2012	World Value Surveys	0	1480	[Petty]: Election
Ghana	2014	Afrobarometers	2282	2248	[Grand]: Executive; [Grand]: Legislative; [Grand]: Judicial; [Grand]: Taxation; [Grand]: Government Officials; [Grand]: Authorities; [Grand]: Local Government; [Petty]: Authorities; [Petty]: Utilities; [Petty]: Education; [Petty]: Documents; [Petty]: Health; [Petty]: Election
Ghana	2017	Afrobarometers	2281	2400	[Grand]: Executive; [Grand]: Legislative; [Grand]: Judicial; [Grand]: Government Officials; [Grand]: Authorities; [Grand]: Local Government; [Petty]: Authorities; [Petty]: Education; [Petty]: Documents; [Petty]: Health
Greece	2007	Eurobarometers	1000	1000	[Grand]: State; [Grand]: Judicial; [Grand]: Government Officials; [Grand]: Authorities; [Grand]: Local Government; [Petty]: Authorities; [Petty]: Government Officials; [Petty]: Judicial; [Petty]: Education; [Petty]: Health
Greece	2011	Eurobarometers	1000	1000	[Grand]: State; [Grand]: Judicial; [Grand]: Government Officials; [Grand]: Authorities; [Grand]: Local Government; [Petty]: Authorities; [Petty]: Government Officials; [Petty]: Judicial; [Petty]: Education; [Petty]: Health
Greece	2017	Eurobarometers	1003	1003	[Grand]: State; [Grand]: Judicial; [Grand]: Taxation; [Grand]: Government Officials; [Grand]: Authorities; [Grand]: Local Government; [Petty]: Authorities; [Petty]: Government Officials; [Petty]: Judicial; [Petty]: Education; [Petty]: Health
Greece	2017	World Value Surveys	1196	1181	[Grand]: State; [Grand]: Civil; [Grand]: Severity; [Grand]: Local Government; [Petty]: Government Officials; [Petty]: Election
Greece	2017	Europe Quality of Government QoG	1547	1620	[Grand]: Authorities; [Petty]: Authorities; [Petty]: Education; [Petty]: Health; [Petty]: Election
Greece	2019	Eurobarometers	1008	1008	[Grand]: State; [Grand]: Judicial; [Grand]: Taxation; [Grand]: Government Officials; [Grand]: Authorities; [Grand]: Local Government; [Petty]: Authorities; [Petty]: Government Officials; [Petty]: Judicial; [Petty]: Education; [Petty]: Health
Greece	2021	Europe Quality of Government QoG	6098	6780	[Grand]: Authorities; [Petty]: Authorities; [Petty]: Education; [Petty]: Health
Greece	2022	Eurobarometers	1013	1013	[Grand]: State; [Grand]: Judicial; [Grand]: Taxation; [Grand]: Government Officials; [Grand]: Authorities; [Grand]: Local Government; [Petty]: Authorities; [Petty]: Government Officials; [Petty]: Judicial; [Petty]: Education; [Petty]: Health
Guatemala	2000	Latinobarometers	972	0	[Grand]: Severity
Guatemala	2001	Latinobarometers	977	0	[Grand]: Civil; [Grand]: Severity
Guatemala	2004	LAPOP	1435	1708	[Grand]: Government Officials; [Petty]: Authorities; [Petty]: Government Officials; [Petty]: Judicial; [Petty]: Education; [Petty]: Documents; [Petty]: Health
Guatemala	2006	LAPOP	1263	1493	[Grand]: Government Officials; [Petty]: Authorities; [Petty]: Government Officials; [Petty]: Judicial; [Petty]: Education; [Petty]: Documents; [Petty]: Health
Guatemala	2008	LAPOP	1375	1535	[Grand]: Government Officials; [Petty]: Authorities; [Petty]: Government Officials; [Petty]: Judicial; [Petty]: Education; [Petty]: Documents; [Petty]: Health
Guatemala	2010	LAPOP	1355	1484	[Grand]: Government Officials; [Petty]: Authorities; [Petty]: Government Officials; [Petty]: Judicial; [Petty]: Education; [Petty]: Documents; [Petty]: Health
Guatemala	2012	LAPOP	1314	1507	[Grand]: Government Officials; [Petty]: Authorities; [Petty]: Government Officials; [Petty]: Judicial; [Petty]: Education; [Petty]: Documents; [Petty]: Health
Guatemala	2013	Latinobarometers	926	0	[Grand]: State; [Grand]: Local Government
Guatemala	2014	LAPOP	1408	1505	[Grand]: Government Officials; [Petty]: Authorities; [Petty]: Government Officials; [Petty]: Judicial; [Petty]: Education; [Petty]: Documents; [Petty]: Health
Guatemala	2016	Latinobarometers	932	0	[Grand]: Executive; [Grand]: Legislative; [Grand]: Judicial; [Grand]: Taxation; [Grand]: Government Officials; [Grand]: Authorities; [Grand]: Local Government
Guatemala	2017	LAPOP	1480	1543	[Grand]: Politician; [Petty]: Authorities; [Petty]: Government Officials; [Petty]: Judicial; [Petty]: Education; [Petty]: Documents; [Petty]: Health
Guatemala	2017	Latinobarometers	966	0	[Grand]: Legislative; [Grand]: State; [Grand]: Judicial; [Grand]: Local Government

Guatemala	2018	Latinobarometers	859	0	[Grand]: Executive; [Grand]: Legislative; [Grand]: Judicial; [Grand]: Taxation; [Grand]: Government Officials; [Grand]: Authorities; [Grand]: Local Government
Guatemala	2019	LAPOP	1517	1596	[Grand]: Politician; [Grand]: Government Officials; [Petty]: Authorities; [Petty]: Government Officials; [Petty]: Judicial; [Petty]: Education; [Petty]: Documents; [Petty]: Health
Guatemala	2020	World Value Surveys	1203	1203	[Grand]: State; [Grand]: Civil; [Grand]: Severity; [Grand]: Local Government; [Petty]: Government Officials; [Petty]: Election
Guinea	2012	Afrobarometers	1151	1199	[Grand]: Executive; [Grand]: Legislative; [Grand]: Judicial; [Grand]: Taxation; [Grand]: Government Officials; [Grand]: Authorities; [Grand]: Local Government; [Petty]: Authorities; [Petty]: Utilities; [Petty]: Education; [Petty]: Documents; [Petty]: Health; [Petty]: Election
Guinea	2015	Afrobarometers	1155	1165	[Grand]: Executive; [Grand]: Legislative; [Grand]: Judicial; [Grand]: Taxation; [Grand]: Government Officials; [Grand]: Authorities; [Grand]: Local Government; [Petty]: Authorities; [Petty]: Utilities; [Petty]: Education; [Petty]: Documents; [Petty]: Health; [Petty]: Election
Guinea	2017	Afrobarometers	1170	1194	[Grand]: Executive; [Grand]: Legislative; [Grand]: Judicial; [Grand]: Government Officials; [Grand]: Authorities; [Grand]: Local Government; [Petty]: Authorities; [Petty]: Education; [Petty]: Documents; [Petty]: Health
Guinea	2019	Afrobarometers	1194	1060	[Grand]: Executive; [Grand]: Legislative; [Grand]: Judicial; [Grand]: Taxation; [Grand]: Civil; [Grand]: Authorities; [Grand]: Local Government; [Petty]: Authorities; [Petty]: Education; [Petty]: Documents; [Petty]: Health
Guinea Bissau	2014	World Bank Country Group Surveys	169	0	[Grand]: State
Guinea Bissau	2021	World Bank Country Group Surveys	193	0	[Grand]: State
Guyana	2006	LAPOP	1413	1555	[Grand]: Government Officials; [Petty]: Authorities; [Petty]: Government Officials; [Petty]: Judicial; [Petty]: Education; [Petty]: Documents; [Petty]: Health
Guyana	2009	LAPOP	2392	2507	[Grand]: Government Officials; [Petty]: Authorities; [Petty]: Government Officials; [Petty]: Judicial; [Petty]: Education; [Petty]: Documents; [Petty]: Health
Guyana	2010	LAPOP	1490	1540	[Grand]: Government Officials; [Petty]: Authorities; [Petty]: Government Officials; [Petty]: Judicial; [Petty]: Education; [Petty]: Documents; [Petty]: Health
Guyana	2012	LAPOP	1461	1529	[Grand]: Government Officials; [Petty]: Authorities; [Petty]: Government Officials; [Petty]: Judicial; [Petty]: Education; [Petty]: Documents; [Petty]: Health
Guyana	2014	LAPOP	1535	1553	[Grand]: Government Officials; [Petty]: Authorities; [Petty]: Government Officials; [Petty]: Judicial; [Petty]: Education; [Petty]: Documents; [Petty]: Health
Haiti	2006	LAPOP	1586	1615	[Grand]: Government Officials; [Petty]: Authorities; [Petty]: Government Officials; [Petty]: Judicial; [Petty]: Education; [Petty]: Documents; [Petty]: Health
Haiti	2008	LAPOP	1503	1515	[Grand]: Government Officials; [Petty]: Authorities; [Petty]: Government Officials; [Petty]: Judicial; [Petty]: Education; [Petty]: Documents; [Petty]: Health
Haiti	2010	LAPOP	1626	1742	[Grand]: Government Officials; [Petty]: Authorities; [Petty]: Government Officials; [Petty]: Judicial; [Petty]: Education; [Petty]: Documents; [Petty]: Health
Haiti	2012	LAPOP	1702	1823	[Grand]: Government Officials; [Petty]: Authorities; [Petty]: Government Officials; [Petty]: Judicial; [Petty]: Education; [Petty]: Documents; [Petty]: Health
Haiti	2014	LAPOP	1220	1507	[Grand]: Government Officials; [Petty]: Authorities; [Petty]: Government Officials; [Petty]: Judicial; [Petty]: Education; [Petty]: Documents; [Petty]: Health
Haiti	2017	LAPOP	1556	2205	[Grand]: Politician; [Petty]: Authorities; [Petty]: Government Officials
Honduras	2000	Latinobarometers	994	0	[Grand]: Severity
Honduras	2001	Latinobarometers	998	0	[Grand]: Civil; [Grand]: Severity
Honduras	2004	LAPOP	1139	1380	[Grand]: Government Officials; [Petty]: Authorities; [Petty]: Government Officials; [Petty]: Judicial; [Petty]: Education; [Petty]: Documents; [Petty]: Health
Honduras	2006	LAPOP	1431	1519	[Grand]: Government Officials; [Petty]: Authorities; [Petty]: Government Officials; [Petty]: Judicial; [Petty]: Education; [Petty]: Documents; [Petty]: Health

Honduras	2008	LAPOP	1378	1457	[Grand]: Government Officials; [Petty]: Authorities; [Petty]: Government Officials; [Petty]: Judicial; [Petty]: Education; [Petty]: Documents; [Petty]: Health
Honduras	2010	LAPOP	1457	1530	[Grand]: Government Officials; [Petty]: Authorities; [Petty]: Government Officials; [Petty]: Judicial; [Petty]: Education; [Petty]: Documents; [Petty]: Health
Honduras	2012	LAPOP	1554	1725	[Grand]: Government Officials; [Petty]: Authorities; [Petty]: Government Officials; [Petty]: Judicial; [Petty]: Education; [Petty]: Documents; [Petty]: Health
Honduras	2013	Latinobarometers	951	0	[Grand]: State; [Grand]: Local Government
Honduras	2014	LAPOP	1511	1561	[Grand]: Government Officials; [Petty]: Authorities; [Petty]: Government Officials; [Petty]: Judicial; [Petty]: Education; [Petty]: Documents; [Petty]: Health
Honduras	2016	Latinobarometers	961	0	[Grand]: Executive; [Grand]: Legislative; [Grand]: Judicial; [Grand]: Taxation; [Grand]: Government Officials; [Grand]: Authorities; [Grand]: Local Government
Honduras	2016	LAPOP	1442	1560	[Grand]: Politician; [Petty]: Authorities; [Petty]: Government Officials; [Petty]: Judicial; [Petty]: Education; [Petty]: Documents; [Petty]: Health
Honduras	2017	Latinobarometers	986	0	[Grand]: Legislative; [Grand]: State; [Grand]: Judicial; [Grand]: Local Government
Honduras	2018	Latinobarometers	939	0	[Grand]: Executive; [Grand]: Legislative; [Grand]: Judicial; [Grand]: Taxation; [Grand]: Government Officials; [Grand]: Authorities; [Grand]: Local Government
Honduras	2018	LAPOP	1473	1559	[Grand]: Politician; [Grand]: Government Officials; [Petty]: Authorities; [Petty]: Government Officials; [Petty]: Judicial; [Petty]: Education; [Petty]: Documents; [Petty]: Health
Hungary	1998	World Value Surveys	615	0	[Grand]: Severity
Hungary	2006	International Social Survey Programme	898	990	[Grand]: Politician; [Grand]: Government Officials; [Petty]: Government Officials
Hungary	2007	Eurobarometers	1000	1000	[Grand]: State; [Grand]: Judicial; [Grand]: Government Officials; [Grand]: Authorities; [Grand]: Local Government; [Petty]: Authorities; [Petty]: Government Officials; [Petty]: Judicial; [Petty]: Education; [Petty]: Health
Hungary	2011	Eurobarometers	1015	1015	[Grand]: State; [Grand]: Judicial; [Grand]: Government Officials; [Grand]: Authorities; [Grand]: Local Government; [Petty]: Authorities; [Petty]: Government Officials; [Petty]: Judicial; [Petty]: Education; [Petty]: Health
Hungary	2017	Europe Quality of Government QoG	2727	2800	[Grand]: Authorities; [Petty]: Authorities; [Petty]: Education; [Petty]: Health; [Petty]: Election
Hungary	2017	Eurobarometers	1038	1038	[Grand]: State; [Grand]: Judicial; [Grand]: Taxation; [Grand]: Government Officials; [Grand]: Authorities; [Grand]: Local Government; [Petty]: Authorities; [Petty]: Government Officials; [Petty]: Judicial; [Petty]: Education; [Petty]: Health
Hungary	2019	Eurobarometers	1026	1026	[Grand]: State; [Grand]: Judicial; [Grand]: Taxation; [Grand]: Government Officials; [Grand]: Authorities; [Grand]: Local Government; [Petty]: Authorities; [Petty]: Government Officials; [Petty]: Judicial; [Petty]: Education; [Petty]: Health
Hungary	2021	Europe Quality of Government QoG	3613	4044	[Grand]: Authorities; [Petty]: Authorities; [Petty]: Education; [Petty]: Health
Hungary	2022	Eurobarometers	1016	1016	[Grand]: State; [Grand]: Judicial; [Grand]: Taxation; [Grand]: Government Officials; [Grand]: Authorities; [Grand]: Local Government; [Petty]: Authorities; [Petty]: Government Officials; [Petty]: Judicial; [Petty]: Education; [Petty]: Health
India	1995	World Value Surveys	1742	0	[Grand]: Severity
India	2019	Asiabarometers	4665	0	[Grand]: State; [Grand]: Local Government
Indonesia	2016	Asiabarometers	1267	0	[Grand]: State; [Grand]: Local Government
Indonesia	2018	World Value Surveys	3184	3190	[Grand]: State; [Grand]: Civil; [Grand]: Severity; [Grand]: Local Government; [Petty]: Government Officials; [Petty]: Election
Indonesia	2019	Asiabarometers	1136	0	[Grand]: State; [Grand]: Local Government
Iran	2020	World Value Surveys	1494	1488	[Grand]: State; [Grand]: Civil; [Grand]: Severity; [Grand]: Local Government; [Petty]: Government Officials; [Petty]: Election
Iraq	2011	Arabbarometers	1215	0	[Grand]: State
Iraq	2012	World Value Surveys	982	1086	[Grand]: State; [Petty]: Election
Iraq	2014	Arabbarometers	2141	0	[Grand]: State

Iraq	2018	World Value Surveys	1200	1183	[Grand]: State; [Grand]: Civil; [Grand]: Severity; [Grand]: Local Government; [Petty]: Government Officials; [Petty]: Election
Ireland	2006	International Social Survey Programme	948	993	[Grand]: Politician; [Grand]: Government Officials; [Petty]: Government Officials
Ireland	2017	Eurobarometers	1008	1008	[Grand]: State; [Grand]: Judicial; [Grand]: Government Officials; [Grand]: Authorities; [Grand]: Local Government; [Petty]: Authorities; [Petty]: Government Officials; [Petty]: Judicial; [Petty]: Education; [Petty]: Health
Ireland	2019	Eurobarometers	1017	1017	[Grand]: State; [Grand]: Judicial; [Grand]: Government Officials; [Grand]: Authorities; [Grand]: Local Government; [Petty]: Authorities; [Petty]: Government Officials; [Petty]: Judicial; [Petty]: Education; [Petty]: Health
Ireland	2021	Europe Quality of Government QoG	1369	1497	[Grand]: Authorities; [Petty]: Authorities; [Petty]: Education; [Petty]: Health
Ireland	2022	Eurobarometers	1011	1011	[Grand]: State; [Grand]: Judicial; [Grand]: Government Officials; [Grand]: Authorities; [Grand]: Local Government; [Petty]: Authorities; [Petty]: Government Officials; [Petty]: Judicial; [Petty]: Education; [Petty]: Health
Israel	2006	International Social Survey Programme	1296	1292	[Grand]: Politician; [Grand]: Government Officials; [Petty]: Government Officials
Italy	2007	Eurobarometers	1039	1039	[Grand]: State; [Grand]: Judicial; [Grand]: Government Officials; [Grand]: Authorities; [Grand]: Local Government; [Petty]: Authorities; [Petty]: Government Officials; [Petty]: Judicial; [Petty]: Education; [Petty]: Health
Italy	2010	Europe Quality of Government QoG	4095	4095	[Grand]: Authorities; [Petty]: Authorities; [Petty]: Education; [Petty]: Health; [Petty]: Election
Italy	2011	Eurobarometers	1043	1043	[Grand]: State; [Grand]: Judicial; [Grand]: Government Officials; [Grand]: Authorities; [Grand]: Local Government; [Petty]: Authorities; [Petty]: Government Officials; [Petty]: Judicial; [Petty]: Education; [Petty]: Health
Italy	2013	Europe Quality of Government QoG	8425	8424	[Grand]: Authorities; [Petty]: Authorities; [Petty]: Education; [Petty]: Health; [Petty]: Election
Italy	2017	Europe Quality of Government QoG	8283	8400	[Grand]: Authorities; [Petty]: Authorities; [Petty]: Education; [Petty]: Health; [Petty]: Election
Italy	2017	Eurobarometers	1025	1025	[Grand]: State; [Grand]: Judicial; [Grand]: Taxation; [Grand]: Government Officials; [Grand]: Authorities; [Grand]: Local Government; [Petty]: Authorities; [Petty]: Government Officials; [Petty]: Judicial; [Petty]: Education; [Petty]: Health
Italy	2019	Eurobarometers	1020	1020	[Grand]: State; [Grand]: Judicial; [Grand]: Taxation; [Grand]: Government Officials; [Grand]: Authorities; [Grand]: Local Government; [Petty]: Authorities; [Petty]: Government Officials; [Petty]: Judicial; [Petty]: Education; [Petty]: Health
Italy	2021	Europe Quality of Government QoG	11848	12831	[Grand]: Authorities; [Petty]: Authorities; [Petty]: Education; [Petty]: Health
Italy	2022	Eurobarometers	1017	1017	[Grand]: State; [Grand]: Judicial; [Grand]: Taxation; [Grand]: Government Officials; [Grand]: Authorities; [Grand]: Local Government; [Petty]: Authorities; [Petty]: Government Officials; [Petty]: Judicial; [Petty]: Education; [Petty]: Health
Jamaica	2006	LAPOP	1484	1575	[Grand]: Government Officials; [Petty]: Authorities; [Petty]: Government Officials; [Petty]: Judicial; [Petty]: Education; [Petty]: Documents; [Petty]: Health
Jamaica	2008	LAPOP	1345	1497	[Grand]: Government Officials; [Petty]: Authorities; [Petty]: Government Officials; [Petty]: Judicial; [Petty]: Education; [Petty]: Documents; [Petty]: Health
Jamaica	2010	LAPOP	1473	1504	[Grand]: Government Officials; [Petty]: Authorities; [Petty]: Government Officials; [Petty]: Judicial; [Petty]: Education; [Petty]: Documents; [Petty]: Health
Jamaica	2012	LAPOP	1463	1500	[Grand]: Government Officials; [Petty]: Authorities; [Petty]: Government Officials; [Petty]: Judicial; [Petty]: Education; [Petty]: Documents; [Petty]: Health
Jamaica	2014	LAPOP	1461	1503	[Grand]: Government Officials; [Petty]: Authorities; [Petty]: Government Officials; [Petty]: Judicial; [Petty]: Education; [Petty]: Documents; [Petty]: Health
Jamaica	2017	LAPOP	1355	1513	[Grand]: Politician; [Petty]: Authorities; [Petty]: Government Officials; [Petty]: Judicial; [Petty]: Education; [Petty]: Documents; [Petty]: Health
Jamaica	2019	LAPOP	1411	1512	[Grand]: Politician; [Grand]: Government Officials; [Petty]: Authorities; [Petty]: Government Officials; [Petty]: Judicial; [Petty]: Education; [Petty]: Documents; [Petty]: Health
Japan	2006	International Social Survey Programme	1156	1123	[Grand]: Politician; [Grand]: Government Officials; [Petty]: Government Officials

Japan	2016	Asiabarometers	993	0	[Grand]: State; [Grand]: Local Government
Japan	2019	Asiabarometers	971	0	[Grand]: State; [Grand]: Local Government
Japan	2019	World Value Surveys	1263	1244	[Grand]: State; [Grand]: Civil; [Grand]: Severity; [Grand]: Local Government; [Petty]: Government Officials; [Petty]: Election
Jordan	2011	Arabbarometers	1065	0	[Grand]: State
Jordan	2014	Arabbarometers	1687	0	[Grand]: State
Jordan	2014	World Value Surveys	1200	1105	[Grand]: State; [Petty]: Election
Jordan	2017	Arabbarometers	1166	0	[Grand]: State
Jordan	2018	World Value Surveys	1169	1191	[Grand]: State; [Grand]: Civil; [Grand]: Severity; [Grand]: Local Government; [Petty]: Government Officials; [Petty]: Election
Jordan	2021	Arabbarometers	2914	0	[Grand]: State
Kazakhstan	2011	World Value Surveys	0	1077	[Petty]: Election
Kazakhstan	2018	World Value Surveys	1251	1151	[Grand]: State; [Grand]: Civil; [Grand]: Severity; [Grand]: Local Government; [Petty]: Government Officials; [Petty]: Election
Kenya	2008	Afrobarometers	1069	960	[Grand]: Executive; [Grand]: Legislative; [Grand]: Judicial; [Grand]: Taxation; [Grand]: Government Officials; [Grand]: Authorities; [Grand]: Local Government; [Petty]: Authorities; [Petty]: Utilities; [Petty]: Documents
Kenya	2012	Afrobarometers	2310	2381	[Grand]: Executive; [Grand]: Legislative; [Grand]: Judicial; [Grand]: Taxation; [Grand]: Government Officials; [Grand]: Authorities; [Grand]: Local Government; [Petty]: Authorities; [Petty]: Utilities; [Petty]: Education; [Petty]: Documents; [Petty]: Health; [Petty]: Election
Kenya	2014	Afrobarometers	2377	2382	[Grand]: Executive; [Grand]: Legislative; [Grand]: Judicial; [Grand]: Taxation; [Grand]: Government Officials; [Grand]: Authorities; [Grand]: Local Government; [Petty]: Authorities; [Petty]: Utilities; [Petty]: Education; [Petty]: Documents; [Petty]: Health; [Petty]: Election
Kenya	2016	Afrobarometers	1531	1599	[Grand]: Executive; [Grand]: Legislative; [Grand]: Judicial; [Grand]: Government Officials; [Grand]: Authorities; [Grand]: Local Government; [Petty]: Authorities; [Petty]: Education; [Petty]: Documents; [Petty]: Health
Kenya	2019	Afrobarometers	2371	2194	[Grand]: Executive; [Grand]: Legislative; [Grand]: Judicial; [Grand]: Taxation; [Grand]: Civil; [Grand]: Authorities; [Grand]: Local Government; [Petty]: Authorities; [Petty]: Education; [Petty]: Documents; [Petty]: Health
Kiribati	2017	Transparency International	498	484	[Grand]: Executive; [Grand]: Legislative; [Grand]: State; [Grand]: Judicial; [Grand]: Civil; [Grand]: Authorities; [Grand]: Local Government; [Petty]: Authorities; [Petty]: Judicial; [Petty]: Utilities; [Petty]: Education; [Petty]: Documents; [Petty]: Health; [Petty]: Election
Kosovo	2009	World Bank Enterprise Surveys	242	249	[Grand]: State; [Grand]: Taxation; [Petty]: Government Officials; [Petty]: Utilities; [Petty]: Documents
Kosovo	2013	World Bank Enterprise Surveys	191	186	[Grand]: State; [Grand]: Taxation; [Petty]: Government Officials; [Petty]: Utilities; [Petty]: Documents
Kuwait	2014	Arabbarometers	968	0	[Grand]: State
Kuwait	2014	World Value Surveys	1089	900	[Grand]: State; [Petty]: Election
Kyrgyzstan	2011	World Value Surveys	0	1349	[Petty]: Election
Kyrgyzstan	2020	World Value Surveys	1181	1170	[Grand]: State; [Grand]: Civil; [Grand]: Severity; [Grand]: Local Government; [Petty]: Government Officials; [Petty]: Election
Lao	2012	World Bank Enterprise Surveys	252	230	[Grand]: State; [Grand]: Taxation; [Petty]: Government Officials; [Petty]: Utilities; [Petty]: Documents
Lao	2016	World Bank Enterprise Surveys	316	225	[Grand]: State; [Grand]: Taxation; [Petty]: Government Officials; [Petty]: Utilities; [Petty]: Documents
Latvia	1996	World Value Surveys	1137	0	[Grand]: Severity
Latvia	2006	International Social Survey Programme	925	894	[Grand]: Politician; [Grand]: Government Officials; [Petty]: Government Officials
Latvia	2007	Eurobarometers	1009	1009	[Grand]: State; [Grand]: Judicial; [Grand]: Government Officials; [Grand]: Authorities; [Grand]: Local Government; [Petty]: Authorities; [Petty]: Government Officials; [Petty]: Judicial; [Petty]: Education; [Petty]: Health
Latvia	2011	Eurobarometers	1014	1014	[Grand]: State; [Grand]: Judicial; [Grand]: Government Officials; [Grand]: Authorities; [Grand]: Local Government; [Petty]: Authorities; [Petty]: Government Officials; [Petty]: Judicial; [Petty]: Education; [Petty]: Health

Latvia	2017	Eurobarometers	1001	1001	[Grand]: State; [Grand]: Judicial; [Grand]: Taxation; [Grand]: Government Officials; [Grand]: Authorities; [Grand]: Local Government; [Petty]: Authorities; [Petty]: Government Officials; [Petty]: Judicial; [Petty]: Education; [Petty]: Health
Latvia	2019	Eurobarometers	1002	1002	[Grand]: State; [Grand]: Judicial; [Grand]: Taxation; [Grand]: Government Officials; [Grand]: Authorities; [Grand]: Local Government; [Petty]: Authorities; [Petty]: Government Officials; [Petty]: Judicial; [Petty]: Education; [Petty]: Health
Latvia	2021	Europe Quality of Government QoG	878	1023	[Grand]: Authorities; [Petty]: Authorities; [Petty]: Education; [Petty]: Health
Latvia	2022	Eurobarometers	1013	1013	[Grand]: State; [Grand]: Judicial; [Grand]: Taxation; [Grand]: Government Officials; [Grand]: Authorities; [Grand]: Local Government; [Petty]: Authorities; [Petty]: Government Officials; [Petty]: Judicial; [Petty]: Education; [Petty]: Health
Lebanon	2011	Arabbarometers	1379	0	[Grand]: State
Lebanon	2014	Arabbarometers	1187	0	[Grand]: State
Lebanon	2017	Arabbarometers	1197	0	[Grand]: State
Lebanon	2018	World Value Surveys	1200	1195	[Grand]: State; [Grand]: Civil; [Grand]: Severity; [Grand]: Local Government; [Petty]: Government Officials; [Petty]: Election
Lebanon	2021	Arabbarometers	2995	0	[Grand]: State
Lesotho	2008	Afrobarometers	1126	1146	[Grand]: Executive; [Grand]: Legislative; [Grand]: Judicial; [Grand]: Taxation; [Grand]: Government Officials; [Grand]: Authorities; [Grand]: Local Government; [Petty]: Authorities; [Petty]: Utilities; [Petty]: Documents
Lesotho	2012	Afrobarometers	1035	1189	[Grand]: Executive; [Grand]: Legislative; [Grand]: Judicial; [Grand]: Taxation; [Grand]: Government Officials; [Grand]: Authorities; [Grand]: Local Government; [Petty]: Authorities; [Petty]: Utilities; [Petty]: Education; [Petty]: Documents; [Petty]: Health; [Petty]: Election
Lesotho	2014	Afrobarometers	1071	1112	[Grand]: Executive; [Grand]: Legislative; [Grand]: Judicial; [Grand]: Taxation; [Grand]: Government Officials; [Grand]: Authorities; [Grand]: Local Government; [Petty]: Authorities; [Petty]: Utilities; [Petty]: Education; [Petty]: Documents; [Petty]: Health; [Petty]: Election
Lesotho	2017	Afrobarometers	1122	1200	[Grand]: Executive; [Grand]: Legislative; [Grand]: Judicial; [Grand]: Government Officials; [Grand]: Authorities; [Grand]: Local Government; [Petty]: Authorities; [Petty]: Education; [Petty]: Documents; [Petty]: Health
Lesotho	2020	Afrobarometers	1167	1066	[Grand]: Executive; [Grand]: Legislative; [Grand]: Judicial; [Grand]: Taxation; [Grand]: Civil; [Grand]: Authorities; [Grand]: Local Government; [Petty]: Authorities; [Petty]: Education; [Petty]: Documents; [Petty]: Health
Liberia	2008	Afrobarometers	1167	1115	[Grand]: Executive; [Grand]: Legislative; [Grand]: Judicial; [Grand]: Taxation; [Grand]: Government Officials; [Grand]: Authorities; [Grand]: Local Government; [Petty]: Authorities; [Petty]: Utilities; [Petty]: Documents
Liberia	2012	Afrobarometers	1150	1159	[Grand]: Executive; [Grand]: Legislative; [Grand]: Judicial; [Grand]: Taxation; [Grand]: Government Officials; [Grand]: Authorities; [Grand]: Local Government; [Petty]: Authorities; [Petty]: Utilities; [Petty]: Education; [Petty]: Documents; [Petty]: Health; [Petty]: Election
Liberia	2015	Afrobarometers	1187	1191	[Grand]: Executive; [Grand]: Legislative; [Grand]: Judicial; [Grand]: Taxation; [Grand]: Government Officials; [Grand]: Authorities; [Grand]: Local Government; [Petty]: Authorities; [Petty]: Utilities; [Petty]: Education; [Petty]: Documents; [Petty]: Health; [Petty]: Election
Liberia	2018	Afrobarometers	1189	1200	[Grand]: Executive; [Grand]: Legislative; [Grand]: Judicial; [Grand]: Government Officials; [Grand]: Authorities; [Grand]: Local Government; [Petty]: Authorities; [Petty]: Education; [Petty]: Documents; [Petty]: Health
Liberia	2020	Afrobarometers	1198	1096	[Grand]: Executive; [Grand]: Legislative; [Grand]: Judicial; [Grand]: Taxation; [Grand]: Civil; [Grand]: Authorities; [Grand]: Local Government; [Petty]: Authorities; [Petty]: Education; [Petty]: Documents; [Petty]: Health
Libya	2014	Arabbarometers	1189	0	[Grand]: State
Libya	2014	World Value Surveys	0	1447	[Petty]: Election
Libya	2021	Arabbarometers	2523	0	[Grand]: State
Lithuania	1997	World Value Surveys	950	0	[Grand]: Severity
Lithuania	2007	Eurobarometers	1018	1018	[Grand]: State; [Grand]: Judicial; [Grand]: Government Officials; [Grand]: Authorities; [Grand]: Local Government; [Petty]: Authorities; [Petty]: Government Officials; [Petty]: Judicial; [Petty]: Education; [Petty]: Health

Lithuania	2011	Eurobarometers	1031	1031	[Grand]: State; [Grand]: Judicial; [Grand]: Government Officials; [Grand]: Authorities; [Grand]: Local Government; [Petty]: Authorities; [Petty]: Government Officials; [Petty]: Judicial; [Petty]: Education; [Petty]: Health
Lithuania	2017	Eurobarometers	1008	1008	[Grand]: State; [Grand]: Judicial; [Grand]: Taxation; [Grand]: Government Officials; [Grand]: Authorities; [Grand]: Local Government; [Petty]: Authorities; [Petty]: Government Officials; [Petty]: Judicial; [Petty]: Education; [Petty]: Health
Lithuania	2019	Eurobarometers	1000	1000	[Grand]: State; [Grand]: Judicial; [Grand]: Taxation; [Grand]: Government Officials; [Grand]: Authorities; [Grand]: Local Government; [Petty]: Authorities; [Petty]: Government Officials; [Petty]: Judicial; [Petty]: Education; [Petty]: Health
Lithuania	2021	Europe Quality of Government QoG	1811	2013	[Grand]: Authorities; [Petty]: Authorities; [Petty]: Education; [Petty]: Health
Lithuania	2022	Eurobarometers	1006	1006	[Grand]: State; [Grand]: Judicial; [Grand]: Taxation; [Grand]: Government Officials; [Grand]: Authorities; [Grand]: Local Government; [Petty]: Authorities; [Petty]: Government Officials; [Petty]: Judicial; [Petty]: Education; [Petty]: Health
Luxembourg	2011	Eurobarometers	502	502	[Grand]: State; [Grand]: Judicial; [Grand]: Government Officials; [Grand]: Authorities; [Grand]: Local Government; [Petty]: Authorities; [Petty]: Government Officials; [Petty]: Judicial; [Petty]: Education; [Petty]: Health
Luxembourg	2017	Eurobarometers	508	508	[Grand]: State; [Grand]: Judicial; [Grand]: Government Officials; [Grand]: Authorities; [Grand]: Local Government; [Petty]: Authorities; [Petty]: Government Officials; [Petty]: Judicial; [Petty]: Education; [Petty]: Health
Luxembourg	2019	Eurobarometers	509	509	[Grand]: State; [Grand]: Judicial; [Grand]: Government Officials; [Grand]: Authorities; [Grand]: Local Government; [Petty]: Authorities; [Petty]: Government Officials; [Petty]: Judicial; [Petty]: Education; [Petty]: Health
Luxembourg	2022	Eurobarometers	506	506	[Grand]: State; [Grand]: Judicial; [Grand]: Government Officials; [Grand]: Authorities; [Grand]: Local Government; [Petty]: Authorities; [Petty]: Government Officials; [Petty]: Judicial; [Petty]: Education; [Petty]: Health
Madagascar	2012	Afrobarometers	993	1174	[Grand]: Executive; [Grand]: Judicial; [Grand]: Taxation; [Grand]: Government Officials; [Grand]: Authorities; [Grand]: Local Government; [Petty]: Authorities; [Petty]: Utilities; [Petty]: Education; [Petty]: Documents; [Petty]: Health; [Petty]: Election
Madagascar	2014	Afrobarometers	1193	1197	[Grand]: Executive; [Grand]: Legislative; [Grand]: Judicial; [Grand]: Taxation; [Grand]: Government Officials; [Grand]: Authorities; [Grand]: Local Government; [Petty]: Authorities; [Petty]: Utilities; [Petty]: Education; [Petty]: Documents; [Petty]: Health; [Petty]: Election
Madagascar	2018	Afrobarometers	1178	1200	[Grand]: Executive; [Grand]: Legislative; [Grand]: Judicial; [Grand]: Government Officials; [Grand]: Authorities; [Grand]: Local Government; [Petty]: Authorities; [Petty]: Education; [Petty]: Documents; [Petty]: Health
Malawi	2016	Afrobarometers	1156	1200	[Grand]: Executive; [Grand]: Legislative; [Grand]: Judicial; [Grand]: Government Officials; [Grand]: Authorities; [Grand]: Local Government; [Petty]: Authorities; [Petty]: Education; [Petty]: Documents; [Petty]: Health
Malaysia	2010	Asiabarometers	1425	0	[Grand]: State; [Grand]: Local Government
Malaysia	2012	World Value Surveys	0	1056	[Petty]: Election
Malaysia	2018	World Value Surveys	1313	1313	[Grand]: State; [Grand]: Civil; [Grand]: Severity; [Grand]: Local Government; [Petty]: Government Officials; [Petty]: Election
Maldives	2018	World Bank Country Group Surveys	105	0	[Grand]: State
Maldives	2021	World Bank Country Group Surveys	84	0	[Grand]: State
Mali	2008	Afrobarometers	1164	904	[Grand]: Executive; [Grand]: Legislative; [Grand]: Judicial; [Grand]: Taxation; [Grand]: Government Officials; [Grand]: Authorities; [Grand]: Local Government; [Petty]: Authorities; [Petty]: Utilities; [Petty]: Documents
Mali	2012	Afrobarometers	1161	1198	[Grand]: Executive; [Grand]: Legislative; [Grand]: Judicial; [Grand]: Taxation; [Grand]: Government Officials; [Grand]: Authorities; [Grand]: Local Government; [Petty]: Authorities; [Petty]: Utilities; [Petty]: Education; [Petty]: Documents; [Petty]: Health; [Petty]: Election
Mali	2014	Afrobarometers	1199	1198	[Grand]: Executive; [Grand]: Legislative; [Grand]: Judicial; [Grand]: Taxation; [Grand]: Government Officials; [Grand]: Authorities; [Grand]: Local Government; [Petty]: Authorities; [Petty]: Utilities; [Petty]: Education; [Petty]: Documents; [Petty]: Health; [Petty]: Election

Mali	2017	Afrobarometers	1180	1200	[Grand]: Executive; [Grand]: Legislative; [Grand]: Judicial; [Grand]: Government Officials; [Grand]: Authorities; [Grand]: Local Government; [Petty]: Authorities; [Petty]: Education; [Petty]: Documents; [Petty]: Health
Mali	2020	Afrobarometers	1186	961	[Grand]: Executive; [Grand]: Legislative; [Grand]: Judicial; [Grand]: Taxation; [Grand]: Civil; [Grand]: Authorities; [Grand]: Local Government; [Petty]: Authorities; [Petty]: Education; [Petty]: Documents; [Petty]: Health
Malta	2021	Europe Quality of Government QoG	461	504	[Grand]: Authorities; [Petty]: Authorities; [Petty]: Education; [Petty]: Health
Marshall Islands	2017	Transparency International	260	258	[Grand]: Executive; [Grand]: Legislative; [Grand]: State; [Grand]: Judicial; [Grand]: Civil; [Grand]: Authorities; [Grand]: Local Government; [Petty]: Authorities; [Petty]: Judicial; [Petty]: Utilities; [Petty]: Education; [Petty]: Documents; [Petty]: Health; [Petty]: Election
Mauritania	2015	World Bank Country Group Surveys	172	0	[Grand]: State
Mauritania	2018	World Bank Country Group Surveys	294	0	[Grand]: State
Mauritania	2021	World Bank Country Group Surveys	347	0	[Grand]: State
Mauritius	2012	Afrobarometers	1164	1199	[Grand]: Executive; [Grand]: Legislative; [Grand]: Judicial; [Grand]: Taxation; [Grand]: Government Officials; [Grand]: Authorities; [Grand]: Local Government; [Petty]: Authorities; [Petty]: Utilities; [Petty]: Education; [Petty]: Documents; [Petty]: Health; [Petty]: Election
Mauritius	2014	Afrobarometers	1158	1189	[Grand]: Executive; [Grand]: Legislative; [Grand]: Judicial; [Grand]: Taxation; [Grand]: Government Officials; [Grand]: Authorities; [Grand]: Local Government; [Petty]: Authorities; [Petty]: Utilities; [Petty]: Education; [Petty]: Documents; [Petty]: Health; [Petty]: Election
Mauritius	2017	Afrobarometers	1111	1200	[Grand]: Executive; [Grand]: Legislative; [Grand]: Judicial; [Grand]: Government Officials; [Grand]: Authorities; [Grand]: Local Government; [Petty]: Authorities; [Petty]: Education; [Petty]: Documents; [Petty]: Health
Mauritius	2020	Afrobarometers	1160	1080	[Grand]: Executive; [Grand]: Legislative; [Grand]: Judicial; [Grand]: Taxation; [Grand]: Civil; [Grand]: Authorities; [Grand]: Local Government; [Petty]: Authorities; [Petty]: Education; [Petty]: Documents; [Petty]: Health
Mexico	2001	Latinobarometers	1252	0	[Grand]: Civil; [Grand]: Severity
Mexico	2004	LAPOP	1365	1551	[Grand]: Government Officials; [Petty]: Authorities; [Petty]: Government Officials; [Petty]: Judicial; [Petty]: Education; [Petty]: Documents; [Petty]: Health
Mexico	2006	LAPOP	1435	1557	[Grand]: Government Officials; [Petty]: Authorities; [Petty]: Government Officials; [Petty]: Judicial; [Petty]: Education; [Petty]: Documents; [Petty]: Health
Mexico	2008	LAPOP	1489	1560	[Grand]: Government Officials; [Petty]: Authorities; [Petty]: Government Officials; [Petty]: Judicial; [Petty]: Education; [Petty]: Documents; [Petty]: Health
Mexico	2010	LAPOP	1512	1557	[Grand]: Government Officials; [Petty]: Authorities; [Petty]: Government Officials; [Petty]: Judicial; [Petty]: Education; [Petty]: Documents; [Petty]: Health
Mexico	2012	LAPOP	1510	1536	[Grand]: Government Officials; [Petty]: Authorities; [Petty]: Government Officials; [Petty]: Judicial; [Petty]: Education; [Petty]: Documents; [Petty]: Health
Mexico	2013	Latinobarometers	1157	0	[Grand]: State; [Grand]: Local Government
Mexico	2014	LAPOP	1485	1535	[Grand]: Government Officials; [Petty]: Authorities; [Petty]: Government Officials; [Petty]: Judicial; [Petty]: Education; [Petty]: Documents; [Petty]: Health
Mexico	2016	Latinobarometers	1146	0	[Grand]: Executive; [Grand]: Legislative; [Grand]: Judicial; [Grand]: Taxation; [Grand]: Government Officials; [Grand]: Authorities; [Grand]: Local Government
Mexico	2016	LAPOP	1499	1563	[Grand]: Politician; [Petty]: Authorities; [Petty]: Government Officials; [Petty]: Judicial; [Petty]: Education; [Petty]: Documents; [Petty]: Health
Mexico	2017	Latinobarometers	1187	0	[Grand]: Legislative; [Grand]: State; [Grand]: Judicial; [Grand]: Local Government
Mexico	2018	Latinobarometers	1130	0	[Grand]: Executive; [Grand]: Legislative; [Grand]: Judicial; [Grand]: Taxation; [Grand]: Government Officials; [Grand]: Authorities; [Grand]: Local Government
Mexico	2019	LAPOP	1510	1580	[Grand]: Politician; [Grand]: Government Officials; [Petty]: Authorities; [Petty]: Government Officials; [Petty]: Judicial; [Petty]: Education; [Petty]: Documents; [Petty]: Health

Moldova	1996	World Value Surveys	905	0	[Grand]: Severity
Mongolia	2014	Asiabarometers	1197	0	[Grand]: State; [Grand]: Local Government
Mongolia	2018	Asiabarometers	1205	0	[Grand]: State; [Grand]: Local Government
Montenegro	2009	World Bank Enterprise Surveys	72	113	[Grand]: State; [Grand]: Taxation; [Petty]: Government Officials; [Petty]: Utilities; [Petty]: Documents
Montenegro	2013	World Bank Enterprise Surveys	104	126	[Grand]: Taxation; [Petty]: Government Officials; [Petty]: Utilities; [Petty]: Documents
Morocco	2012	Afrobarometers	1124	1190	[Grand]: Legislative; [Grand]: Judicial; [Grand]: Taxation; [Grand]: Government Officials; [Grand]: Authorities; [Grand]: Local Government; [Petty]: Authorities; [Petty]: Utilities; [Petty]: Education; [Petty]: Documents; [Petty]: Health; [Petty]: Election
Morocco	2014	Arabbarometers	1028	0	[Grand]: State
Morocco	2015	Afrobarometers	1152	1189	[Grand]: Executive; [Grand]: Legislative; [Grand]: Judicial; [Grand]: Taxation; [Grand]: Government Officials; [Grand]: Authorities; [Grand]: Local Government; [Petty]: Authorities; [Petty]: Utilities; [Petty]: Education; [Petty]: Documents; [Petty]: Health; [Petty]: Election
Morocco	2017	Arabbarometers	1133	0	[Grand]: State
Morocco	2018	Afrobarometers	1107	1192	[Grand]: Executive; [Grand]: Legislative; [Grand]: Judicial; [Grand]: Government Officials; [Grand]: Authorities; [Grand]: Local Government; [Petty]: Authorities; [Petty]: Education; [Petty]: Documents; [Petty]: Health
Morocco	2021	Arabbarometers	2950	0	[Grand]: State
Mozambique	2018	Afrobarometers	2071	2392	[Grand]: Executive; [Grand]: Legislative; [Grand]: Judicial; [Grand]: Government Officials; [Grand]: Authorities; [Grand]: Local Government; [Petty]: Authorities; [Petty]: Education; [Petty]: Documents; [Petty]: Health
Mozambique	2021	Afrobarometers	1060	886	[Grand]: Executive; [Grand]: Legislative; [Grand]: Judicial; [Grand]: Taxation; [Grand]: Civil; [Grand]: Authorities; [Grand]: Local Government; [Petty]: Authorities; [Petty]: Education; [Petty]: Documents; [Petty]: Health
Myanmar	2020	World Value Surveys	1200	1200	[Grand]: State; [Grand]: Civil; [Grand]: Severity; [Grand]: Local Government; [Petty]: Government Officials; [Petty]: Election
Namibia	2008	Afrobarometers	1168	865	[Grand]: Executive; [Grand]: Legislative; [Grand]: Judicial; [Grand]: Taxation; [Grand]: Government Officials; [Grand]: Authorities; [Grand]: Local Government; [Petty]: Authorities; [Petty]: Utilities; [Petty]: Documents
Namibia	2012	Afrobarometers	1197	1137	[Grand]: Executive; [Grand]: Legislative; [Grand]: Judicial; [Grand]: Taxation; [Grand]: Government Officials; [Grand]: Authorities; [Grand]: Local Government; [Petty]: Authorities; [Petty]: Utilities; [Petty]: Education; [Petty]: Documents; [Petty]: Health; [Petty]: Election
Namibia	2014	Afrobarometers	1179	1179	[Grand]: Executive; [Grand]: Legislative; [Grand]: Judicial; [Grand]: Taxation; [Grand]: Government Officials; [Grand]: Authorities; [Grand]: Local Government; [Petty]: Authorities; [Petty]: Utilities; [Petty]: Education; [Petty]: Documents; [Petty]: Health; [Petty]: Election
Namibia	2017	Afrobarometers	1138	1200	[Grand]: Executive; [Grand]: Legislative; [Grand]: Judicial; [Grand]: Government Officials; [Grand]: Authorities; [Grand]: Local Government; [Petty]: Authorities; [Petty]: Education; [Petty]: Documents; [Petty]: Health
Namibia	2019	Afrobarometers	1163	938	[Grand]: Executive; [Grand]: Legislative; [Grand]: Judicial; [Grand]: Taxation; [Grand]: Civil; [Grand]: Authorities; [Grand]: Local Government; [Petty]: Authorities; [Petty]: Education; [Petty]: Documents; [Petty]: Health
Nauru	2017	Transparency International	86	100	[Grand]: Executive; [Grand]: Legislative; [Grand]: State; [Grand]: Judicial; [Grand]: Civil; [Grand]: Authorities; [Grand]: Local Government; [Petty]: Authorities; [Petty]: Judicial; [Petty]: Utilities; [Petty]: Education; [Petty]: Documents; [Petty]: Health; [Petty]: Election
Nepal	2009	World Bank Enterprise Surveys	252	360	[Grand]: State; [Grand]: Taxation; [Petty]: Government Officials; [Petty]: Utilities; [Petty]: Documents
Nepal	2013	World Bank Enterprise Surveys	360	463	[Grand]: State; [Grand]: Taxation; [Petty]: Government Officials; [Petty]: Utilities; [Petty]: Documents
Netherlands	2006	International Social Survey Programme	831	935	[Grand]: Politician; [Grand]: Government Officials; [Petty]: Government Officials
Netherlands	2007	Eurobarometers	1000	1000	[Grand]: State; [Grand]: Judicial; [Grand]: Government Officials; [Grand]: Authorities; [Grand]: Local Government; [Petty]: Authorities; [Petty]: Government Officials; [Petty]: Judicial; [Petty]: Education; [Petty]: Health
Netherlands	2011	Eurobarometers	1002	1002	[Grand]: State; [Grand]: Judicial; [Grand]: Government Officials; [Grand]: Authorities; [Grand]: Local Government; [Petty]: Authorities; [Petty]: Government Officials; [Petty]: Judicial; [Petty]: Education; [Petty]: Health

Netherlands	2012	World Value Surveys	0	1486	[Petty]: Election
Netherlands	2013	Europe Quality of Government QoG	4445	4814	[Grand]: Authorities; [Petty]: Authorities; [Petty]: Education; [Petty]: Health; [Petty]: Election
Netherlands	2017	Europe Quality of Government QoG	1788	1840	[Grand]: Authorities; [Petty]: Authorities; [Petty]: Education; [Petty]: Health; [Petty]: Election
Netherlands	2017	Eurobarometers	1025	1025	[Grand]: State; [Grand]: Judicial; [Grand]: Taxation; [Grand]: Government Officials; [Grand]: Authorities; [Grand]: Local Government; [Petty]: Authorities; [Petty]: Government Officials; [Petty]: Judicial; [Petty]: Education; [Petty]: Health
Netherlands	2019	Eurobarometers	1032	1032	[Grand]: State; [Grand]: Judicial; [Grand]: Taxation; [Grand]: Government Officials; [Grand]: Authorities; [Grand]: Local Government; [Petty]: Authorities; [Petty]: Government Officials; [Petty]: Judicial; [Petty]: Education; [Petty]: Health
Netherlands	2021	Europe Quality of Government QoG	5324	6088	[Grand]: Authorities; [Petty]: Authorities; [Petty]: Education; [Petty]: Health
Netherlands	2022	Eurobarometers	1003	1003	[Grand]: State; [Grand]: Judicial; [Grand]: Taxation; [Grand]: Government Officials; [Grand]: Authorities; [Grand]: Local Government; [Petty]: Authorities; [Petty]: Government Officials; [Petty]: Judicial; [Petty]: Education; [Petty]: Health
New Caledonia	2017	Transparency International	480	488	[Grand]: Executive; [Grand]: Legislative; [Grand]: State; [Grand]: Judicial; [Grand]: Civil; [Grand]: Authorities; [Grand]: Local Government; [Petty]: Authorities; [Petty]: Judicial; [Petty]: Utilities; [Petty]: Education; [Petty]: Documents; [Petty]: Health; [Petty]: Election
New Zealand	2006	International Social Survey Programme	1116	1163	[Grand]: Politician; [Grand]: Government Officials; [Petty]: Government Officials
New Zealand	2020	World Value Surveys	943	981	[Grand]: State; [Grand]: Civil; [Grand]: Severity; [Grand]: Local Government; [Petty]: Government Officials; [Petty]: Election
Nicaragua	1998	Latinobarometers	994	0	[Grand]: Severity
Nicaragua	2000	Latinobarometers	997	0	[Grand]: Severity
Nicaragua	2001	Latinobarometers	1005	0	[Grand]: Civil; [Grand]: Severity
Nicaragua	2004	LAPOP	1225	1430	[Grand]: Government Officials; [Petty]: Authorities; [Petty]: Government Officials; [Petty]: Judicial; [Petty]: Education; [Petty]: Documents; [Petty]: Health
Nicaragua	2006	LAPOP	1679	1757	[Grand]: Government Officials; [Petty]: Authorities; [Petty]: Government Officials; [Petty]: Judicial; [Petty]: Education; [Petty]: Documents; [Petty]: Health
Nicaragua	2008	LAPOP	1455	1536	[Grand]: Government Officials; [Petty]: Authorities; [Petty]: Government Officials; [Petty]: Judicial; [Petty]: Education; [Petty]: Documents; [Petty]: Health
Nicaragua	2010	LAPOP	1448	1539	[Grand]: Government Officials; [Petty]: Authorities; [Petty]: Government Officials; [Petty]: Judicial; [Petty]: Education; [Petty]: Documents; [Petty]: Health
Nicaragua	2012	LAPOP	1585	1686	[Grand]: Government Officials; [Petty]: Authorities; [Petty]: Government Officials; [Petty]: Judicial; [Petty]: Education; [Petty]: Documents; [Petty]: Health
Nicaragua	2013	Latinobarometers	851	0	[Grand]: State; [Grand]: Local Government
Nicaragua	2014	LAPOP	1476	1546	[Grand]: Government Officials; [Petty]: Authorities; [Petty]: Government Officials; [Petty]: Judicial; [Petty]: Education; [Petty]: Documents; [Petty]: Health
Nicaragua	2016	Latinobarometers	936	0	[Grand]: Executive; [Grand]: Legislative; [Grand]: Judicial; [Grand]: Taxation; [Grand]: Government Officials; [Grand]: Authorities; [Grand]: Local Government
Nicaragua	2016	LAPOP	1399	1559	[Grand]: Politician; [Petty]: Authorities; [Petty]: Government Officials; [Petty]: Judicial; [Petty]: Education; [Petty]: Documents; [Petty]: Health
Nicaragua	2017	Latinobarometers	951	0	[Grand]: Legislative; [Grand]: State; [Grand]: Judicial; [Grand]: Local Government
Nicaragua	2018	Latinobarometers	871	0	[Grand]: Executive; [Grand]: Legislative; [Grand]: Judicial; [Grand]: Taxation; [Grand]: Government Officials; [Grand]: Authorities; [Grand]: Local Government
Nicaragua	2019	LAPOP	1435	1547	[Grand]: Politician; [Grand]: Government Officials; [Petty]: Authorities; [Petty]: Government Officials; [Petty]: Judicial; [Petty]: Education; [Petty]: Documents; [Petty]: Health
Nicaragua	2020	World Value Surveys	1200	1200	[Grand]: State; [Grand]: Civil; [Grand]: Severity; [Grand]: Local Government; [Petty]: Government Officials; [Petty]: Election

Niger	2012	Afrobarometers	1102	1144	[Grand]: Executive; [Grand]: Legislative; [Grand]: Judicial; [Grand]: Taxation; [Grand]: Government Officials; [Grand]: Authorities; [Grand]: Local Government; [Petty]: Authorities; [Petty]: Utilities; [Petty]: Education; [Petty]: Documents; [Petty]: Health; [Petty]: Election
Niger	2015	Afrobarometers	1162	1178	[Grand]: Executive; [Grand]: Legislative; [Grand]: Judicial; [Grand]: Taxation; [Grand]: Government Officials; [Grand]: Authorities; [Grand]: Local Government; [Petty]: Authorities; [Petty]: Utilities; [Petty]: Education; [Petty]: Documents; [Petty]: Health; [Petty]: Election
Niger	2018	Afrobarometers	1142	1200	[Grand]: Executive; [Grand]: Legislative; [Grand]: Judicial; [Grand]: Government Officials; [Grand]: Authorities; [Grand]: Local Government; [Petty]: Authorities; [Petty]: Education; [Petty]: Documents; [Petty]: Health
Niger	2020	Afrobarometers	1144	1134	[Grand]: Executive; [Grand]: Legislative; [Grand]: Judicial; [Grand]: Taxation; [Grand]: Civil; [Grand]: Authorities; [Grand]: Local Government; [Petty]: Authorities; [Petty]: Education; [Petty]: Documents; [Petty]: Health
Nigeria	2008	Afrobarometers	2250	1970	[Grand]: Executive; [Grand]: Legislative; [Grand]: Judicial; [Grand]: Taxation; [Grand]: Government Officials; [Grand]: Authorities; [Grand]: Local Government; [Petty]: Authorities; [Petty]: Utilities; [Petty]: Documents
Nigeria	2011	World Value Surveys	0	1653	[Petty]: Election
Nigeria	2012	Afrobarometers	2390	2368	[Grand]: Executive; [Grand]: Legislative; [Grand]: Judicial; [Grand]: Taxation; [Grand]: Government Officials; [Grand]: Authorities; [Grand]: Local Government; [Petty]: Authorities; [Petty]: Utilities; [Petty]: Education; [Petty]: Documents; [Petty]: Health; [Petty]: Election
Nigeria	2014	Afrobarometers	2383	2374	[Grand]: Executive; [Grand]: Legislative; [Grand]: Judicial; [Grand]: Taxation; [Grand]: Government Officials; [Grand]: Authorities; [Grand]: Local Government; [Petty]: Authorities; [Petty]: Utilities; [Petty]: Education; [Petty]: Documents; [Petty]: Health; [Petty]: Election
Nigeria	2017	Afrobarometers	1592	1600	[Grand]: Executive; [Grand]: Legislative; [Grand]: Judicial; [Grand]: Government Officials; [Grand]: Authorities; [Grand]: Local Government; [Petty]: Authorities; [Petty]: Education; [Petty]: Documents; [Petty]: Health
Nigeria	2018	World Value Surveys	1236	1235	[Grand]: State; [Grand]: Civil; [Grand]: Severity; [Grand]: Local Government; [Petty]: Government Officials; [Petty]: Election
Nigeria	2020	Afrobarometers	1501	1199	[Grand]: Executive; [Grand]: Legislative; [Grand]: Judicial; [Grand]: Taxation; [Grand]: Civil; [Grand]: Authorities; [Grand]: Local Government; [Petty]: Authorities; [Petty]: Education; [Petty]: Documents; [Petty]: Health
North Macedonia	1998	World Value Surveys	900	0	[Grand]: Severity
Norway	2006	International Social Survey Programme	1180	1264	[Grand]: Politician; [Grand]: Government Officials; [Petty]: Government Officials
PNG	2017	Transparency International	1025	1025	[Grand]: Executive; [Grand]: Legislative; [Grand]: State; [Grand]: Judicial; [Grand]: Civil; [Grand]: Authorities; [Grand]: Local Government; [Petty]: Authorities; [Petty]: Judicial; [Petty]: Utilities; [Petty]: Education; [Petty]: Documents; [Petty]: Health; [Petty]: Election
Pakistan	2012	World Value Surveys	0	1127	[Petty]: Election
Pakistan	2018	World Value Surveys	1971	1958	[Grand]: State; [Grand]: Civil; [Grand]: Severity; [Grand]: Local Government; [Petty]: Government Officials; [Petty]: Election
Palau	2017	Transparency International	237	252	[Grand]: Executive; [Grand]: Legislative; [Grand]: State; [Grand]: Judicial; [Grand]: Civil; [Grand]: Authorities; [Grand]: Local Government; [Petty]: Authorities; [Petty]: Judicial; [Petty]: Utilities; [Petty]: Education; [Petty]: Documents; [Petty]: Health; [Petty]: Election
Palestine	2011	Arabbarometers	1054	0	[Grand]: State
Palestine	2013	World Value Surveys	0	856	[Petty]: Election
Palestine	2014	Arabbarometers	1114	0	[Grand]: State
Palestine	2017	Arabbarometers	1148	0	[Grand]: State
Panama	2000	Latinobarometers	992	0	[Grand]: Severity
Panama	2001	Latinobarometers	984	0	[Grand]: Civil; [Grand]: Severity
Panama	2006	LAPOP	1453	1535	[Grand]: Government Officials; [Petty]: Authorities; [Petty]: Government Officials; [Petty]: Judicial; [Petty]: Education; [Petty]: Documents; [Petty]: Health
Panama	2008	LAPOP	1480	1535	[Grand]: Government Officials; [Petty]: Authorities; [Petty]: Government Officials; [Petty]: Judicial; [Petty]: Education; [Petty]: Documents; [Petty]: Health

Panama	2010	LAPOP	1478	1536	[Grand]: Government Officials; [Petty]: Authorities; [Petty]: Government Officials; [Petty]: Judicial; [Petty]: Education; [Petty]: Documents; [Petty]: Health
Panama	2012	LAPOP	1562	1617	[Grand]: Government Officials; [Petty]: Authorities; [Petty]: Government Officials; [Petty]: Judicial; [Petty]: Education; [Petty]: Documents; [Petty]: Health
Panama	2013	Latinobarometers	971	0	[Grand]: State; [Grand]: Local Government
Panama	2014	LAPOP	1456	1506	[Grand]: Government Officials; [Petty]: Authorities; [Petty]: Government Officials; [Petty]: Judicial; [Petty]: Education; [Petty]: Documents; [Petty]: Health
Panama	2017	Latinobarometers	975	0	[Grand]: Legislative; [Grand]: State; [Grand]: Judicial; [Grand]: Local Government
Panama	2018	Latinobarometers	887	0	[Grand]: Executive; [Grand]: Legislative; [Grand]: Judicial; [Grand]: Taxation; [Grand]: Government Officials; [Grand]: Authorities; [Grand]: Local Government
Paraguay	1998	Latinobarometers	595	0	[Grand]: Severity
Paraguay	2000	Latinobarometers	601	0	[Grand]: Severity
Paraguay	2001	Latinobarometers	604	0	[Grand]: Civil; [Grand]: Severity
Paraguay	2006	LAPOP	0	1161	[Petty]: Authorities; [Petty]: Government Officials; [Petty]: Judicial; [Petty]: Education; [Petty]: Documents; [Petty]: Health
Paraguay	2008	LAPOP	1102	1166	[Grand]: Government Officials; [Petty]: Authorities; [Petty]: Government Officials; [Petty]: Judicial; [Petty]: Education; [Petty]: Documents; [Petty]: Health
Paraguay	2010	LAPOP	1415	1500	[Grand]: Government Officials; [Petty]: Authorities; [Petty]: Government Officials; [Petty]: Judicial; [Petty]: Education; [Petty]: Documents; [Petty]: Health
Paraguay	2012	LAPOP	1412	1507	[Grand]: Government Officials; [Petty]: Authorities; [Petty]: Government Officials; [Petty]: Judicial; [Petty]: Education; [Petty]: Documents; [Petty]: Health
Paraguay	2014	LAPOP	1425	1496	[Grand]: Government Officials; [Petty]: Authorities; [Petty]: Government Officials; [Petty]: Judicial; [Petty]: Education; [Petty]: Documents; [Petty]: Health
Paraguay	2016	LAPOP	1486	1528	[Grand]: Politician; [Grand]: Government Officials; [Petty]: Authorities; [Petty]: Government Officials; [Petty]: Judicial; [Petty]: Education; [Petty]: Documents; [Petty]: Health
Paraguay	2019	LAPOP	1508	1512	[Grand]: Politician; [Grand]: Government Officials; [Petty]: Authorities; [Petty]: Government Officials; [Petty]: Judicial; [Petty]: Education; [Petty]: Documents; [Petty]: Health
Peru	1996	World Value Surveys	1129	0	[Grand]: Severity
Peru	1998	Latinobarometers	1031	0	[Grand]: Severity
Peru	2000	Latinobarometers	1038	0	[Grand]: Severity
Peru	2001	Latinobarometers	1016	0	[Grand]: Civil; [Grand]: Severity
Peru	2006	LAPOP	1454	1500	[Grand]: Government Officials; [Petty]: Authorities; [Petty]: Government Officials; [Petty]: Judicial; [Petty]: Education; [Petty]: Documents; [Petty]: Health
Peru	2008	LAPOP	1458	1443	[Grand]: Government Officials; [Petty]: Authorities; [Petty]: Government Officials; [Petty]: Judicial; [Petty]: Education; [Petty]: Documents; [Petty]: Health
Peru	2010	LAPOP	1446	1499	[Grand]: Government Officials; [Petty]: Authorities; [Petty]: Government Officials; [Petty]: Judicial; [Petty]: Education; [Petty]: Documents; [Petty]: Health
Peru	2012	World Value Surveys	0	1089	[Petty]: Election
Peru	2012	LAPOP	1463	1499	[Grand]: Government Officials; [Petty]: Authorities; [Petty]: Government Officials; [Petty]: Judicial; [Petty]: Education; [Petty]: Documents; [Petty]: Health
Peru	2013	Latinobarometers	1139	0	[Grand]: State; [Grand]: Local Government
Peru	2014	LAPOP	1448	1500	[Grand]: Government Officials; [Petty]: Authorities; [Petty]: Government Officials; [Petty]: Judicial; [Petty]: Education; [Petty]: Documents; [Petty]: Health
Peru	2016	Latinobarometers	1150	0	[Grand]: Executive; [Grand]: Legislative; [Grand]: Judicial; [Grand]: Taxation; [Grand]: Government Officials; [Grand]: Authorities; [Grand]: Local Government

Peru	2017	LAPOP	2630	2643	[Grand]: Politician; [Grand]: Government Officials; [Petty]: Authorities; [Petty]: Government Officials; [Petty]: Judicial; [Petty]: Education; [Petty]: Documents; [Petty]: Health
Peru	2017	Latinobarometers	1178	0	[Grand]: Legislative; [Grand]: State; [Grand]: Judicial; [Grand]: Local Government
Peru	2018	World Value Surveys	1398	1387	[Grand]: State; [Grand]: Civil; [Grand]: Severity; [Grand]: Local Government; [Petty]: Government Officials; [Petty]: Election
Peru	2018	Latinobarometers	1140	0	[Grand]: Executive; [Grand]: Legislative; [Grand]: Judicial; [Grand]: Taxation; [Grand]: Government Officials; [Grand]: Authorities; [Grand]: Local Government
Peru	2019	LAPOP	1506	1520	[Grand]: Politician; [Grand]: Government Officials; [Petty]: Authorities; [Petty]: Government Officials; [Petty]: Judicial; [Petty]: Education; [Petty]: Documents; [Petty]: Health
Philippines	2012	World Value Surveys	0	1197	[Petty]: Election
Philippines	2019	World Value Surveys	1200	1200	[Grand]: State; [Grand]: Civil; [Grand]: Severity; [Grand]: Local Government; [Petty]: Government Officials; [Petty]: Election
Poland	2006	International Social Survey Programme	1181	1206	[Grand]: Politician; [Grand]: Government Officials; [Petty]: Government Officials
Poland	2007	Eurobarometers	1000	1000	[Grand]: State; [Grand]: Judicial; [Grand]: Government Officials; [Grand]: Authorities; [Grand]: Local Government; [Petty]: Authorities; [Petty]: Government Officials; [Petty]: Judicial; [Petty]: Education; [Petty]: Health
Poland	2010	Europe Quality of Government QoG	3120	3120	[Grand]: Authorities; [Petty]: Authorities; [Petty]: Education; [Petty]: Health; [Petty]: Election
Poland	2011	Eurobarometers	1000	1000	[Grand]: State; [Grand]: Judicial; [Grand]: Government Officials; [Grand]: Authorities; [Grand]: Local Government; [Petty]: Authorities; [Petty]: Government Officials; [Petty]: Judicial; [Petty]: Education; [Petty]: Health
Poland	2012	World Value Surveys	0	798	[Petty]: Election
Poland	2013	Europe Quality of Government QoG	5844	6396	[Grand]: Authorities; [Petty]: Authorities; [Petty]: Education; [Petty]: Health; [Petty]: Election
Poland	2017	Europe Quality of Government QoG	6059	6441	[Grand]: Authorities; [Petty]: Authorities; [Petty]: Education; [Petty]: Health; [Petty]: Election
Poland	2017	Eurobarometers	1037	1037	[Grand]: State; [Grand]: Judicial; [Grand]: Taxation; [Grand]: Government Officials; [Grand]: Authorities; [Grand]: Local Government; [Petty]: Authorities; [Petty]: Government Officials; [Petty]: Judicial; [Petty]: Education; [Petty]: Health
Poland	2019	Eurobarometers	1034	1034	[Grand]: State; [Grand]: Judicial; [Grand]: Taxation; [Grand]: Government Officials; [Grand]: Authorities; [Grand]: Local Government; [Petty]: Authorities; [Petty]: Government Officials; [Petty]: Judicial; [Petty]: Education; [Petty]: Health
Poland	2021	Europe Quality of Government QoG	8876	10197	[Grand]: Authorities; [Petty]: Authorities; [Petty]: Education; [Petty]: Health
Poland	2022	Eurobarometers	1009	1009	[Grand]: State; [Grand]: Judicial; [Grand]: Taxation; [Grand]: Government Officials; [Grand]: Authorities; [Grand]: Local Government; [Petty]: Authorities; [Petty]: Government Officials; [Petty]: Judicial; [Petty]: Education; [Petty]: Health
Portugal	2006	International Social Survey Programme	1654	1765	[Grand]: Politician; [Grand]: Government Officials; [Petty]: Government Officials
Portugal	2007	Eurobarometers	1000	1000	[Grand]: State; [Grand]: Judicial; [Grand]: Government Officials; [Grand]: Authorities; [Grand]: Local Government; [Petty]: Authorities; [Petty]: Government Officials; [Petty]: Judicial; [Petty]: Education; [Petty]: Health
Portugal	2010	Europe Quality of Government QoG	1365	1365	[Grand]: Authorities; [Petty]: Authorities; [Petty]: Education; [Petty]: Health; [Petty]: Election
Portugal	2011	Eurobarometers	1035	1035	[Grand]: State; [Grand]: Judicial; [Grand]: Government Officials; [Grand]: Authorities; [Grand]: Local Government; [Petty]: Authorities; [Petty]: Government Officials; [Petty]: Judicial; [Petty]: Education; [Petty]: Health
Portugal	2013	Europe Quality of Government QoG	2686	2885	[Grand]: Authorities; [Petty]: Authorities; [Petty]: Education; [Petty]: Health; [Petty]: Election
Portugal	2017	Europe Quality of Government QoG	2620	2800	[Grand]: Authorities; [Petty]: Authorities; [Petty]: Education; [Petty]: Health; [Petty]: Election
Portugal	2017	Eurobarometers	1099	1099	[Grand]: State; [Grand]: Judicial; [Grand]: Taxation; [Grand]: Government Officials; [Grand]: Authorities; [Grand]: Local Government; [Petty]: Authorities; [Petty]: Government Officials; [Petty]: Judicial; [Petty]: Education; [Petty]: Health

Portugal	2019	Eurobarometers	1003	1003	[Grand]: State; [Grand]: Judicial; [Grand]: Taxation; [Grand]: Government Officials; [Grand]: Authorities; [Grand]: Local Government; [Petty]: Authorities; [Petty]: Government Officials; [Petty]: Judicial; [Petty]: Education; [Petty]: Health
Portugal	2021	Europe Quality of Government QoG	3039	3553	[Grand]: Authorities; [Petty]: Authorities; [Petty]: Education; [Petty]: Health
Portugal	2022	Eurobarometers	1005	1005	[Grand]: State; [Grand]: Judicial; [Grand]: Taxation; [Grand]: Government Officials; [Grand]: Authorities; [Grand]: Local Government; [Petty]: Authorities; [Petty]: Government Officials; [Petty]: Judicial; [Petty]: Education; [Petty]: Health
PuertoRico	1995	World Value Surveys	1098	0	[Grand]: Severity
PuertoRico	2018	World Value Surveys	1124	1124	[Grand]: State; [Grand]: Civil; [Grand]: Severity; [Grand]: Local Government; [Petty]: Government Officials; [Petty]: Election
Romania	2007	Eurobarometers	1000	1000	[Grand]: State; [Grand]: Judicial; [Grand]: Government Officials; [Grand]: Authorities; [Grand]: Local Government; [Petty]: Authorities; [Petty]: Government Officials; [Petty]: Judicial; [Petty]: Education; [Petty]: Health
Romania	2010	Europe Quality of Government QoG	1560	1560	[Grand]: Authorities; [Petty]: Authorities; [Petty]: Education; [Petty]: Health; [Petty]: Election
Romania	2011	Eurobarometers	1050	1050	[Grand]: State; [Grand]: Judicial; [Grand]: Government Officials; [Grand]: Authorities; [Grand]: Local Government; [Petty]: Authorities; [Petty]: Government Officials; [Petty]: Judicial; [Petty]: Education; [Petty]: Health
Romania	2012	World Value Surveys	0	1253	[Petty]: Election
Romania	2013	Europe Quality of Government QoG	3188	3200	[Grand]: Authorities; [Petty]: Authorities; [Petty]: Education; [Petty]: Health; [Petty]: Election
Romania	2017	Europe Quality of Government QoG	3570	3600	[Grand]: Authorities; [Petty]: Authorities; [Petty]: Education; [Petty]: Health; [Petty]: Election
Romania	2017	Eurobarometers	1055	1055	[Grand]: State; [Grand]: Judicial; [Grand]: Taxation; [Grand]: Government Officials; [Grand]: Authorities; [Grand]: Local Government; [Petty]: Authorities; [Petty]: Government Officials; [Petty]: Judicial; [Petty]: Education; [Petty]: Health
Romania	2018	World Value Surveys	1221	1142	[Grand]: State; [Grand]: Civil; [Grand]: Severity; [Grand]: Local Government; [Petty]: Government Officials; [Petty]: Election
Romania	2019	Eurobarometers	1081	1081	[Grand]: State; [Grand]: Judicial; [Grand]: Taxation; [Grand]: Government Officials; [Grand]: Authorities; [Grand]: Local Government; [Petty]: Authorities; [Petty]: Government Officials; [Petty]: Judicial; [Petty]: Education; [Petty]: Health
Romania	2021	Europe Quality of Government QoG	3883	4121	[Grand]: Authorities; [Petty]: Authorities; [Petty]: Education; [Petty]: Health
Romania	2022	Eurobarometers	1037	1037	[Grand]: State; [Grand]: Judicial; [Grand]: Taxation; [Grand]: Government Officials; [Grand]: Authorities; [Grand]: Local Government; [Petty]: Authorities; [Petty]: Government Officials; [Petty]: Judicial; [Petty]: Education; [Petty]: Health
RussianFederation	1995	World Value Surveys	1862	0	[Grand]: Severity
RussianFederation	2006	International Social Survey Programme	1951	1967	[Grand]: Politician; [Grand]: Government Officials; [Petty]: Government Officials
RussianFederation	2017	World Value Surveys	1769	1730	[Grand]: State; [Grand]: Civil; [Grand]: Severity; [Grand]: Local Government; [Petty]: Government Officials; [Petty]: Election
Rwanda	2012	World Value Surveys	0	713	[Petty]: Election
Samoa	2017	Transparency International	497	504	[Grand]: Executive; [Grand]: Legislative; [Grand]: State; [Grand]: Judicial; [Grand]: Civil; [Grand]: Authorities; [Petty]: Authorities; [Petty]: Judicial; [Petty]: Utilities; [Petty]: Education; [Petty]: Documents; [Petty]: Health; [Petty]: Election
Senegal	2008	Afrobarometers	924	740	[Grand]: Executive; [Grand]: Legislative; [Grand]: Judicial; [Grand]: Taxation; [Grand]: Government Officials; [Grand]: Authorities; [Grand]: Local Government; [Petty]: Authorities; [Petty]: Utilities; [Petty]: Documents
Senegal	2012	Afrobarometers	1081	1199	[Grand]: Executive; [Grand]: Legislative; [Grand]: Judicial; [Grand]: Taxation; [Grand]: Government Officials; [Grand]: Authorities; [Grand]: Local Government; [Petty]: Authorities; [Petty]: Utilities; [Petty]: Education; [Petty]: Documents; [Petty]: Health; [Petty]: Election
Senegal	2014	Afrobarometers	1079	1173	[Grand]: Executive; [Grand]: Legislative; [Grand]: Judicial; [Grand]: Taxation; [Grand]: Government Officials; [Grand]: Authorities; [Grand]: Local Government; [Petty]: Authorities; [Petty]: Utilities; [Petty]: Education; [Petty]: Documents; [Petty]: Health; [Petty]: Election

Senegal	2017	Afrobarometers	1004	1200	[Grand]: Executive; [Grand]: Legislative; [Grand]: Judicial; [Grand]: Government Officials; [Grand]: Authorities; [Grand]: Local Government; [Petty]: Authorities; [Petty]: Education; [Petty]: Documents; [Petty]: Health
Senegal	2020	Afrobarometers	1158	1088	[Grand]: Executive; [Grand]: Legislative; [Grand]: Judicial; [Grand]: Taxation; [Grand]: Civil; [Grand]: Authorities; [Grand]: Local Government; [Petty]: Authorities; [Petty]: Education; [Petty]: Documents; [Petty]: Health
Serbia	2017	World Value Surveys	1004	994	[Grand]: State; [Grand]: Civil; [Grand]: Severity; [Grand]: Local Government; [Petty]: Government Officials; [Petty]: Election
Seychelles	2020	World Bank Country Group Surveys	105	0	[Grand]: State
Sierra Leone	2018	Afrobarometers	1137	1200	[Grand]: Executive; [Grand]: Legislative; [Grand]: Judicial; [Grand]: Government Officials; [Grand]: Authorities; [Grand]: Local Government; [Petty]: Authorities; [Petty]: Education; [Petty]: Documents; [Petty]: Health
Singapore	2014	Asiabarometers	940	0	[Grand]: State; [Grand]: Local Government
Slovakia	1998	World Value Surveys	1008	0	[Grand]: Severity
Slovakia	2006	International Social Survey Programme	1051	993	[Grand]: Politician; [Grand]: Government Officials; [Petty]: Government Officials
Slovakia	2007	Eurobarometers	1055	1055	[Grand]: State; [Grand]: Judicial; [Grand]: Government Officials; [Grand]: Authorities; [Grand]: Local Government; [Petty]: Authorities; [Petty]: Government Officials; [Petty]: Judicial; [Petty]: Education; [Petty]: Health
Slovakia	2010	Europe Quality of Government QoG	780	780	[Grand]: Authorities; [Petty]: Authorities; [Petty]: Education; [Petty]: Health; [Petty]: Election
Slovakia	2011	Eurobarometers	1013	1013	[Grand]: State; [Grand]: Judicial; [Grand]: Government Officials; [Grand]: Authorities; [Grand]: Local Government; [Petty]: Authorities; [Petty]: Government Officials; [Petty]: Judicial; [Petty]: Education; [Petty]: Health
Slovakia	2013	Europe Quality of Government QoG	1609	1609	[Grand]: Authorities; [Petty]: Authorities; [Petty]: Education; [Petty]: Health; [Petty]: Election
Slovakia	2017	Europe Quality of Government QoG	1799	1800	[Grand]: Authorities; [Petty]: Authorities; [Petty]: Education; [Petty]: Health; [Petty]: Election
Slovakia	2017	Eurobarometers	1080	1080	[Grand]: State; [Grand]: Judicial; [Grand]: Taxation; [Grand]: Government Officials; [Grand]: Authorities; [Grand]: Local Government; [Petty]: Authorities; [Petty]: Government Officials; [Petty]: Judicial; [Petty]: Education; [Petty]: Health
Slovakia	2019	Eurobarometers	1038	1038	[Grand]: State; [Grand]: Judicial; [Grand]: Taxation; [Grand]: Government Officials; [Grand]: Authorities; [Grand]: Local Government; [Petty]: Authorities; [Petty]: Government Officials; [Petty]: Judicial; [Petty]: Education; [Petty]: Health
Slovakia	2021	Europe Quality of Government QoG	1776	2056	[Grand]: Authorities; [Petty]: Authorities; [Petty]: Education; [Petty]: Health
Slovakia	2022	Eurobarometers	1009	1009	[Grand]: State; [Grand]: Judicial; [Grand]: Taxation; [Grand]: Government Officials; [Grand]: Authorities; [Grand]: Local Government; [Petty]: Authorities; [Petty]: Government Officials; [Petty]: Judicial; [Petty]: Education; [Petty]: Health
Slovenia	2006	International Social Survey Programme	885	941	[Grand]: Politician; [Grand]: Government Officials; [Petty]: Government Officials
Slovenia	2007	Eurobarometers	1016	1016	[Grand]: State; [Grand]: Judicial; [Grand]: Government Officials; [Grand]: Authorities; [Grand]: Local Government; [Petty]: Authorities; [Petty]: Government Officials; [Petty]: Judicial; [Petty]: Education; [Petty]: Health
Slovenia	2011	Eurobarometers	1024	1024	[Grand]: State; [Grand]: Judicial; [Grand]: Government Officials; [Grand]: Authorities; [Grand]: Local Government; [Petty]: Authorities; [Petty]: Government Officials; [Petty]: Judicial; [Petty]: Education; [Petty]: Health
Slovenia	2017	Eurobarometers	1014	1014	[Grand]: State; [Grand]: Judicial; [Grand]: Taxation; [Grand]: Government Officials; [Grand]: Authorities; [Grand]: Local Government; [Petty]: Authorities; [Petty]: Government Officials; [Petty]: Judicial; [Petty]: Education; [Petty]: Health
Slovenia	2019	Eurobarometers	1006	1006	[Grand]: State; [Grand]: Judicial; [Grand]: Taxation; [Grand]: Government Officials; [Grand]: Authorities; [Grand]: Local Government; [Petty]: Authorities; [Petty]: Government Officials; [Petty]: Judicial; [Petty]: Education; [Petty]: Health
Slovenia	2021	Europe Quality of Government QoG	933	1011	[Grand]: Authorities; [Petty]: Authorities; [Petty]: Education; [Petty]: Health

Slovenia	2022	Eurobarometers	1006	1006	[Grand]: State; [Grand]: Judicial; [Grand]: Taxation; [Grand]: Government Officials; [Grand]: Authorities; [Grand]: Local Government; [Petty]: Authorities; [Petty]: Government Officials; [Petty]: Judicial; [Petty]: Education; [Petty]: Health
Solomon Islands	2017	Transparency International	528	527	[Grand]: Executive; [Grand]: Legislative; [Grand]: State; [Grand]: Judicial; [Grand]: Civil; [Grand]: Authorities; [Petty]: Authorities; [Petty]: Judicial; [Petty]: Utilities; [Petty]: Education; [Petty]: Documents; [Petty]: Health; [Petty]: Election
Somalia	2021	World Bank Country Group Surveys	163	0	[Grand]: State
South Africa	1996	World Value Surveys	2488	0	[Grand]: Severity
South Africa	2006	International Social Survey Programme	2744	2694	[Grand]: Politician; [Grand]: Government Officials; [Petty]: Government Officials
South Africa	2008	Afrobarometers	2238	1649	[Grand]: Executive; [Grand]: Legislative; [Grand]: Judicial; [Grand]: Taxation; [Grand]: Government Officials; [Grand]: Authorities; [Grand]: Local Government; [Petty]: Authorities; [Petty]: Utilities; [Petty]: Documents
South Africa	2012	Afrobarometers	2371	2378	[Grand]: Executive; [Grand]: Legislative; [Grand]: Judicial; [Grand]: Taxation; [Grand]: Government Officials; [Grand]: Authorities; [Grand]: Local Government; [Petty]: Authorities; [Petty]: Utilities; [Petty]: Education; [Petty]: Documents; [Petty]: Health; [Petty]: Election
South Africa	2013	World Value Surveys	0	2791	[Petty]: Election
South Africa	2014	Afrobarometers	2381	2285	[Grand]: Executive; [Grand]: Legislative; [Grand]: Judicial; [Grand]: Taxation; [Grand]: Government Officials; [Grand]: Authorities; [Grand]: Local Government; [Petty]: Authorities; [Petty]: Utilities; [Petty]: Education; [Petty]: Documents; [Petty]: Health; [Petty]: Election
South Africa	2018	Afrobarometers	1794	1840	[Grand]: Executive; [Grand]: Legislative; [Grand]: Judicial; [Grand]: Government Officials; [Grand]: Authorities; [Grand]: Local Government; [Petty]: Authorities; [Petty]: Education; [Petty]: Documents; [Petty]: Health
South Africa	2021	Afrobarometers	1551	1227	[Grand]: Executive; [Grand]: Legislative; [Grand]: Judicial; [Grand]: Taxation; [Grand]: Civil; [Grand]: Authorities; [Grand]: Local Government; [Petty]: Authorities; [Petty]: Education; [Petty]: Documents; [Petty]: Health
South Korea	1996	World Value Surveys	1216	0	[Grand]: Severity
South Korea	2006	International Social Survey Programme	1578	1575	[Grand]: Politician; [Grand]: Government Officials; [Petty]: Government Officials
South Korea	2015	Asiabarometers	1189	0	[Grand]: State; [Grand]: Local Government
South Korea	2018	World Value Surveys	1245	1245	[Grand]: State; [Grand]: Civil; [Grand]: Severity; [Grand]: Local Government; [Petty]: Government Officials; [Petty]: Election
South Korea	2019	Asiabarometers	1263	0	[Grand]: State; [Grand]: Local Government
South Sudan	2013	World Bank Country Group Surveys	187	0	[Grand]: State
Spain	1995	World Value Surveys	1125	0	[Grand]: Severity
Spain	2006	International Social Survey Programme	2274	2472	[Grand]: Politician; [Grand]: Government Officials; [Petty]: Government Officials
Spain	2007	Eurobarometers	1000	1000	[Grand]: State; [Grand]: Judicial; [Grand]: Government Officials; [Grand]: Authorities; [Grand]: Local Government; [Petty]: Authorities; [Petty]: Government Officials; [Petty]: Judicial; [Petty]: Education; [Petty]: Health
Spain	2010	Europe Quality of Government QoG	3315	3315	[Grand]: Authorities; [Petty]: Authorities; [Petty]: Education; [Petty]: Health; [Petty]: Election
Spain	2011	Eurobarometers	1004	1004	[Grand]: State; [Grand]: Judicial; [Grand]: Government Officials; [Grand]: Authorities; [Grand]: Local Government; [Petty]: Authorities; [Petty]: Government Officials; [Petty]: Judicial; [Petty]: Education; [Petty]: Health
Spain	2013	Europe Quality of Government QoG	6598	6800	[Grand]: Authorities; [Petty]: Authorities; [Petty]: Education; [Petty]: Health; [Petty]: Election
Spain	2017	Europe Quality of Government QoG	6826	6992	[Grand]: Authorities; [Petty]: Authorities; [Petty]: Education; [Petty]: Health; [Petty]: Election
Spain	2017	Eurobarometers	1016	1016	[Grand]: State; [Grand]: Judicial; [Grand]: Taxation; [Grand]: Government Officials; [Grand]: Authorities; [Grand]: Local Government; [Petty]: Authorities; [Petty]: Government Officials; [Petty]: Judicial; [Petty]: Education; [Petty]: Health

Spain	2019	Eurobarometers	1014	1014	[Grand]: State; [Grand]: Judicial; [Grand]: Taxation; [Grand]: Government Officials; [Grand]: Authorities; [Grand]: Local Government; [Petty]: Authorities; [Petty]: Government Officials; [Petty]: Judicial; [Petty]: Education; [Petty]: Health
Spain	2021	Europe Quality of Government QoG	9215	10337	[Grand]: Authorities; [Petty]: Authorities; [Petty]: Education; [Petty]: Health
Spain	2022	Eurobarometers	1003	1003	[Grand]: State; [Grand]: Judicial; [Grand]: Taxation; [Grand]: Government Officials; [Grand]: Authorities; [Grand]: Local Government; [Petty]: Authorities; [Petty]: Government Officials; [Petty]: Judicial; [Petty]: Education; [Petty]: Health
Sri Lanka	2011	World Bank Enterprise Surveys	323	577	[Grand]: State; [Grand]: Taxation; [Petty]: Government Officials; [Petty]: Utilities; [Petty]: Documents
Sudan	2012	Afrobarometers	1030	1184	[Grand]: Executive; [Grand]: Legislative; [Grand]: Judicial; [Grand]: Taxation; [Grand]: Government Officials; [Grand]: Authorities; [Grand]: Local Government; [Petty]: Authorities; [Petty]: Utilities; [Petty]: Education; [Petty]: Documents; [Petty]: Health; [Petty]: Election
Sudan	2015	Afrobarometers	1151	1169	[Grand]: Executive; [Grand]: Legislative; [Grand]: Judicial; [Grand]: Taxation; [Grand]: Government Officials; [Grand]: Authorities; [Grand]: Local Government; [Petty]: Authorities; [Petty]: Utilities; [Petty]: Education; [Petty]: Documents; [Petty]: Health; [Petty]: Election
Suriname	2014	LAPOP	0	3996	[Petty]: Authorities; [Petty]: Government Officials; [Petty]: Judicial; [Petty]: Education; [Petty]: Documents; [Petty]: Health
Sweden	2006	International Social Survey Programme	956	1097	[Grand]: Politician; [Grand]: Government Officials; [Petty]: Government Officials
Sweden	2007	Eurobarometers	1015	1015	[Grand]: State; [Grand]: Judicial; [Grand]: Government Officials; [Grand]: Authorities; [Grand]: Local Government; [Petty]: Authorities; [Petty]: Government Officials; [Petty]: Judicial; [Petty]: Education; [Petty]: Health
Sweden	2011	Eurobarometers	1020	1020	[Grand]: State; [Grand]: Judicial; [Grand]: Government Officials; [Grand]: Authorities; [Grand]: Local Government; [Petty]: Authorities; [Petty]: Government Officials; [Petty]: Judicial; [Petty]: Education; [Petty]: Health
Sweden	2017	Eurobarometers	1051	1051	[Grand]: State; [Grand]: Judicial; [Grand]: Taxation; [Grand]: Government Officials; [Grand]: Authorities; [Grand]: Local Government; [Petty]: Authorities; [Petty]: Government Officials; [Petty]: Judicial; [Petty]: Education; [Petty]: Health
Sweden	2017	Europe Quality of Government QoG	1181	1200	[Grand]: Authorities; [Petty]: Authorities; [Petty]: Education; [Petty]: Health; [Petty]: Election
Sweden	2019	Eurobarometers	1012	1012	[Grand]: State; [Grand]: Judicial; [Grand]: Taxation; [Grand]: Government Officials; [Grand]: Authorities; [Grand]: Local Government; [Petty]: Authorities; [Petty]: Government Officials; [Petty]: Judicial; [Petty]: Education; [Petty]: Health
Sweden	2021	Europe Quality of Government QoG	2897	4002	[Grand]: Authorities; [Petty]: Authorities; [Petty]: Education; [Petty]: Health
Switzerland	1996	World Value Surveys	1104	0	[Grand]: Severity
Switzerland	2006	International Social Survey Programme	935	988	[Grand]: Politician; [Grand]: Government Officials; [Petty]: Government Officials
São Tomé and Príncipe	2018	Afrobarometers	1060	1200	[Grand]: Executive; [Grand]: Legislative; [Grand]: Judicial; [Grand]: Government Officials; [Grand]: Authorities; [Grand]: Local Government; [Petty]: Authorities; [Petty]: Education; [Petty]: Documents; [Petty]: Health
Tajikistan	2020	World Value Surveys	1200	1200	[Grand]: Severity; [Petty]: Government Officials; [Petty]: Election
Tanzania	2008	Afrobarometers	1109	941	[Grand]: Executive; [Grand]: Legislative; [Grand]: Judicial; [Grand]: Taxation; [Grand]: Government Officials; [Grand]: Authorities; [Grand]: Local Government; [Petty]: Authorities; [Petty]: Utilities; [Petty]: Documents
Tanzania	2012	Afrobarometers	2358	2391	[Grand]: Executive; [Grand]: Legislative; [Grand]: Judicial; [Grand]: Taxation; [Grand]: Government Officials; [Grand]: Authorities; [Grand]: Local Government; [Petty]: Authorities; [Petty]: Utilities; [Petty]: Education; [Petty]: Documents; [Petty]: Health; [Petty]: Election
Tanzania	2014	Afrobarometers	2295	2329	[Grand]: Executive; [Grand]: Legislative; [Grand]: Judicial; [Grand]: Taxation; [Grand]: Government Officials; [Grand]: Authorities; [Grand]: Local Government; [Petty]: Authorities; [Petty]: Utilities; [Petty]: Education; [Petty]: Documents; [Petty]: Health; [Petty]: Election
Tanzania	2017	Afrobarometers	2122	2400	[Grand]: Executive; [Grand]: Legislative; [Grand]: Judicial; [Grand]: Government Officials; [Grand]: Authorities; [Grand]: Local Government; [Petty]: Authorities; [Petty]: Education; [Petty]: Documents; [Petty]: Health

Tanzania	2021	Afrobarometers	2227	1931	[Grand]: Executive; [Grand]: Legislative; [Grand]: Judicial; [Grand]: Taxation; [Grand]: Civil; [Grand]: Authorities; [Grand]: Local Government; [Petty]: Authorities; [Petty]: Education; [Petty]: Documents; [Petty]: Health
Thailand	2013	World Value Surveys	0	1070	[Petty]: Election
Thailand	2014	Asiabarometers	1069	0	[Grand]: State; [Grand]: Local Government
Thailand	2018	World Value Surveys	1485	1491	[Grand]: State; [Grand]: Civil; [Grand]: Severity; [Grand]: Local Government; [Petty]: Government Officials; [Petty]: Election
Thailand	2018	Asiabarometers	1007	0	[Grand]: State; [Grand]: Local Government
Timor-Leste	2014		959	0	[Grand]: Severity
Timor-Leste	2016		1095	0	[Grand]: Executive; [Grand]: Legislative; [Grand]: State; [Grand]: Judicial; [Grand]: Politician; [Grand]: Authorities; [Grand]: Local Government
Timor-Leste	2018		1183	0	[Grand]: Executive; [Grand]: Legislative; [Grand]: State; [Grand]: Judicial; [Grand]: Politician; [Grand]: Authorities; [Grand]: Local Government
Togo	2012	Afrobarometers	1027	1195	[Grand]: Executive; [Grand]: Legislative; [Grand]: Judicial; [Grand]: Taxation; [Grand]: Government Officials; [Grand]: Authorities; [Grand]: Local Government; [Petty]: Authorities; [Petty]: Utilities; [Petty]: Education; [Petty]: Documents; [Petty]: Health; [Petty]: Election
Togo	2014	Afrobarometers	1086	1172	[Grand]: Executive; [Grand]: Legislative; [Grand]: Judicial; [Grand]: Taxation; [Grand]: Government Officials; [Grand]: Authorities; [Grand]: Local Government; [Petty]: Authorities; [Petty]: Utilities; [Petty]: Education; [Petty]: Documents; [Petty]: Health; [Petty]: Election
Togo	2017	Afrobarometers	1121	1200	[Grand]: Executive; [Grand]: Legislative; [Grand]: Judicial; [Grand]: Government Officials; [Grand]: Authorities; [Grand]: Local Government; [Petty]: Authorities; [Petty]: Education; [Petty]: Documents; [Petty]: Health
Togo	2020	Afrobarometers	1185	969	[Grand]: Executive; [Grand]: Legislative; [Grand]: Judicial; [Grand]: Taxation; [Grand]: Civil; [Grand]: Authorities; [Grand]: Local Government; [Petty]: Authorities; [Petty]: Education; [Petty]: Documents; [Petty]: Health
Tonga	2017	Transparency International	504	503	[Grand]: Executive; [Grand]: Legislative; [Grand]: State; [Grand]: Judicial; [Grand]: Civil; [Grand]: Authorities; [Grand]: Local Government; [Petty]: Authorities; [Petty]: Judicial; [Petty]: Utilities; [Petty]: Education; [Petty]: Documents; [Petty]: Health; [Petty]: Election
Tunisia	2011	Arabbarometers	1047	0	[Grand]: State
Tunisia	2012	Afrobarometers	1880	2385	[Grand]: Executive; [Grand]: Legislative; [Grand]: Judicial; [Grand]: Taxation; [Grand]: Government Officials; [Grand]: Authorities; [Grand]: Local Government; [Petty]: Authorities; [Petty]: Utilities; [Petty]: Education; [Petty]: Documents; [Petty]: Health; [Petty]: Election
Tunisia	2013	World Value Surveys	1192	717	[Grand]: State; [Petty]: Election
Tunisia	2014	Arabbarometers	1029	0	[Grand]: State
Tunisia	2017	Arabbarometers	1145	0	[Grand]: State
Tunisia	2018	Afrobarometers	1010	1199	[Grand]: Executive; [Grand]: Legislative; [Grand]: Judicial; [Grand]: Government Officials; [Grand]: Authorities; [Grand]: Local Government; [Petty]: Authorities; [Petty]: Education; [Petty]: Documents; [Petty]: Health
Tunisia	2019	World Value Surveys	1200	1182	[Grand]: State; [Grand]: Civil; [Grand]: Severity; [Grand]: Local Government; [Petty]: Government Officials; [Petty]: Election
Tunisia	2020	Afrobarometers	1139	802	[Grand]: Executive; [Grand]: Legislative; [Grand]: Judicial; [Grand]: Taxation; [Grand]: Civil; [Grand]: Authorities; [Grand]: Local Government; [Petty]: Authorities; [Petty]: Education; [Petty]: Documents; [Petty]: Health
Tunisia	2021	Arabbarometers	3119	0	[Grand]: State
Turkey	2018	World Value Surveys	2373	2383	[Grand]: State; [Grand]: Civil; [Grand]: Severity; [Grand]: Local Government; [Petty]: Government Officials; [Petty]: Election
Turkmenistan	2016	World Bank Country Group Surveys	53	0	[Grand]: State
Tuvalu	2017	Transparency International	158	157	[Grand]: Executive; [Grand]: Legislative; [Grand]: State; [Grand]: Judicial; [Grand]: Civil; [Grand]: Authorities; [Grand]: Local Government; [Petty]: Authorities; [Petty]: Judicial; [Petty]: Utilities; [Petty]: Education; [Petty]: Documents; [Petty]: Health; [Petty]: Election
Uganda	2008	Afrobarometers	2339	2170	[Grand]: Executive; [Grand]: Legislative; [Grand]: Judicial; [Grand]: Taxation; [Grand]: Government Officials; [Grand]: Authorities; [Grand]: Local Government; [Petty]: Authorities; [Petty]: Utilities; [Petty]: Documents

Uganda	2012	Afrobarometers	2366	2390	[Grand]: Executive; [Grand]: Legislative; [Grand]: Judicial; [Grand]: Taxation; [Grand]: Government Officials; [Grand]: Authorities; [Grand]: Local Government; [Petty]: Authorities; [Petty]: Utilities; [Petty]: Education; [Petty]: Documents; [Petty]: Health; [Petty]: Election
Uganda	2015	Afrobarometers	2330	2362	[Grand]: Executive; [Grand]: Legislative; [Grand]: Judicial; [Grand]: Taxation; [Grand]: Government Officials; [Grand]: Authorities; [Grand]: Local Government; [Petty]: Authorities; [Petty]: Utilities; [Petty]: Education; [Petty]: Documents; [Petty]: Health; [Petty]: Election
Uganda	2016	Afrobarometers	1184	1200	[Grand]: Executive; [Grand]: Legislative; [Grand]: Judicial; [Grand]: Government Officials; [Grand]: Authorities; [Grand]: Local Government; [Petty]: Authorities; [Petty]: Education; [Petty]: Documents; [Petty]: Health
Uganda	2019	Afrobarometers	851	798	[Grand]: Executive; [Grand]: Legislative; [Grand]: Judicial; [Grand]: Taxation; [Grand]: Civil; [Grand]: Authorities; [Grand]: Local Government; [Petty]: Authorities; [Petty]: Education; [Petty]: Documents; [Petty]: Health
Ukraine	1996	World Value Surveys	2539	0	[Grand]: Severity
Ukraine	2011	World Value Surveys	0	1217	[Petty]: Election
Ukraine	2020	World Value Surveys	1272	1254	[Grand]: State; [Grand]: Civil; [Grand]: Severity; [Grand]: Local Government; [Petty]: Government Officials; [Petty]: Election
UnitedKingdom	1998	World Value Surveys	1044	0	[Grand]: Severity
UnitedKingdom	2007	Eurobarometers	1278	1278	[Grand]: State; [Grand]: Judicial; [Grand]: Government Officials; [Grand]: Authorities; [Grand]: Local Government; [Petty]: Authorities; [Petty]: Government Officials; [Petty]: Judicial; [Petty]: Education; [Petty]: Health
UnitedKingdom	2011	Eurobarometers	1328	1328	[Grand]: State; [Grand]: Judicial; [Grand]: Government Officials; [Grand]: Authorities; [Grand]: Local Government; [Petty]: Authorities; [Petty]: Government Officials; [Petty]: Judicial; [Petty]: Education; [Petty]: Health
UnitedKingdom	2017	Eurobarometers	1382	1382	[Grand]: State; [Grand]: Judicial; [Grand]: Taxation; [Grand]: Government Officials; [Grand]: Authorities; [Grand]: Local Government; [Petty]: Authorities; [Petty]: Government Officials; [Petty]: Judicial; [Petty]: Education; [Petty]: Health
UnitedKingdom	2019	Eurobarometers	1023	1023	[Grand]: State; [Grand]: Judicial; [Grand]: Taxation; [Grand]: Government Officials; [Grand]: Authorities; [Grand]: Local Government; [Petty]: Authorities; [Petty]: Government Officials; [Petty]: Judicial; [Petty]: Education; [Petty]: Health
UnitedStates	1995	World Value Surveys	1323	0	[Grand]: Severity
UnitedStates	2006	International Social Survey Programme	1482	1513	[Grand]: Politician; [Grand]: Government Officials; [Petty]: Government Officials
UnitedStates	2008	LAPOP	1498	1500	[Grand]: Government Officials; [Petty]: Authorities; [Petty]: Government Officials; [Petty]: Judicial; [Petty]: Education; [Petty]: Documents; [Petty]: Health
UnitedStates	2017	World Value Surveys	2586	2585	[Grand]: State; [Grand]: Civil; [Grand]: Severity; [Grand]: Local Government; [Petty]: Government Officials; [Petty]: Election
Uruguay	1996	World Value Surveys	906	0	[Grand]: Severity
Uruguay	2000	Latinobarometers	1178	0	[Grand]: Severity
Uruguay	2001	Latinobarometers	1174	0	[Grand]: Civil; [Grand]: Severity
Uruguay	2007	LAPOP	1119	1197	[Grand]: Government Officials; [Petty]: Authorities; [Petty]: Government Officials; [Petty]: Judicial; [Petty]: Education; [Petty]: Documents; [Petty]: Health
Uruguay	2008	LAPOP	1406	1500	[Grand]: Government Officials; [Petty]: Authorities; [Petty]: Government Officials; [Petty]: Judicial; [Petty]: Education; [Petty]: Documents; [Petty]: Health
Uruguay	2010	LAPOP	1402	1500	[Grand]: Government Officials; [Petty]: Authorities; [Petty]: Government Officials; [Petty]: Judicial; [Petty]: Education; [Petty]: Documents; [Petty]: Health
Uruguay	2011	World Value Surveys	0	769	[Petty]: Election
Uruguay	2012	LAPOP	1394	1512	[Grand]: Government Officials; [Petty]: Authorities; [Petty]: Government Officials; [Petty]: Judicial; [Petty]: Education; [Petty]: Documents; [Petty]: Health
Uruguay	2013	Latinobarometers	1036	0	[Grand]: State; [Grand]: Local Government
Uruguay	2014	LAPOP	1417	1512	[Grand]: Government Officials; [Petty]: Authorities; [Petty]: Government Officials; [Petty]: Judicial; [Petty]: Education; [Petty]: Documents; [Petty]: Health

Uruguay	2016	Latinobarometers	1107	0	[Grand]: Executive; [Grand]: Legislative; [Grand]: Judicial; [Grand]: Taxation; [Grand]: Government Officials; [Grand]: Authorities; [Grand]: Local Government
Uruguay	2017	Latinobarometers	1140	0	[Grand]: Legislative; [Grand]: State; [Grand]: Judicial; [Grand]: Local Government
Uruguay	2018	Latinobarometers	1100	0	[Grand]: Executive; [Grand]: Legislative; [Grand]: Judicial; [Grand]: Taxation; [Grand]: Government Officials; [Grand]: Authorities; [Grand]: Local Government
Uzbekistan	2019	World Bank Country Group Surveys	352	0	[Grand]: State
Vanuatu	2017	Transparency International	510	515	[Grand]: Executive; [Grand]: Legislative; [Grand]: State; [Grand]: Judicial; [Grand]: Civil; [Grand]: Authorities; [Grand]: Local Government; [Petty]: Authorities; [Petty]: Judicial; [Petty]: Utilities; [Petty]: Education; [Petty]: Documents; [Petty]: Health; [Petty]: Election
Venezuela	2000	Latinobarometers	1194	0	[Grand]: Severity
Venezuela	2001	Latinobarometers	1191	0	[Grand]: Civil; [Grand]: Severity
Vietnam	2018	Asiabarometers	1044	0	[Grand]: State; [Grand]: Local Government
Vietnam	2020	World Value Surveys	1200	1200	[Grand]: State; [Grand]: Civil; [Grand]: Severity; [Grand]: Local Government; [Petty]: Government Officials; [Petty]: Election
Yemen	2011	Arabbarometers	1137	0	[Grand]: State
Yemen	2014	Arabbarometers	1137	0	[Grand]: State
Yemen	2014	World Value Surveys	928	759	[Grand]: State; [Petty]: Election
Zambia	2008	Afrobarometers	1106	1059	[Grand]: Executive; [Grand]: Legislative; [Grand]: Judicial; [Grand]: Taxation; [Grand]: Government Officials; [Grand]: Authorities; [Grand]: Local Government; [Petty]: Authorities; [Petty]: Utilities; [Petty]: Documents
Zambia	2012	Afrobarometers	1176	1197	[Grand]: Executive; [Grand]: Legislative; [Grand]: Judicial; [Grand]: Taxation; [Grand]: Government Officials; [Grand]: Authorities; [Grand]: Local Government; [Petty]: Authorities; [Petty]: Utilities; [Petty]: Education; [Petty]: Documents; [Petty]: Health; [Petty]: Election
Zambia	2014	Afrobarometers	1166	1177	[Grand]: Executive; [Grand]: Legislative; [Grand]: Judicial; [Grand]: Taxation; [Grand]: Government Officials; [Grand]: Authorities; [Grand]: Local Government; [Petty]: Authorities; [Petty]: Utilities; [Petty]: Education; [Petty]: Documents; [Petty]: Health; [Petty]: Election
Zambia	2017	Afrobarometers	1155	1200	[Grand]: Executive; [Grand]: Legislative; [Grand]: Judicial; [Grand]: Government Officials; [Grand]: Authorities; [Grand]: Local Government; [Petty]: Authorities; [Petty]: Education; [Petty]: Documents; [Petty]: Health
Zambia	2020	Afrobarometers	1169	1115	[Grand]: Executive; [Grand]: Legislative; [Grand]: Judicial; [Grand]: Taxation; [Grand]: Civil; [Grand]: Authorities; [Grand]: Local Government; [Petty]: Authorities; [Petty]: Education; [Petty]: Documents; [Petty]: Health
Zimbabwe	2008	Afrobarometers	1104	1048	[Grand]: Executive; [Grand]: Legislative; [Grand]: Judicial; [Grand]: Taxation; [Grand]: Government Officials; [Grand]: Authorities; [Grand]: Local Government; [Petty]: Authorities; [Petty]: Utilities; [Petty]: Documents
Zimbabwe	2012	Afrobarometers	2361	2374	[Grand]: Executive; [Grand]: Legislative; [Grand]: Judicial; [Grand]: Taxation; [Grand]: Government Officials; [Grand]: Authorities; [Grand]: Local Government; [Petty]: Authorities; [Petty]: Utilities; [Petty]: Education; [Petty]: Documents; [Petty]: Health; [Petty]: Election
Zimbabwe	2012	World Value Surveys	0	1144	[Petty]: Election
Zimbabwe	2014	Afrobarometers	2309	2312	[Grand]: Executive; [Grand]: Legislative; [Grand]: Judicial; [Grand]: Taxation; [Grand]: Government Officials; [Grand]: Authorities; [Grand]: Local Government; [Petty]: Authorities; [Petty]: Utilities; [Petty]: Education; [Petty]: Documents; [Petty]: Health; [Petty]: Election
Zimbabwe	2017	Afrobarometers	1150	1200	[Grand]: Executive; [Grand]: Legislative; [Grand]: Judicial; [Grand]: Government Officials; [Grand]: Authorities; [Grand]: Local Government; [Petty]: Authorities; [Petty]: Education; [Petty]: Documents; [Petty]: Health
Zimbabwe	2020	World Value Surveys	1214	1208	[Grand]: State; [Grand]: Civil; [Grand]: Severity; [Grand]: Local Government; [Petty]: Government Officials; [Petty]: Election
Zimbabwe	2021	Afrobarometers	1131	1021	[Grand]: Executive; [Grand]: Legislative; [Grand]: Judicial; [Grand]: Taxation; [Grand]: Civil; [Grand]: Authorities; [Grand]: Local Government; [Petty]: Authorities; [Petty]: Education; [Petty]: Documents; [Petty]: Health
